# Supplementary material for: Innate, translation‐dependent silencing of an invasive transposon in Arabidopsis
Source: EMBO Rep. 2021 Dec 21;23(3):e53400. doi: 10.15252/embr.202153400 (PMC8892269; doi:10.15252/embr.202153400)
Supplement: Supplementary file 1 — Appendix [file EMBR-23-e53400-s005.pdf]

# APPENDIX INFORMATION TO:

## Innate, translation-dependent silencing of an invasive transposon in Arabidopsis

*Stefan Oberlin, Rajendran Rajeswaran, Marieke Trasser, Verónica Barragán-Borrero, Michael A. Schon, Alexandra Plotnikova, Lukas Loncsek, Michael D. Nodine, Arturo Marí-Ordóñez* and Olivier Voinnet**

* For correspondence:

Olivier Voinnet: voinneto@ethz.ch

Arturo Marí-Ordóñez: arturo.mari-ordonez@gmi.oeaw.ac.at

## Table of contents:


- APPENDIX FIGURE S1 ---------------------------------- PAGE 3
- APPENDIX FIGURE S2 ---------------------------------- PAGE 4
- APPENDIX FIGURE S3 ---------------------------------- PAGE 6
- APPENDIX FIGURE S4 ---------------------------------- PAGE 8
- APPENDIX FIGURE S5 ---------------------------------- PAGE 9
- APPENDIX FIGURE S6 ---------------------------------- PAGE 10
- APPENDIX FIGURE S7 ---------------------------------- PAGE 12
- APPENDIX FIGURE S8 ---------------------------------- PAGE 13
- APPENDIX FIGURE S9 ---------------------------------- PAGE 14
- APPENDIX FIGURE S10 -------------------------------- PAGE 15
- APPENDIX FIGURE S11 -------------------------------- PAGE 17
- APPENDIX FIGURE S12 -------------------------------- PAGE 18
- APPENDIX DISCUSSION ------------------------------- PAGE 19
- APPENDIX FIGURE S13 -------------------------------- PAGE 23
- APPENDIX TABLE S1 ----------------------------------- PAGE 24

# Appendix figure S1

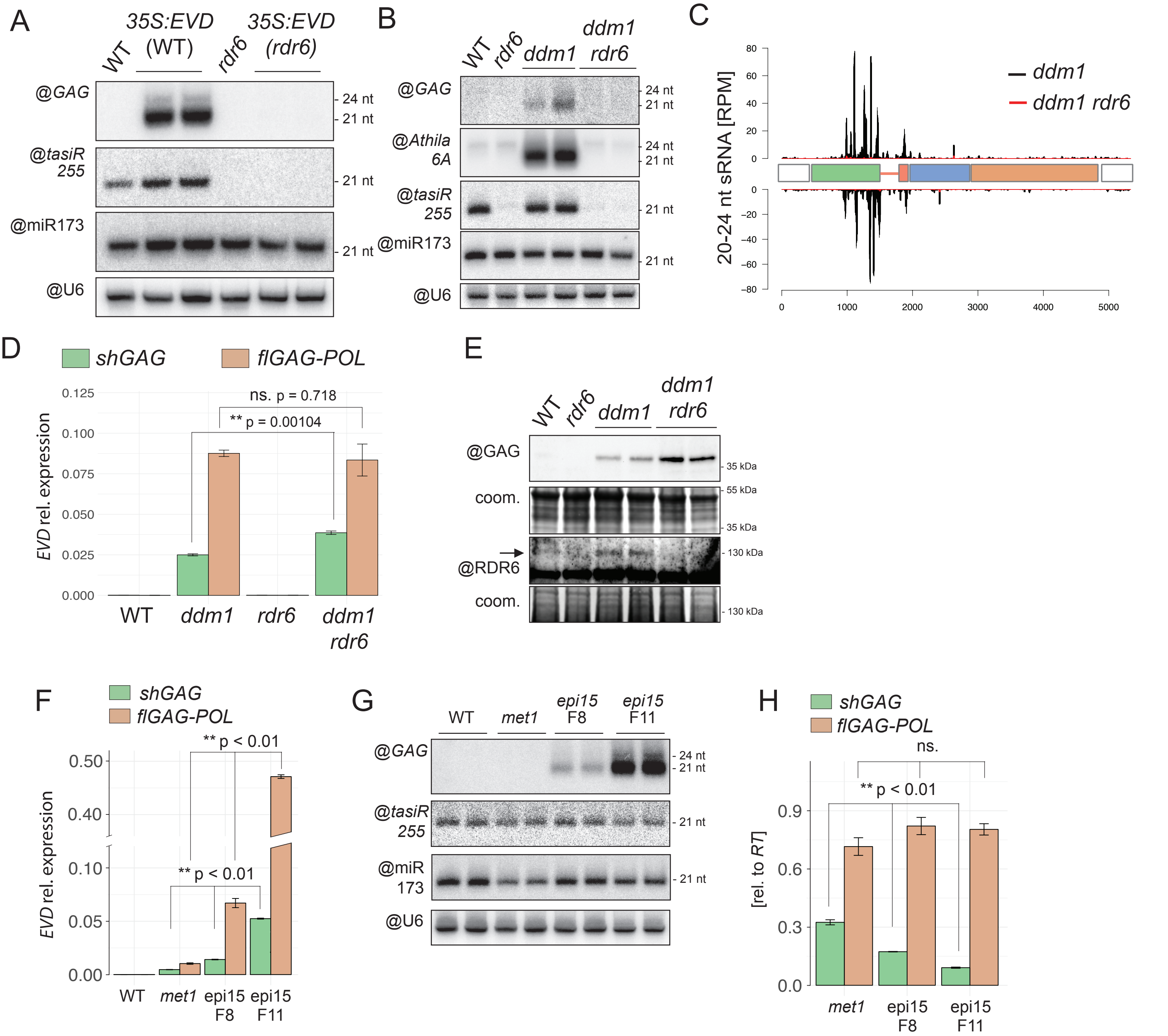


**Appendix Figure S1. The EVD spliced and prematurely terminated shGAG mRNA is a trigger and target of RDR6-dependent silencing. (A)** Low molecular weight RNA analysis from *35S:EVDwt* in the WT or *rdr6* background with probes against *GAG* or tasiR255 as a control for the *rdr6* mutation or miR173 and U6 as loading controls. **(B-E)** Characterization of endogenous *EVD* in *ddm1* or *ddm1 rdr6* backgrounds. (B) Low molecular weight RNA analysis. *Athila 6A*, tasiR255, miR173 and U6 are used as controls. (C) 20-24 nt siRNA profile on the *EVD* locus. RPM: Reads per million. (D) Relative expression of *shGAG* versus *flGAGPOL* mRNAs. (E) Western analysis of GAG and RDR6. Coomassie (coom.) blue staining provides a loading control. **(F-H)** Endogenous *EVD* expression and *GAG* siRNA accumulation caused by increased copy number in WT, *met1* or *met1*-derived epiRIL *epi15* from generation F8 to F11. (F) qPCR analysis of the two mRNA isoforms relative to *ACT2*. (G) Low molecular weight RNA analysis, with controls as in (B). **(H)** qPCR analysis of the spliced and unspliced transcripts levels relative to *EVD RT* expression, reflecting the ratios to the full-length isoform. In all panels: qPCR was performed on three biological replicates and normalized to *ACT2* unless indicated. Error bars represent the standard error. (**) = p-value < 0.01 (two-sided t-test against corresponding controls).

# Appendix figure S2

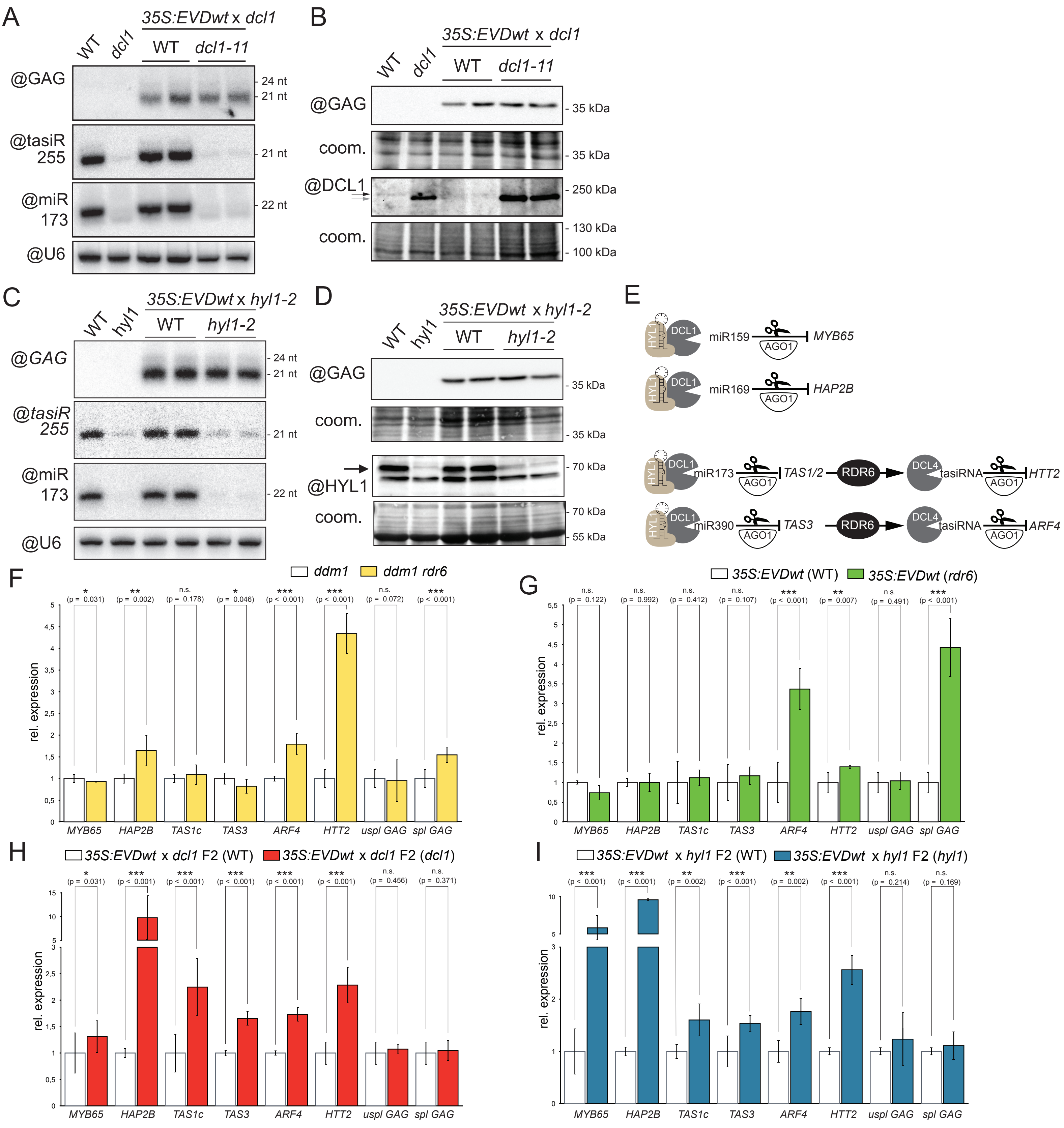


**Appendix Figure S2. miRNA-independent silencing of *EVD* in *ddm1* and *35S:EVDwt* overexpression lines. (A-B)** Analysis of *35S:EVDwt* in the *dcl1-11* versus WT background. (A) Low molecular weight RNA analysis with probes against *GAG* or miR173, tasiR255 and U6 as controls. (B) Western analysis of GAG and DCL1 . Note that WT DCL1 (black arrow) is slightly larger than the mutated dcl1-11 protein (gray arrow). dcl1-11 is also upregulated due to the loss of the miR162-mediated negative feedback loop controlling *DCL1* levels. The coomassie (coom.) blue-stained membrane is shown as a loading control. **(C-D)** Analysis of *35S:EVD* in *hyl1-2*. (C) Low molecular weight RNA analysis of *GAG* as well as miR173, tasiR255 and U6, used as controls. (Continues on next page)

(D) Western analysis of GAG and HYL1. The coomassie (coom.) blue-stained membrane provides a loading control. **(E)** Succinct schemes for DCL1- and HYL1-dependent regulation of miRNA targets *MYB65* and *HAP2B* (top) and for miRNA-RDR6-dependent initiation of tasiRNA biogenesis and regulation of targets *HTT2* and *ARF4* (bottom). **(F - I)** Relative expression levels of multiple miRNA targets, tasiRNA precursors, tasiRNA targets and EVD transcript in *35S:EVDwt* in the indicated genetic backgrounds as used in Fig.1, Fig.2, Supp.Fig.1 and Supp.Fig.2. In all panels: qPCR was performed in three biological replicates. Error bars represent the standard error. (ns.) = non-significant, (*) = p-value < 0.05 , (**) = p-value < 0.01, (***) = p-value < 0.001, (two-sided t-test against corresponding controls).

# Appendix figure S3

**Defective shGAG poly(A)-tailing does not underlie *EVD* siRNA production.**

Transgene mRNAs lacking a poly-adenylation (poly(A)) tail as a result of aberrant transcription stimulate RDR6 activity in vivo. To test if potential poly(A) defects could explain RDR6 affinity for the spliced shGAG mRNA, total polyadenylated (poly(A)+) versus non-polyadenylated (poly(A)-) RNA was fractionated from *35S:EVDwt* tissues in eitherthe WT or *rdr6* background. This was confirmed in*ddm1* and *ddm1 rdr6* non-transgenic plants in which EVD is epigenetically reactivated (Fig.1, S1). In both settings, *shGAG* was near-exclusively poly(A)+ (Fig.S3A-E). Accordingly, the increased *shGAG* mRNA levels in rdr6 were contributed by the global poly(A)+, not poly(A)-, fraction (Fig.S3B,E). Therefore, aberrant transcription leading to poly(A)-tail-deficiency is unlikely to stimulate RDR6 recruitment specifically on *shGAG*.

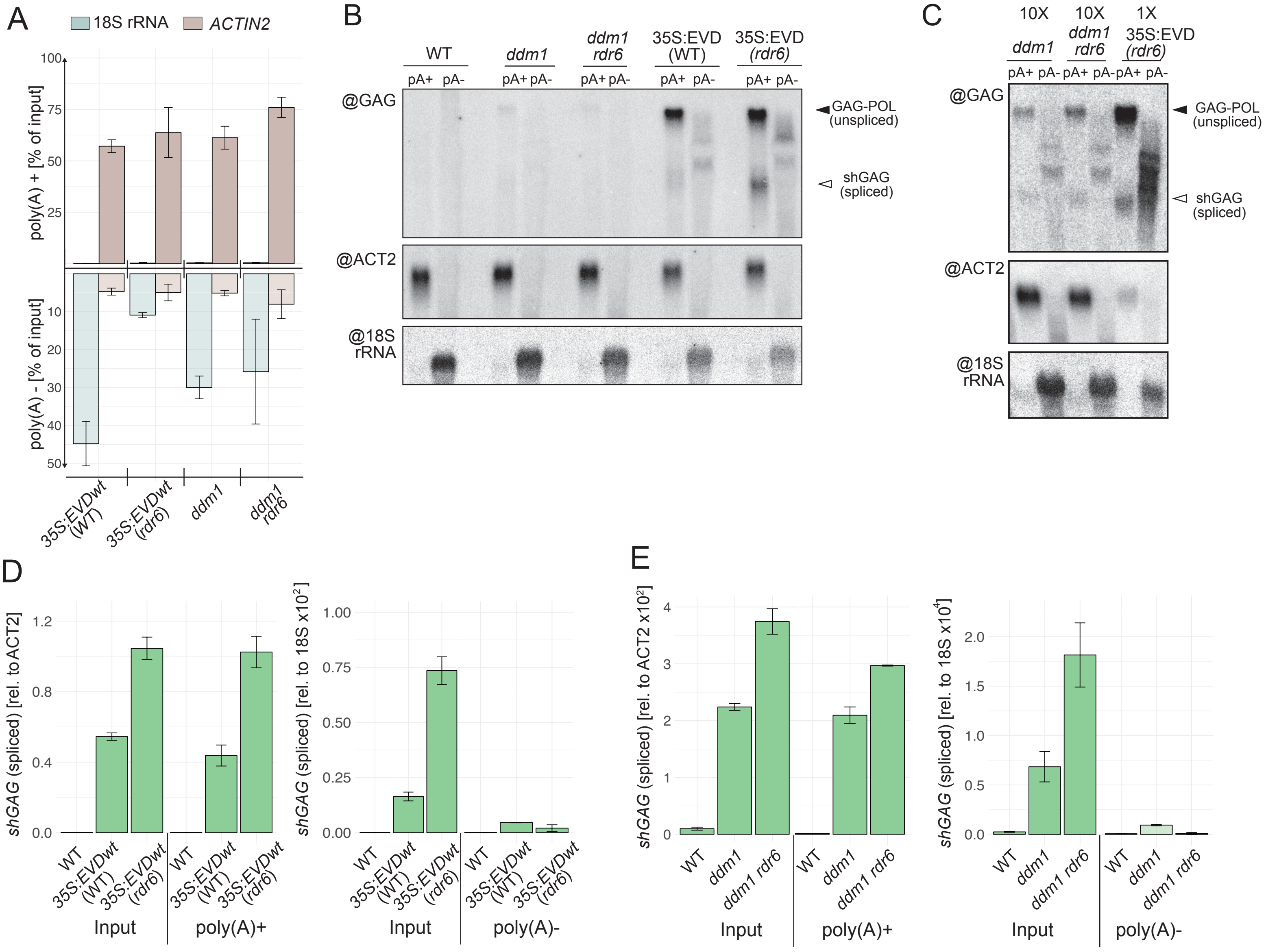


**Appendix Figure S3. Effect of rdr6 on endogenous and transgenic polyadenylated versus non-polyadenylated EVD RNA levels. (A)** qPCR assessment of poly(A)- versus poly(A)+ RNA fractionation after two subsequent oligo(dT) purifications on two biological replicates for each sample. Quantification involves 18S rRNA (poly(A)-) and *ACT2* (poly(A)+) controls relative to input (total RNA). Note that two poly(A) separation steps are not expected to yield a 100% recovery. A third biological replicate was investigated through Northern blot (B, C). **(B)** RNA blot of poly(A)+ and poly(A)- RNA fractions probed against *EVD GAG*. *ACT2* and 18S rRNA serve as poly(A)+ and poly(A)- controls respectively. **(C)** Same

(*continues on next page*)

as in B but loading 10X more RNA for ddm1 samples relative to *35S:EVD* (*rdr6*) to compensate for the difference in EVD expression levels. **(D)** *shGAG* mRNA quantification in input, poly(A) + and poly(A)- fractions relative to *ACT2* (poly(A)+) and 18S rRNA (poly(A)-) in *35S:EVDwt* in the WT or *rdr6* background. **(E)** Same as in (B) but in the *ddm1* versus *ddm1 rdr6* background. qPCR was performed in biological duplicates and error bars represent the standard error.

# Appendix figure S4

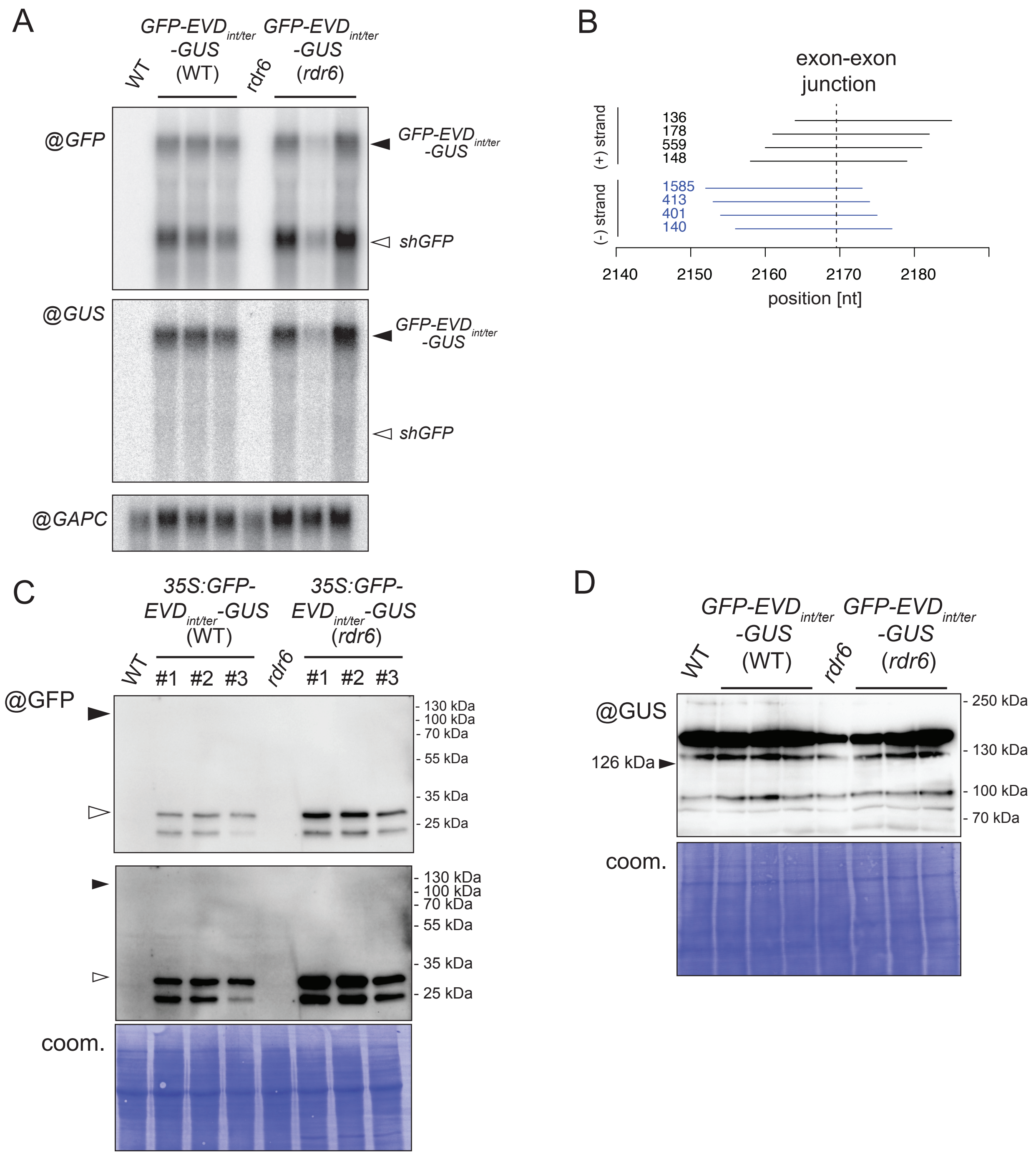


**Appendix Figure S4. Molecular characterization of 35S:GFP-EVDint/ter-GUS expressed in the WT or rdr6 background. (A)** High molecular weight RNA analysis probing for GFP and GUS regions in three independent transgenic lines in either WT or rdr6 alongside with non-transformed controls. GAPC probing is used as a loading control. **(B)** sRNA reads mapping at the splice junction of the EVD intron on the positive (+) and negative (-) strand alongside their abundance in the WT background. No reads covering the intron junction were found in rdr6. **(C-D)** western analysis of GFP (C) and GUS (D). GFP-only protein (white arrow; ~30 kDa), but not the GFP-GUS fusion protein (back arrow; 126kDa) was detected. Coomassie staining (coom.) of the membrane serves as loading control.

# Appendix figure S5

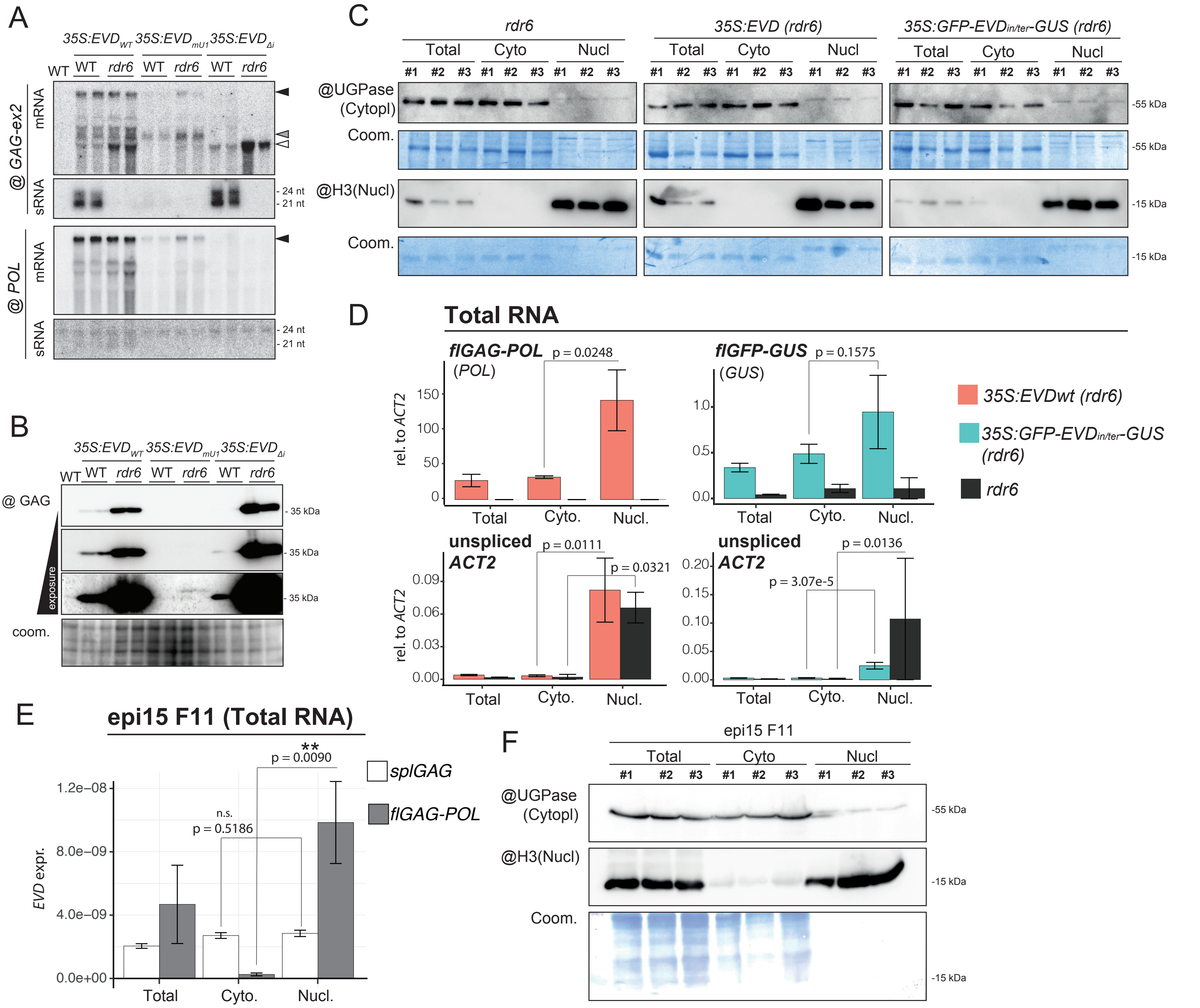


**Appendix Figure S5. Impact of splicing and premature termination on EVD silencing and mRNA localization.** **(A)** High and low molecular RNA analysis of the *shGAG* (GAG-ex2 probe) or *l GAG-POL* (POL probe) transcripts. The mRNA isoforms are labelled with arrows on the side or with an asterisk and correspond to the transcripts depicted in Fig.3A-C. Loading controls are displayed in Fig.3E. **(B)** Same western analysis of the GAG protein as presented in Fig.3F, but with three increasingly higher exposure times and Coomassie loading control (coom.). **(C)** Quality assessment of nucleo-cytosolic fractionations from *rdr6*, *35S:EVD(rdr6)* and *35S:GFP-EVDint/ter-GUS (rdr6)* by western analysis. UGPase and HISTONE 3 (H3) were used as cytoplasmic and nuclear protein markers, respectively, in protein extracted from total, cytoplasmic (Cyto) or nuclear (Nucl) fractions from three independent experiments. Coomassie staining (coom.) of the membranes serves as loading control. **(D)** Nucleo-cytosolic distribution of unspliced *ACT2*, *35S:EVD* and *35S:GFP-EVDint/ter-GUS* full-length RNA in the rdr6 background relative to that of spliced *ACT2* by qPCR. RNA extracted from Total, nuclear and cytoplasmic fractions was reverse transcribed with oligo(dT) to account only for polyadenylated RNA. **(E)** Nucleo-cytosolic distribution of endogenous *EVD* RNA isoforms in the *epi15* F11 analyzed by qPCR from total, cytoplasmic and nuclear fractions. **(F)** Quality assessment of nucleo-cytosolic fractionations, as in C, from epi15 F11 in the three biological replicates used in E. In D and E qPCR was performed on n=3 biological replicates; bars: standard error. (*) = p-value < 0.05; (**) = p-value < 0.01; n.s = non significant (p-value > 0.05) (two-sided t-test between indicated samples).

# Appendix figure S6

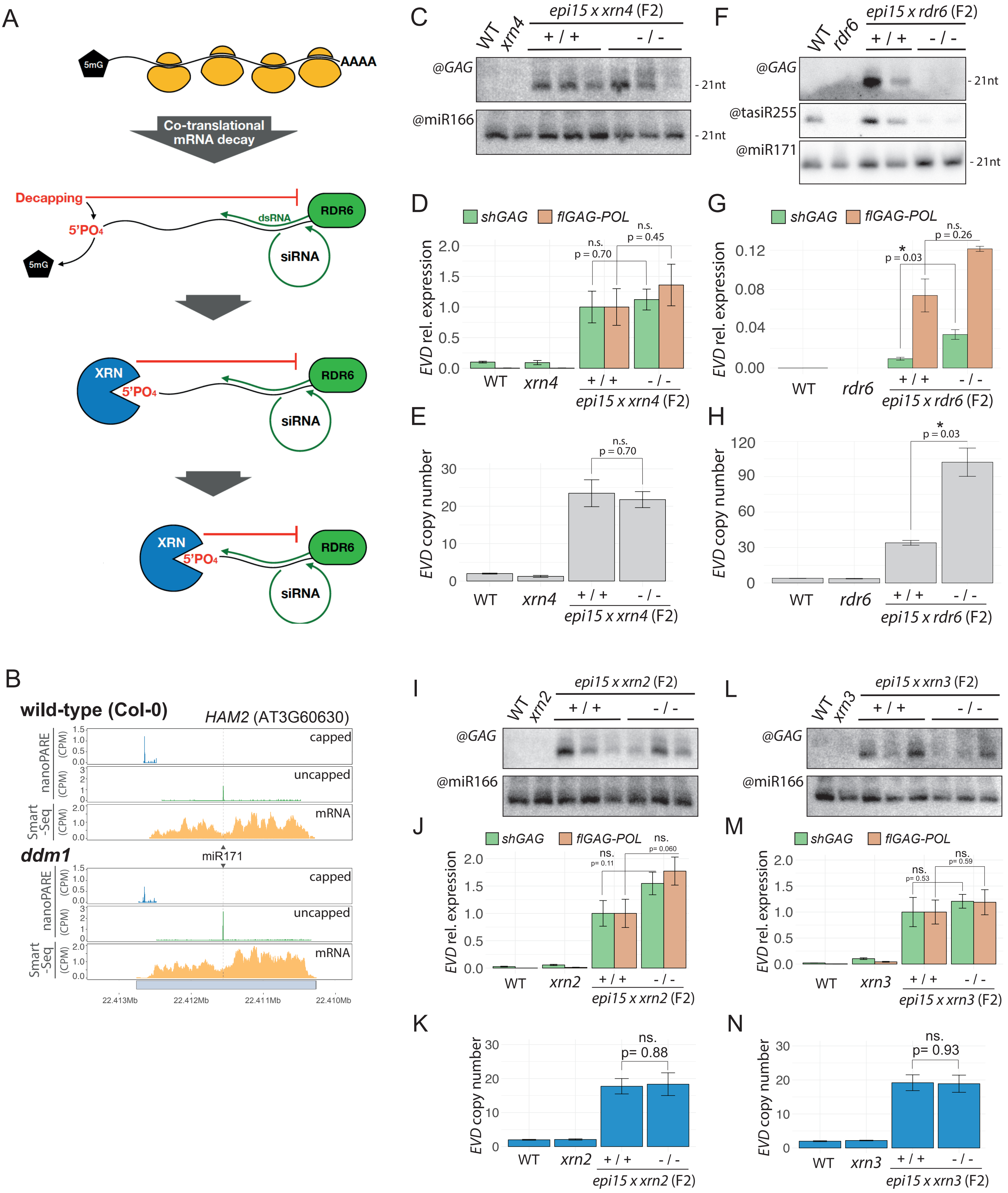


(See legend on next page)

**Appendix Figure S6. XRN4-mediated co-translational mRNA decay does not influence siRNA production from shGAG.**

**(A)** During co-translational decay, mRNA turnover is initiated by decapping of actively translated mRNAs. This exposes 5' monophosphate (5'PO4) groups required for the 5'->3' exonucleolytic activity of XRNs. As evidenced by the RDR6-dependent production of siRNAs from dozens of endogenous loci in decapping and xrn4 mutants of Arabidopsis, plant mRNAs engaged in co-translational decay can become substrates for siRNA biogenesis if they are not degraded by, or their levels saturate, this process. While depicted here, for simplicity, as a co-translational event, RDR6 action onto such RNA likely occurs in specialized "siRNA bodies" adjacent to P-bodies **(B)** Capped and uncapped 5'ends mapping with nanoPARE on the miR171 target *HAM2* in WT and *ddm1*, along with the corresponding Smart-seq coverage (mRNA). Capped and uncapped defined respectively as reads overlapping, or not, with capped 5' clusters (reads containing 5' untemplated G). **(C-E)** EVD genomic proliferation in homozygous xrn4 mutant and WT backgrounds in F2 plants from a cross between xrn4 and epi15 exhibiting active EVD mobilization. (C) sRNA blot analysis using an anti-GAG probe. (D) Relative expression levels of shGAG and flGAGPOL normalized to ACT2 and to AT4G26410 levels. (E) EVD genomic copy number quantification by qPCR. qPCR analysis was performed on three biological replicates for controls and the three independent WT and mutant F2 lines displayed in B. **(F-H)** Same as (C-E) but with the rdr6 versus WT epi15 backgrounds. **(I-K)** Impact of loss-of-XRN2 function on active *EVD* assessed in homozygous *xrn2* and WT backgrounds in F2 plants from a cross between *xrn2* and *epi15* plants undergoing active EVD mobilization. (I) Low molecular weight RNA analysis, with miR166 as a loading control. (J) Relative expression levels of *shGAG* and *flGAGPOL*. (K) EVD genomic copy number determined by qPCR assay on genomic DNA. **(L-N)** Same as (I-K) but with the *xrn3* mutation.
In all panels, qPCR was performed on three biological replicates for controls and three independent WT and mutant F2 lines. Error bars display standard errors. (ns.) = non-significant, (*) = p-value < 0.05, (**) = p-value < 0.01 (two-sided t-test between indicated samples).

# Appendix figure S7

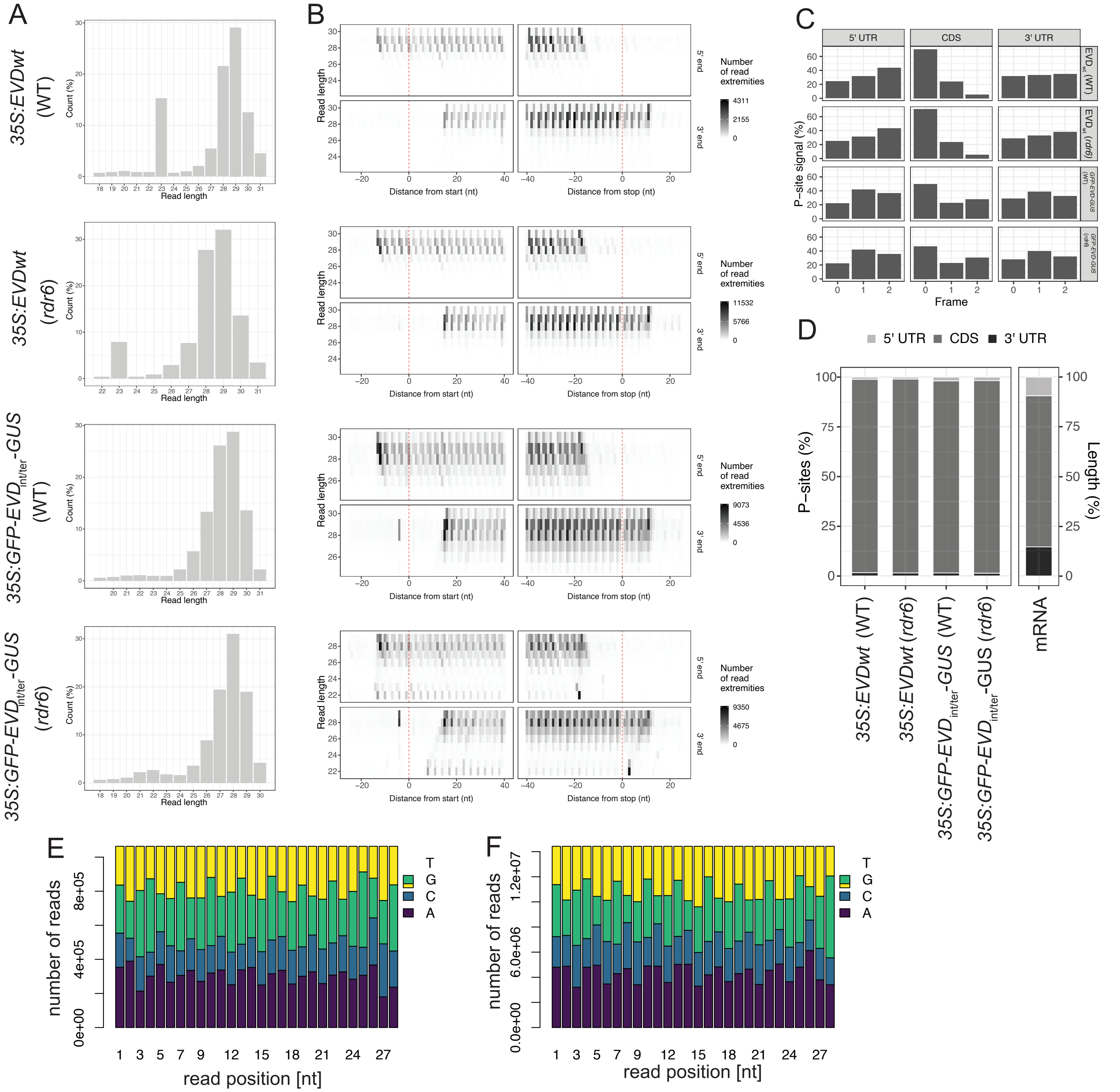


**Appendix Figure S7. Quality control of the ribosomal footprinting libraries in the indicated genetic backgrounds.** **(A)** Read-length profiles of reads mapping to transcripts of protein coding genes in each of the indicated library. **(B)** Footprinting periodicity and offset across read lengths of 24 to 30 nt for each library. The translational start (left) and stop (right) sites are indicated with red vertical lines. Reads densities on the 5′ end (top) and the 3′ end (bottom) are displayed. **(C)** Triplet periodicity was captured inside coding sequences (CDS) but no in untranslated regions (UTRs) upon assigning reads counts to the three positions within each codon. **(D)** Significant enrichment of reads coverage from CDS as opposed to both 5′ and 3′ UTRs compared with the average length of those elements within Arabidopsis transcripts. **(E)** Nucleotide composition at each position of 28 nt long reads from the 35S:EVDwt library (WT background) used in this work. **(F)** Same as in (E) but with a library prepared according to the original RIBO-seq protocol described by Ingolia et al. 2009, presented here for comparison.

# Appendix figure S8

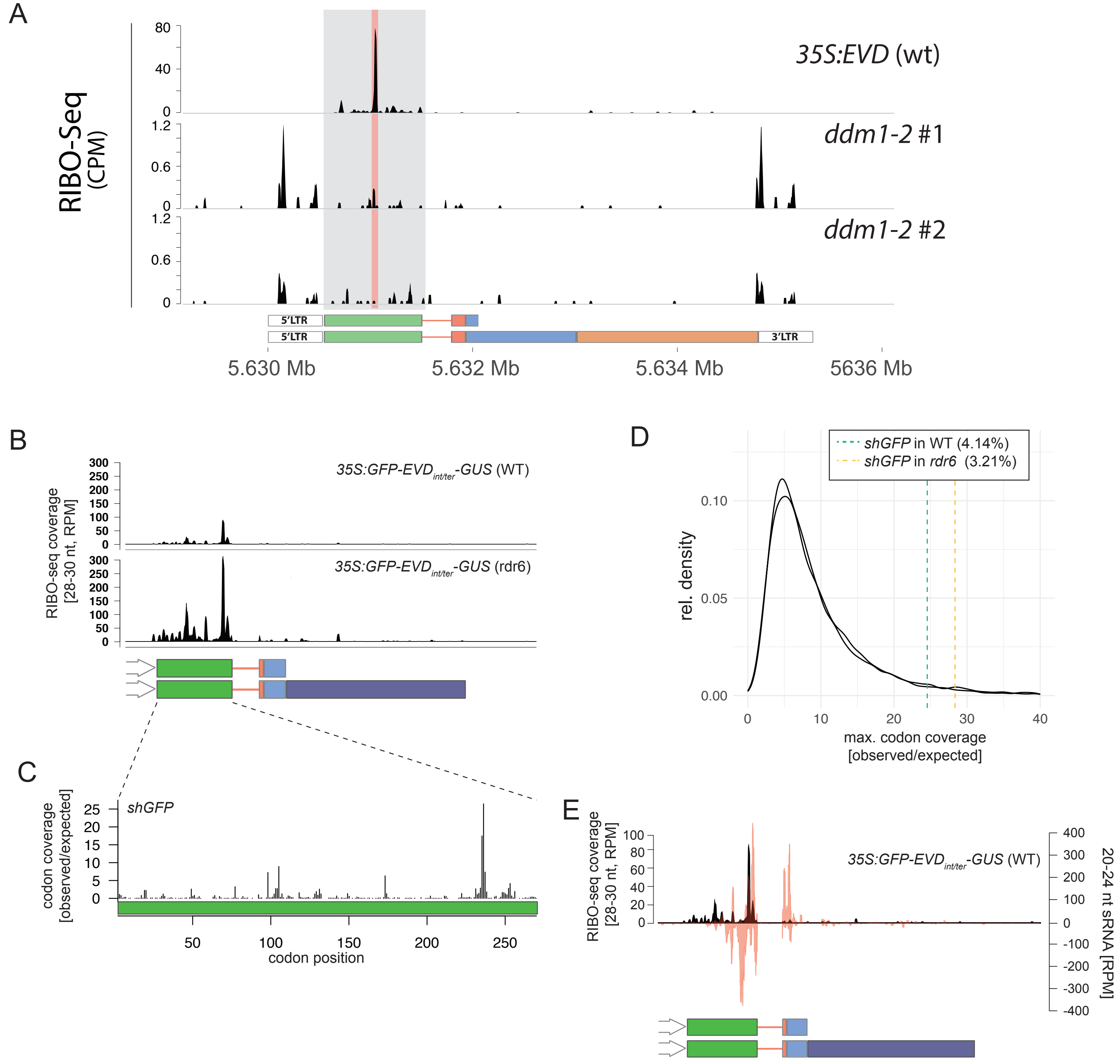


**Appendix Figure S8. Ribosome footprints on 35S:GFP-EVDint/ter-GUS. (A)** *EVD* RIBO-seq coverage profiles on the *35S:EVD* in WT and two *ddm1-2* libraries. The *GAG* coding region is highlighted in grey, and the conserved stalling site in both *35S:EVD* in WT and *rdr6* (Fig.7A) is highlighted in red. CPM: Counts per million. **(B)** RIBO-seq coverage profiles on the 35S:GFP-EVDint/ter-GUS in WT and rdr6. RPM: Reads per million. **(C)** Ribosomal footprints compiled to display codon occupancy at the P-sites over the GFP sequence. Observed coverage at each codon position was divided by the expected mean coverage along the entire GFP coding sequence. **(D)** Maximal individual codon coverage over the expected coverage for all translated transcripts of Arabidopsis. Vertical lines indicate the strength of stalling sites on shGFP in the WT or rdr6 background. Percentages specify the proportion of transcripts with more pronounced stalling events than the shGFP ones. **(E)** Overlay between 35S:GFP-EVDint/ter-GUS siRNAs in WT and RIBO-seq profiles in the *rdr6* background.

# Appendix figure S9

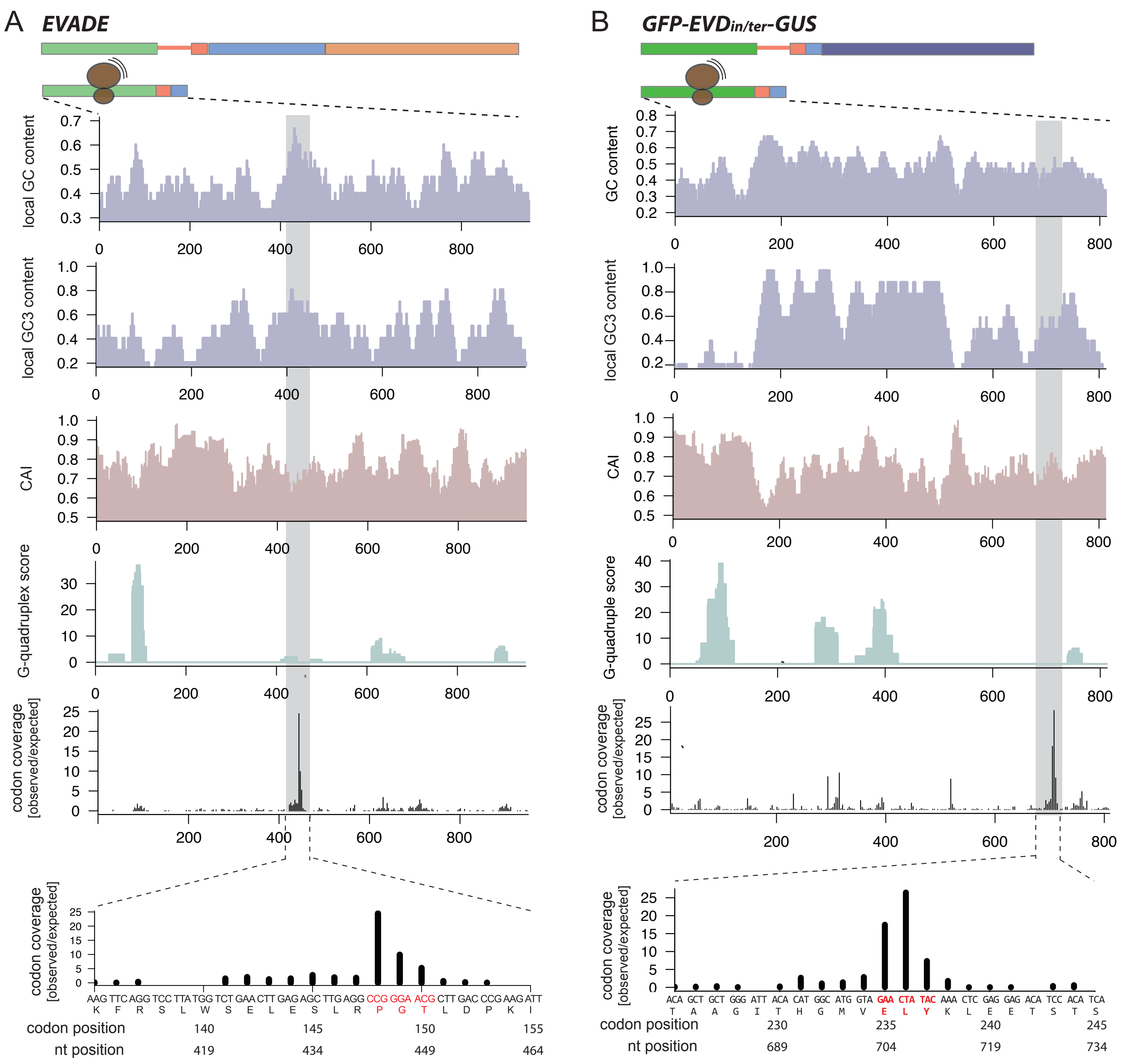


**Appendix Figure S9. Sequence properties with the potential to impact translation of shGAG (A) and shGFP (B) transcripts.** For local GC content, GC content at codon position three and codon adaptivity index, sequences were analysed as 30 nucleotide long sliding windows shifting by three nucleotide steps representing one codon shifts. G-quad-ruplex scores were predicted as previously reported with pqsfinder. Codon coverage represents the ribosomal footprints compiled to display codon occupancy at the P-sites over the *shGAG* and *shGFP* sequence. Observed coverage at each codon position was divided by the expected mean coverage along the entire GFP coding sequence.

# Appendix figure S10

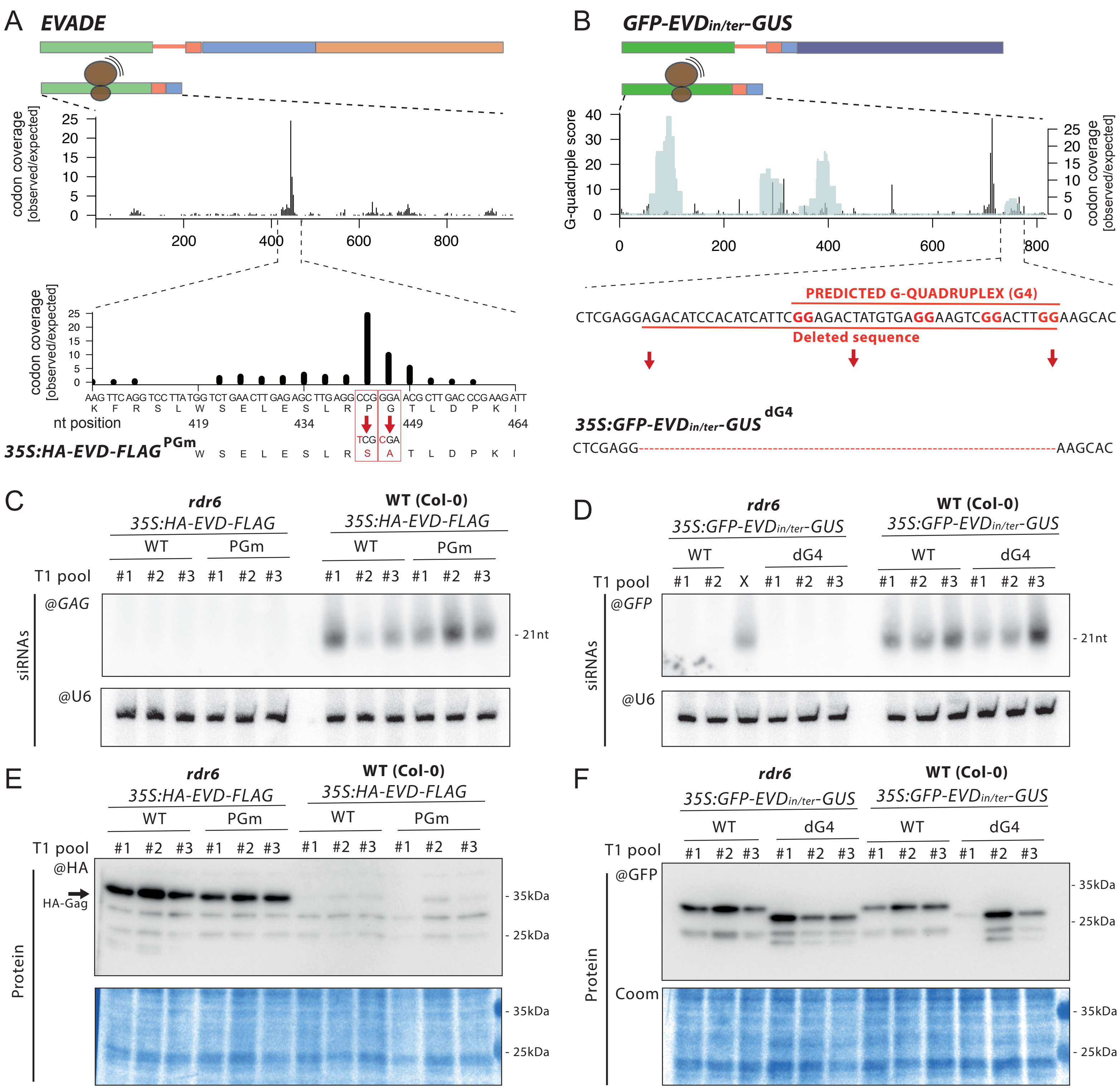


**Appendix Figure S10. Neither aminoacid identity nor G-quadruplex impact siRNA production or translation of shGAG (A) and shGFP (B) respectively.** **(A)** To ivestigate if the consecutive proline (P) and glycine (G) aminoacids were responsible for the ribosome stalling event leading to the initiation of siRNAs in *EVD shGAG* two single nucleotide mutations were introduce in a *35S:HA-EVD-FLAG* construct in order to generate EVD P and G mutant (PGm) carrying both P148S and G149A substitutions. **(B)** In parallel, the predicted G-quadruplex (G4) inmediately downstream of the *shGFP* stalling site (see Suppl. Fig. 9B) was removed from the *35S:GFP-EVDin/ter-GUS* construct (dG4). Three independet pools of trans-genic T1 plants were used to asess the impact of introduced mutations in siRNA (C, D) and protein levels (E, F). **(C)** sRNA blot of *35S:HA-EVD-FLAG*$^{WT}$ and *35S:HA-EVD-FLAG*$^{PGm}$ in WT and *rdr6* backgrounds probed against GAG-derived siRNAs.
(Continues on next page)

**(D)** sRNA blot of *35S:GFP-EVDin/ter-GUS*$^{WT}$ and *35S:GFP-EVDin/ter-GUS*$^{dG4}$ in WT and *rdr6* backgrounds probed against GFP-derived siRNAs. X idicates a missloaded sample from WT Arabidopsis background. In C and D U6 snoRNA serves as loading control. **(E)** anti-HA Western blot of *35S:HA-EVD-FLAG*$^{WT}$ and *35S:HA-EVD-FLAG*$^{PGm}$ in WT and *rdr6* backgrounds to detect *HA-Gag*. **(F)** anti-GFP Western blot of of *35S:GFP-EVDin/ter-GUS*$^{WT}$ and *35S:GFP-EVDin/ter-GUS*$^{dG4}$ in WT and *rdr6* backgrounds. The deletion in dG4 lines results in shorter *shGFP* translation products. In E and F, coomassie staining of the membrane serves as loading control.

# Appendix figure S11

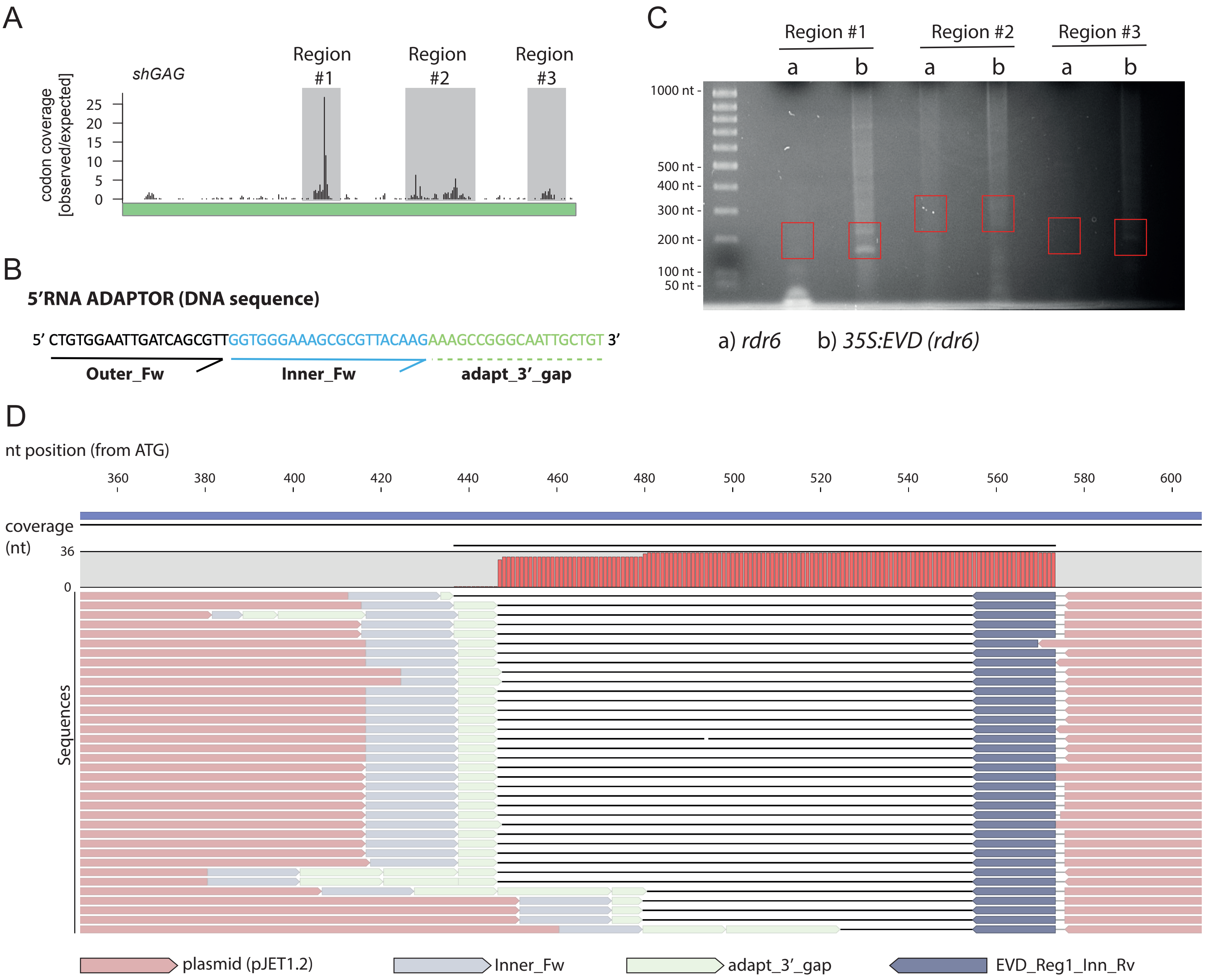


**Appendix Figure S11. Cloning and Mapping of *EVD* 5'OH-ends.**
**(A)** Ribosome footprints over *EVD shGAG* transcript. The three regions inspected for atypical 5'OH ends are higlighted in grey. **(B)** Sequence of the RNA adaptor used to ligate to 5'OH ends with *RbtC*. Binding sites for outer and inner primers for nested PCR are indicated. **(C)** Ethidium Bromide staining of amplification products from second nested PCR in *rdr6* and *35S:EVD (rdr6)* resolved on an agarose gel. Independently of the presence/absence of bands, for all PCRs, gel was excised (red squares) for DNA extraction and cloning at the expected size for any potential amplicon within the regions highlighted in A. **(D)** Mapping, alignment and annotation of positive clones/colonies. To discard potential PCR artifacts, only clones displaying at least 1/4 of adaptor sequence between adaptor Inner_Fw primer and *EVD* sequence (adapt_3' _gap, B) were taken for the analysis. Nucleotide-resolution mapping of the 5'ends is shown in Fig.6F.

# Appendix figure S12

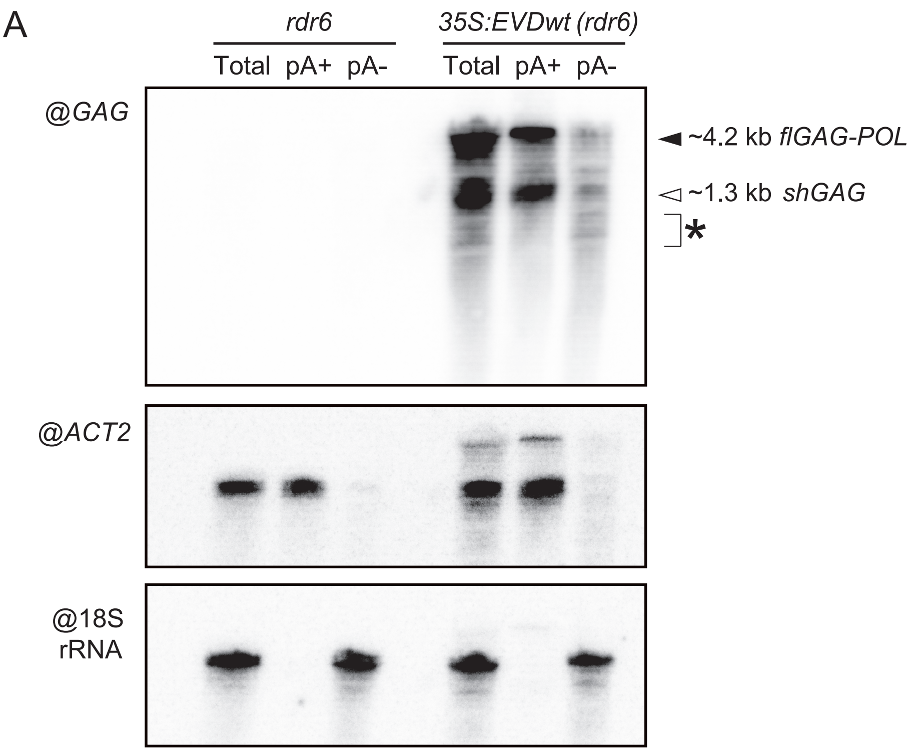


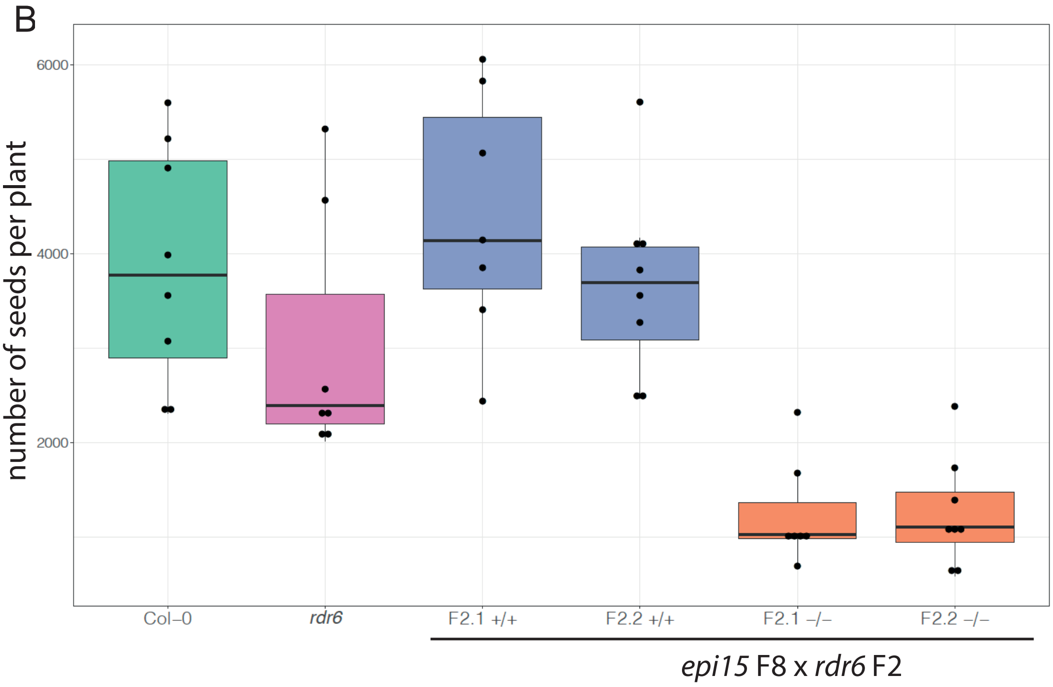


**PAIRWISE COMPARISON P-VALUES:**

| | Col-0 | F2.1 (-/-) | F2.1 (+/+) | F2.2 (-/-) | F2.2 (+/+) |
|---|---|---|---|---|---|
| F2.1 (-/-) | ** 0.0064 | —— | —— | —— | —— |
| F2.1 (+/+) | n.s. 1.0000 | ** 0.0064 | —— | —— | —— |
| F2.2 (-/-) | ** 0.0044 | n.s. 1.0000 | ** 0.0044 | —— | —— |
| F2.2 (+/+) | n.s. 1.0000 | ** 0.0044 | n.s. 1.0000 | ** 0.0023 | —— |
| *rdr6* | n.s. 1.0000 | * 0.0326 | n.s. 0.5099 | * 0.0196 | n.s. 1.0000 |

**Appendix Figure S12. (A)** Separation of poly(A)+ and poly(A)- RNA from *35S:EVDwt* in rdr6 on a 4% PAGE gel hybridized with a probe against *GAG* to detect both *shGAG* and *flGAG-POL* mRNA isoforms. Putative stalling-linked RNA cleavage fragments are indicated with an asterisk. The membrane was subsequently probed against *ACT2* and 18S rRNA as controls for the quality of the fractionation. **(B)** Impact of *EVD* over-proliferation in *epi15* with the *rdr6* as opposed to WT background. The total amount of seeds was counted in 7 to 8 individual plants from controls and homozygous WT or mutants from two individual F2 populations derived from crosses between *epi15* (carrying active EVD) and *rdr6* plants, as in Fig.6. Data points represent the median of two consecutive seed counts measurememnts for each individual plant. Table shows p-values (Holm-adjusted method) for pairwise comparison using Wilcoxon rank sum test (n.s: non significant*: p-value <0.05, **: p-value <0.01).

# Appendix Discussion:

### Possible cause(s) of ribosome stalling

Our findings raise the key question of what molecular signal(s) might cause the unusually intense stalling events observed in the s*hGAG* and *shGFP* ORFs that possibly activate TdS. While studies of ribosome stalling in plants are very scarce, suboptimal codon usage has been widely considered as one of its possible causes (Rocha, 2004; Quax *et al*, 2015). Indeed, extensive stretches of rare codons trigger ribosome stalling and RNA degradation when artificially engineered in reporter mRNAs (Li *et al*, 2006; Yang *et al*, 2019; Park & Subramaniam, 2019) . However, it has been noted that this rarely applies to endogenous mRNAs, where non-optimal codons are common and play roles in translation regulation (Hanson & Coller, 2018; Carneiro *et al*, 2019). Accordingly, recent in-depth analyses in animals and fungi indicate that codon usage *per se* is not predictive of stalling on endogenous mRNAs, and that other factors might modulate ribosome dwell times (Gardin *et al*, 2014; Dana & Tuller, 2014; Rodnina, 2016; Zhang *et al*, 2015; Wu *et al*, 2019). *shGAG* and *shGFP* are devoid of rare codons among other inspected features, suggesting that no universal sequence/structure feature might underpin the stalling events correlating with TdS. Rather, mRNA-intrinsic and variable features, or combinations thereof, might be involved. mRNA-extrinsic features could also contribute to intense and discrete stalling in *shGAG* via *trans*-interactions involving specific RNA sequence-motifs and RNA-binding proteins (RBP), a circumstance that can hinder ribosome progression (Babitzke *et al*, 2009; Iwakawa & Tomari, 2015; Zhang *et al*, 2015).

The action of RNA-induced silencing complexes (RISCs) formed between AGOs and miRNAs (miRISCs) could illustrate how RBPs may, under some circumstances, elicit TdS, for instance during the intricate biogenesis of *TAS3* trans-acting (ta)siRNAs (Hou *et al*, 2016; Xia *et al*, 2017). Observed in virtually all land plants, it involves AGO7-mediated recognition of a highly conserved, non-cleavable miR390 target site located within a miniature ORF invariably borne by *TAS3*-precusor transcripts. By increasing the AGO7 dwell-time on the translating mini-ORF, the non-sliceable miR390 site might cause elongating ribosomes to collide into

AGO7 (Hou *et al*, 2016; Iwakawa *et al*, 2021). A TdS-like process likely follows because the 5'-end of the ensuing RDR6-dependent tasiRNA pattern coincides with the AGO7:miR390 target site therefore suggesting the co-option of TdS into a conserved gene regulation machinery. Strikingly, in *TAS3*, the siRNA pattern's 3'-end matches a second evolutionary conserved, yet this time cleavable, miR390 target site downstream of the mini-ORF (Rajeswaran & Pooggin, 2012; Felippes *et al*, 2017; Xia *et al*, 2017). Production of other tasiRNAs and tasiRNA-related "phasiRNAs" is also typically initiated by longer-than-normal miRNAs (Cuperus *et al*, 2010; Liu *et al*, 2020) possibly increasing AGO1's dwell time concurrently to translation.

Recently, the dsRNA-binding protein SGS3 was shown to hinder ribosome elongation via stalling due to its interaction with AGO7 or with the protruding end of 22-nt miRNAs loaded into AGO1. In both *TAS1* and *TAS3,* ribosome stalling upstream of miRNA target sites is abolished in *sgs3* mutants and tasiRNA production reduced, suggesting that the ribosome stalling event induced by SGS3-RISC enhances siRNA production (Iwakawa *et al*, 2021). How RDR6 is recruited, or stalled RNAs brought into RDR6-rich siRNA-bodies, remains to be determined but perhaps 5'OH fragments similar to those described here for *EVD* underpin this process. A similar role for a miRISC mediating ribosome stalling on *EVD* is excluded based on the lack-of-effect of *dcl1* and *hyl1* on *EVD*-derived siRNA production (Fig.1F-G, S2) and detectable miRNA-mediated cleavage RNA 5'ends (Fig.6A). It remains formally possible, however, that RDR6-independent siRNAs or RNA breakdown products akin to "primal" sRNAs in *S. pombe* (Halic & Moazed, 2010)*,* antisense to *EVD,* might spuriously load into AGO1/2/7 to promote stalling. Although we did not formally introduce *EVD* in *ago1/2/7* mutants to investigate this possibility, we found that background levels of sRNAs produced in *rdr6* from both endogenous and transgenic *EVD* show no obvious enrichment downstream of the main ribosome stalling site (Fig.1B, S1C). A closer inspection revealed that, over the coding region, background 21-to-24-nt siRNAs are equally abundant along GAG and POL regions, and are mostly in the sense, as opposed to antisense, orientation (Fig.S13). Their low abundance, sense orientation and widespread distribution most likely reflects that they are inert RNA degradation byproducts from both *shGAG* and *fl-Gag-POL* transcripts, making it unlikely, therefore, that these species might initiate stalling upon their loading in one/several AGOs.

## Appendix References:


Babitzke P, Baker CS & Romeo T (2009) Regulation of translation initiation by RNA binding proteins. *Annu Rev Microbiol* 63: 27–44

Carneiro RL, Requião RD, Rossetto S, Domitrovic T & Palhano FL (2019) Codon stabilization coefficient as a metric to gain insights into mRNA stability and codon bias and their relationships with translation. *Nucleic Acids Research* 47: 2216–2228

Cuperus JT, Carbonell A, Fahlgren N, Garcia-Ruiz H, Burke RT, Takeda A, Sullivan CM, Gilbert SD, Montgomery TA & Carrington JC (2010) Unique functionality of 22-nt miRNAs in triggering RDR6-dependent siRNA biogenesis from target transcripts in Arabidopsis. *Nat Struct Mol Biol* 17: 997–1003

Dana A & Tuller T (2014) The effect of tRNA levels on decoding times of mRNA codons. *Nucleic Acids Research* 42: 9171–9181

Felippes FF de, Marchais A, Sarazin A, Oberlin S & Voinnet O (2017) A single miR390 targeting event is sufficient for triggering TAS3-tasiRNA biogenesis in Arabidopsis. *Nucleic Acids Research* 45: 5539–5554

Gardin J, Yeasmin R, Yurovsky A, Cai Y, Skiena S & Futcher B (2014) Measurement of average decoding rates of the 61 sense codons in vivo. *Elife* 3

Halic M & Moazed D (2010) Dicer-Independent Primal RNAs Trigger RNAi and Heterochromatin Formation. *Cell* 140: 504–516

Hanson G & Coller J (2018) Codon optimality, bias and usage in translation and mRNA decay. *Nature Reviews Molecular Cell Biology* 19: 20–30

Hou CY, Lee WC, Chou HC, Chen AP, Chou SJ & Chen H-M (2016) Global Analysis of Truncated RNA Ends Reveals New Insights into Ribosome Stalling in Plants. *THE PLANT CELL ONLINE* 28: 2398–2416

Iwakawa H, Lam AYW, Mine A, Fujita T, Kiyokawa K, Yoshikawa M, Takeda A, Iwasaki S & Tomari Y (2021) Ribosome stalling caused by the Argonaute-microRNA-SGS3 complex regulates the production of secondary siRNAs in plants. *Cell Reports* 35: 109300

Iwakawa H & Tomari Y (2015) The Functions of MicroRNAs: mRNA Decay and Translational Repression. *Trends Cell Biol* 25: 651–665

Li X, Hirano R, Tagami H & Aiba H (2006) Protein tagging at rare codons is caused by tmRNA action at the 3' end of nonstop mRNA generated in response to ribosome stalling. *RNA* 12: 248–255

Liu Y, Teng C, Xia R & Meyers BC (2020) PhasiRNAs in Plants: Their Biogenesis, Genic Sources, and Roles in Stress Responses, Development, and Reproduction. *THE PLANT CELL ONLINE* 32: 3059–3080

Park H & Subramaniam AR (2019) Inverted translational control of eukaryotic gene expression by ribosome collisions. *PLoS Biology* 17: e3000396

Quax TEF, Claassens NJ, Söll D & Oost J van der (2015) Codon Bias as a Means to Fine-Tune Gene Expression. *Molecular Cell* 59: 149–161

Rajeswaran R & Pooggin MM (2012) RDR6-mediated synthesis of complementary RNA is terminated by miRNA stably bound to template RNA. *Nucleic Acids Research* 40: 594–599

Rocha EPC (2004) Codon usage bias from tRNA’s point of view: redundancy, specialization, and efficient decoding for translation optimization. *Genome Research* 14: 2279–2286

Rodnina MV (2016) The ribosome in action: Tuning of translational efficiency and protein folding. *Protein science : a publication of the Protein Society* 25: 1390–1406

Wu CC-C, Zinshteyn B, Wehner KA & Green R (2019) High-Resolution Ribosome Profiling Defines Discrete Ribosome Elongation States and Translational Regulation during Cellular Stress. *Molecular Cell* 73: 959-970.e5

Xia R, Xu J & Meyers BC (2017) The Emergence, Evolution, and Diversification of the miR390-TAS3-ARF Pathway in Land Plants. *THE PLANT CELL ONLINE* 29: 1232–1247

Yang Q, Yu C-H, Zhao F, Dang Y, Wu C, Xie P, Sachs MS & Liu Y (2019) eRF1 mediates codon usage effects on mRNA translation efficiency through premature termination at rare codons. *Nucleic Acids Research* 47: 9243–9258

Zhang X, Zhu Y, Liu X, Hong X, Xu Y, Zhu P, Shen Y, Wu H, Ji Y, Wen X, *et al* (2015) Plant biology. Suppression of endogenous gene silencing by bidirectional cytoplasmic RNA decay in Arabidopsis. *Science* 348: 120–123

# Appendix figure S13

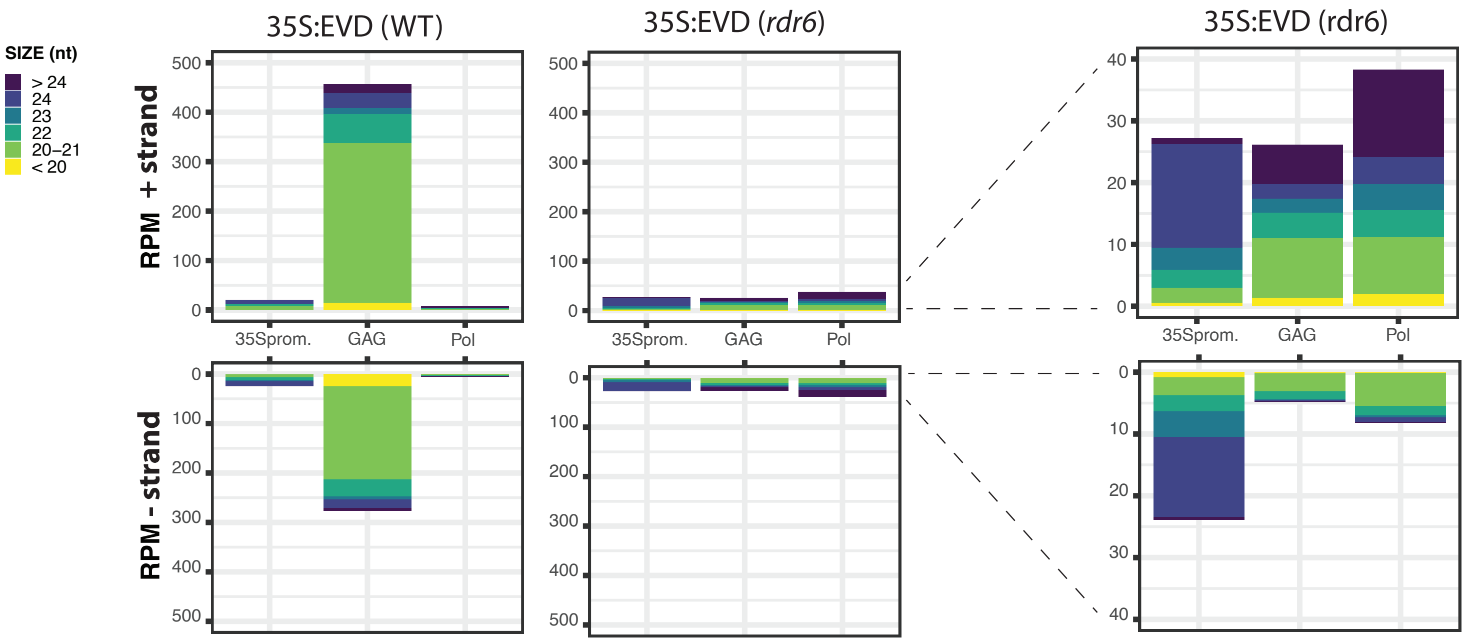


**Appendix Figure S13. Abundance and size distribution of sense and antisense *EVD* siRNAs.** Abundances of sense and antisense siRNA (RPM: reads per million reads) by size (nt) mapping to the 35S promoter (35Sprom.), GAG and Pol coding sequences in 35S:EVD transgenic lines in WT and *rdr6.*

## Appendix Table S1. Oligonucleotides used in this work.

**Genotyping primers**

| Target | Primer | Sequence 5'->3' | NOTES |
|---|---|---|---|
| ddm1-2 | ddm1 F | GCTGGAAGGGAAAGCTTAACAACCT | G-to-A mutation leads to the loss of a HaeIII restriction site: |
| (EMS mutant) | ddm1 R | acactgccatcgattctgcaaacc | wt: 80+121+218nt; ddm1: 121+298nt |
| dcl1-11 | dcl1-11 F | CGGGAATTTGTGAAGGAGGTTC | Use BIN-LB primer for mutant genotyping |
| (T-DNA insertion) | dcl1-11 R | CCTCTATCGCTCGTATTAACTC | (CGTCCGCAATGTGTTATTAAG) |
| hyl1-2 | hyl1-2 F | TTCTTGGAAATTGGATTGCAG | Use Salk T-DNA primer for mutant genotyping |
| (SALK_064863) | hyl1-2 R | AGTTCTCCCAGCGCTAATCTC | (ATTTTGCCGATTTCGGAAC) |
| xrn2-2 | xrn2 F | GCGCAAGTGGAGAAATCACT | Use Sail LB3 primer for mutant genotyping |
| (SAIL_781E02) | xrn2 R | TTTTGTCCCCATCTTCATGCC | (TAGCATCTGAATTTCATAACCAATCTCGATACAC) |
| xrn3-3 | xrn3 F | GCCTTCGATTTCAACAGGC | Use Sail LB3 primer for mutant genotyping |
| (SAIL_1172C07) | xrn3 R | GAAATCGAACACAAATCCG | (TAGCATCTGAATTTCATAACCAATCTCGATACAC) |
| xrn4-3 | xrn4 F | TCCCATGAGAGCCATGCATTC | Use Salk T-DNA primer for mutant genotyping |
| (SALK_020882) | xrn4 R | ACCATCCTGCAGGTCCAAGAA | (ATTTTGCCGATTTCGGAAC) |
| rdr6-12 | rdr6-12 F | CGTAATGAGCCTTGTTTTGG | 178 bp (-7bp for mutant allele) |
| (Fast neutron mutant) | rdr6-12 R | CGTCCTTGGGTTGGTTTCTTAG | |

**Northen blot probes**

| Target | Primer | Sequence 5'->3' |
|---|---|---|
| EVD GAG siRNA/mRNA | GAG F | TAAGTCAAGAAGACTTAGAGTTTA |
| | GAG R | ACTTTGCTCCTCATGAGTTTCTT |
| EVD "exon1" siRNA/mRNA | GAG-ex1 F | CGGCTAACAAAGGAGAAGTAGTGG |
| | GAG-ex1 R | CGGATTCTTTGAGTGAAGCAATGG |
| EVD "exon2" siRNA/mRNA | GAG-ex2 F | TATTGAGACGGGAAAAGTTATTGG |
| | GAG-ex2 R | CATCCGAATGAACTAGATCAAAAC |
| EVD intron siRNA/mRNA | GAG-in F | GTATCACTTTCTCATCACAAACATC |
| | GAG-in R | CTGAAAATACACATCATTAGGG |
| EVD RT (POL) siRNA | RT F | CAAAGACGGTATAGACTCTACCAAGAC |
| | RT R | CTCTATACTCCGATTCTGCACTCGAACA |
| EVD LTR siRNA | LTR F | TTGATCAAGACTCAAATAAGAAAGGCC |
| | LTR R | TATGCTCTGATACCATGAAAGAATAT |
| GFP siRNA/mRNA | GFP F | GCCGACAGTGGTCCCAAAGATG |
| | GFP R | TGATATCACTAGTGCGGCCG |
| GUS siRNA/mRNA | GUS F | GTGTGATATCTACCCGCTTCGCGTC |
| | GUS R | AAAGAGAGGTTAAAGCCGACAGCAGC |
| ACT2 mRNA | ACT2 F | GCACCCTGTTCTTCTTACCG |
| | ACT2 R | AACCCTCGTAGATTGGCACA |
| Athila6A siRNA | Athila6A F | caacgtcgatgaagctgaatcttgg |
| | Athila6A R | cctctcgaacttgggtttagttcc |
| Oligo probes | miR159 | TAGAGCTCCCTTCAATCCAAA |
| | miR171 | GATATTGGCGCGGCTCAATCA |
| | miR160 | TGGCATACAGGGAGCCAGGCA |
| | miR166 | GGGGAATGAAGCATGGTCCGA |
| | mir173 | GTGATTTCTCTCTGCAAGCGAA |
| | mir168 | TTCCCGACCTGCACCAAGCGA |
| | tasiR255 | TACGCTATGTTGGACTTAGAA |
| | U6 | AGGGGCCATGCTAATCTTCTC |

**5'OH RACE**

| Use | Primer | Sequence 5'->3' |
|---|---|---|
| 5' RNA adaptors | RNA adapter 5' dT | /5InvddT/ACGAUCAGUUCGCCGAUGCAG |
| | RNA adapter 3-P | ACCGCCGACGUCGAGGUGCCCGAAGGACCGCGCA-<br>-CCUGGUGCAUGACCCGCAAGCCCGGU/3Phos/ |
| EVD Gene-specific RT oligo | EVD_RT_Rv | CTAGCCTAGCATGCCACAAAG |
| Forward nested primers | Outer_Fw | ACCGCCGACGTCGAGGTGCC |
| | Inner_Fw | CGAAGGACCGCGCACCTGGT |
| EVD GAG region 1 RACE | EVD_R1_Out_Rv | AATCTTAGAACACACCTCATC |
| | EVD_R1_Inn_Rv | TTGGTAACTTCTCCGAC |
| EVD GAG region 2 RACE | EVD_R2_Out_Rv | CCGGCTTGACTTTGCTCCTC |
| | EVD_R2_Inn_Rv | GAGTTTCTTGAGAGAGATGAG |
| EVD GAG region 3 RACE | EVD_R3_Out_Rv | GACTTAGAGGAAAAACAGCTAG |
| | EVD_R3_Inn_Rv | AGAGTTTGGTGACAAGTCTTC |

**qPCR primers**

| Target | Primer | Sequence 5'->3' |
|---|---|---|
| Total GAG/EVD mRNA - | qGag-F | TTTGACCCGCGTGTTTGAAG |
| copy number | qGag-R | AATCTTCGGGTCAAGCGTTC |
| EVD flGAGPOL mRNA - | qRT-F | ACATATGGCTCCGACTACATGG |
| copy number | qRT-R | AGTTCGCCTTGGAGAAATGC |
| ACT2 mRNA | qACT2-F | GCACCCTGTTCTTCTTACCG |
| | qACT2-R | AACCCTCGTAGATTGGCACA |
| GAPC mRNA | qGAPC-F | ACTCAATCACTGCTACTCAG |
| | qGAPC-R | GTTGGGACACGGAAAGACATTCCA |
| RHIP1 mRNA | qRHIP1-F | GAGCTGAAGTGGCTTCAATGAC |
| | qRHIP1-R | GGTCCGACATACCCATGATCC |
| Spliced EVD/shGAG | qGAG spliced F | GTTGGTTGCTACATCCACACCT |
| mRNA | qGAG spliced R | TCAATATCCGGATTCTTTGAG |
| unspliced EVD/flGAG- | qGag unspliced F | GTTGGTTGCTACATCCACACCT |
| POL mRNA | qGag unspliced R | CAATTGAACTACTAGATGTTTGTGATG |
| Spliced GFP-EVDi- | qGFP spliced F | GCTGCTGGGATTACACATGGC |
| GUS/shGFP mRNA | qGFP spliced R | TCAATATCCGGATTCTTTGAG |
| Unspliced GFP-EVDi- | qGUS unspliced F | GCTGCTGGGATTACACATGGC |
| GUS/flGFP-GUS mRNA | qGUS unspliced R | CAATTGAACTACTAGATGTTTGTGATG |
| flGFP-GUS mRNA | qGUS F | GCACGGGAATATTTCGCGC |
| | qGUS R | GTATCGGTGTGAGCGTCGC |
| 18S rRNA | q18S F | TAGTTGGTGGAGCGATTTGTCTG |
| | q18S R | CTAAGCGGCATAGTCCCTCTAAG |
| U5 snoRNA | qU5 F | GAATACCGTGTGCTCTCCACGCT |
| | qU5 R | CCTCCAAAAATAGGCGTATGCCAC |
| MYB65 mRNA | qMYB65 F | GATGGTTCCTGATAGCCATACAGTTAC |
| | qMYB65 R | TAGGCATCAACAGAGTCAAGGAGATC |
| HAP2B mRNA | qHAP2B F | CTTGAACACTAAAAGTCAGAACTTGG |
| | qHAP2B R | GACACATTTAATCCGTTCGATAAGTT |
| TAS1c mRNA | qTAS1c F | TGTAGCGAAGAAGCATCA |
| | qTAS1c R | TGCAAAGCAAAACAGAAG |
| TAS3 mRNA | qTAS3 F | GAGACCGAAGTTTCTCCAAGGC |
| | qTAS3 R | CAGCACAGCGGATCCAATAATCTC |
| ARF4 mRNA | qARF4 F | ATACTACCCCACCCGGAAAC |
| | qARF4 R | TGAGACTGCATCGCAAAATC |
| HTT2 mRNA | qHTT2 F | GCCTGTCTAGCCTGTCTCGT |
| | qHTT2 R | CCCTCGGACTTATTCACTGC |
