# Supplementary material for: Innate, translation‐dependent silencing of an invasive transposon in Arabidopsis
Source: EMBO Rep. 2021 Dec 21;23(3):e53400. doi: 10.15252/embr.202153400 (PMC8892269; doi:10.15252/embr.202153400)
Supplement: Supplementary file 3 — Source Data for Figure 1 [file EMBR-23-e53400-s007.zip › Figure 1/1D/qRT-PCR plate - Sample-Probe distribution on a 384 well plate-.pdf]

qRT-PCR PLATE

		qGAG			qGAG spl			qGAG uspl			qIN			qRT			qGAPC			qACT2			qACT2		
		1	2	3	4	5	6	7	8	9	10	11	12	13	14	15	16	17	18	19	20	21	22	23	24
Col-0#1	A																								
Col-0#2	B																								
Col-0#3	C																								
35S:EVD(Col)#1	D																								
35S:EVD(Col)#2	E																								
35S:EVD(Col)#3	F																						RT-		
rdr6-12#1	G																								
rdr6-12#2	H																								
rdr6-12#3	I																								
35S:EVD(rdr6)#1	J																								
35S:EVD(rdr6)#2	K																								
35S:EVD(rdr6)#3	L																								
	M																								
	N																								
	O																								
	P																								
