# Supplementary figures and images for: Innate, translation‐dependent silencing of an invasive transposon in Arabidopsis
Source: EMBO Rep. 2021 Dec 21;23(3):e53400. doi: 10.15252/embr.202153400 (PMC8892269; doi:10.15252/embr.202153400)

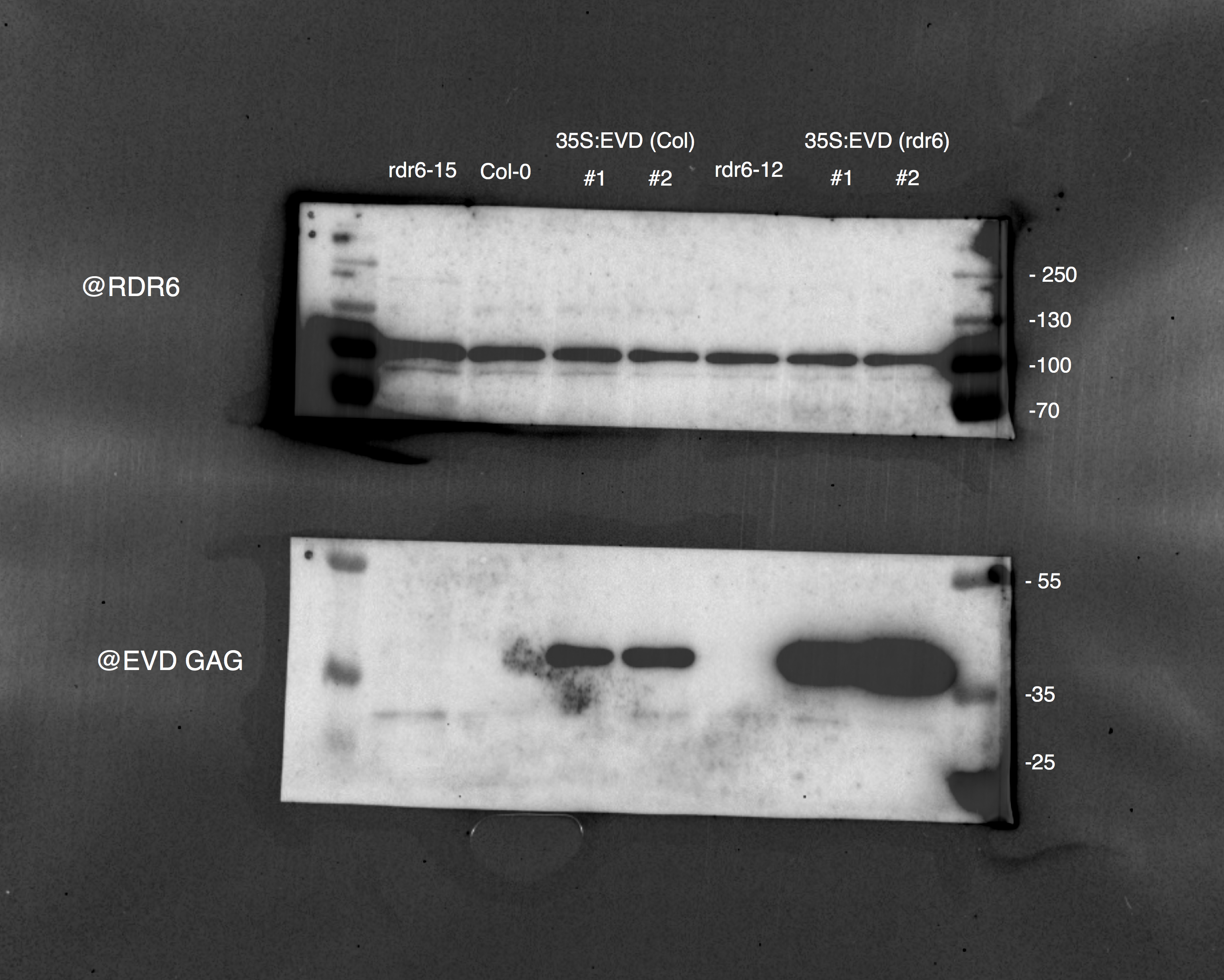

Supplement: Supplementary file 3 — Source Data for Figure 1 [file EMBR-23-e53400-s007.zip › Figure 1/1E/Overlay+sample annotation.tif]

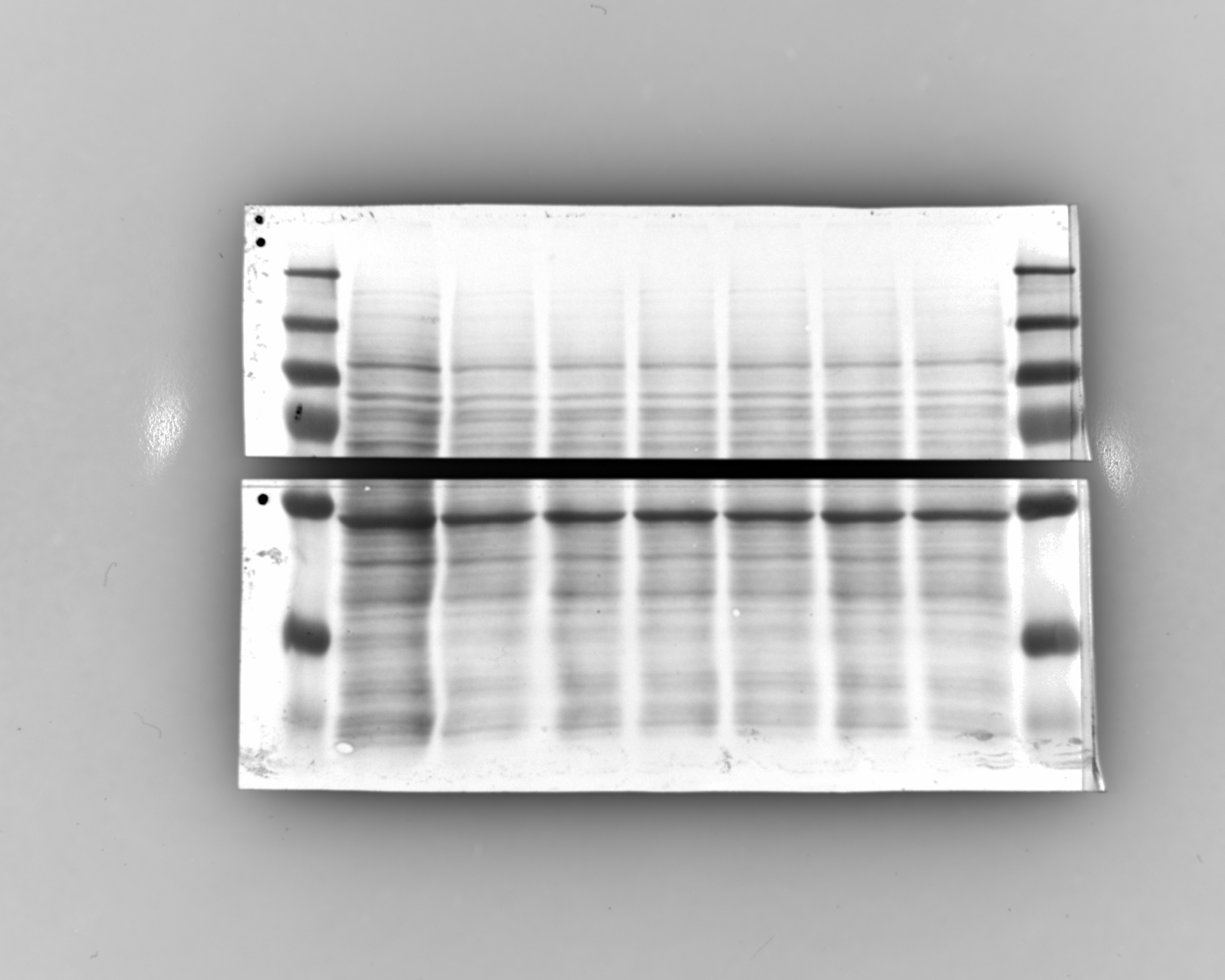

Supplement: Supplementary file 3 — Source Data for Figure 1 [file EMBR-23-e53400-s007.zip › Figure 1/1E/Coomassie staining.tif]

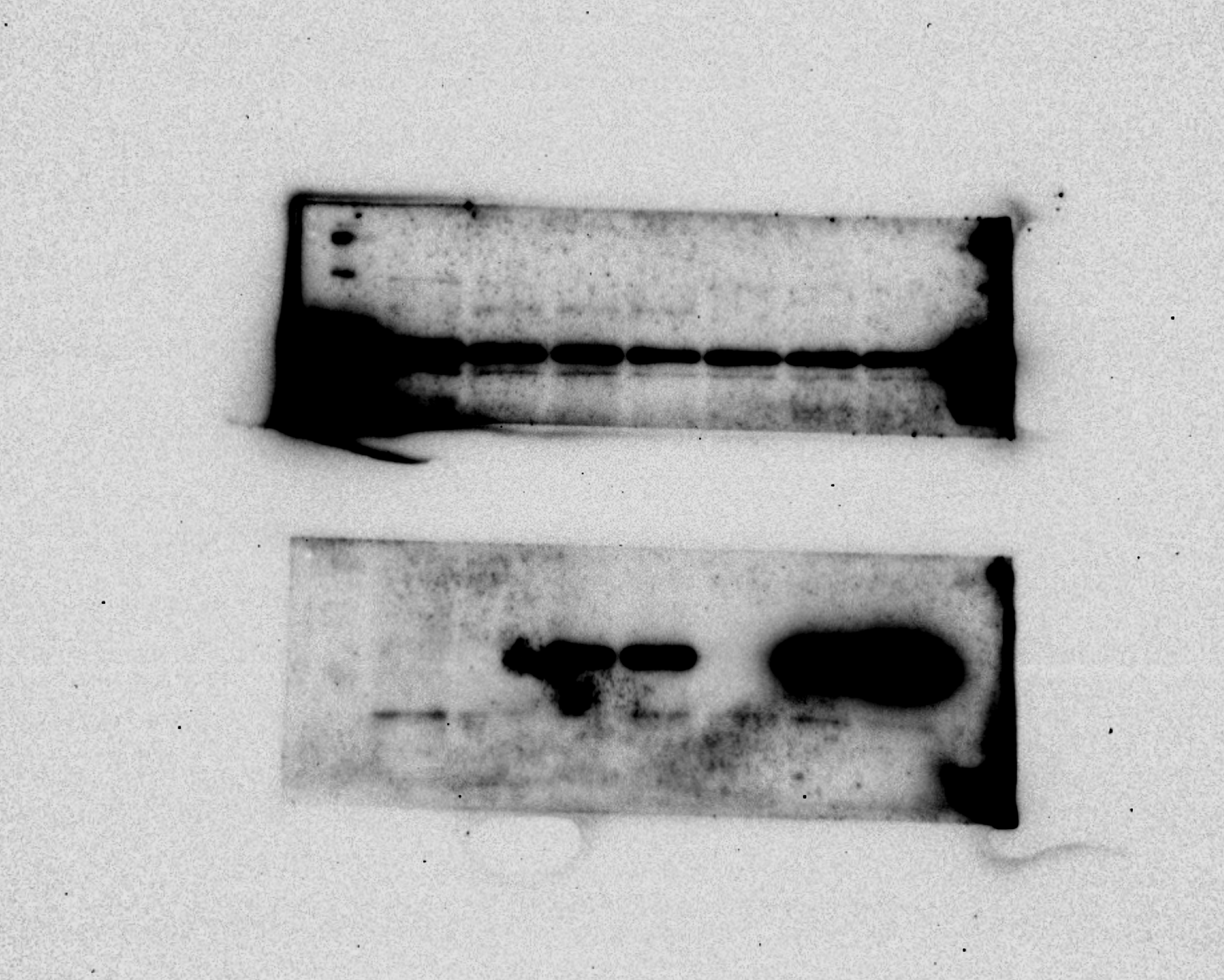

Supplement: Supplementary file 3 — Source Data for Figure 1 [file EMBR-23-e53400-s007.zip › Figure 1/1E/WB @ GAG + RDR6 exp2.tif]

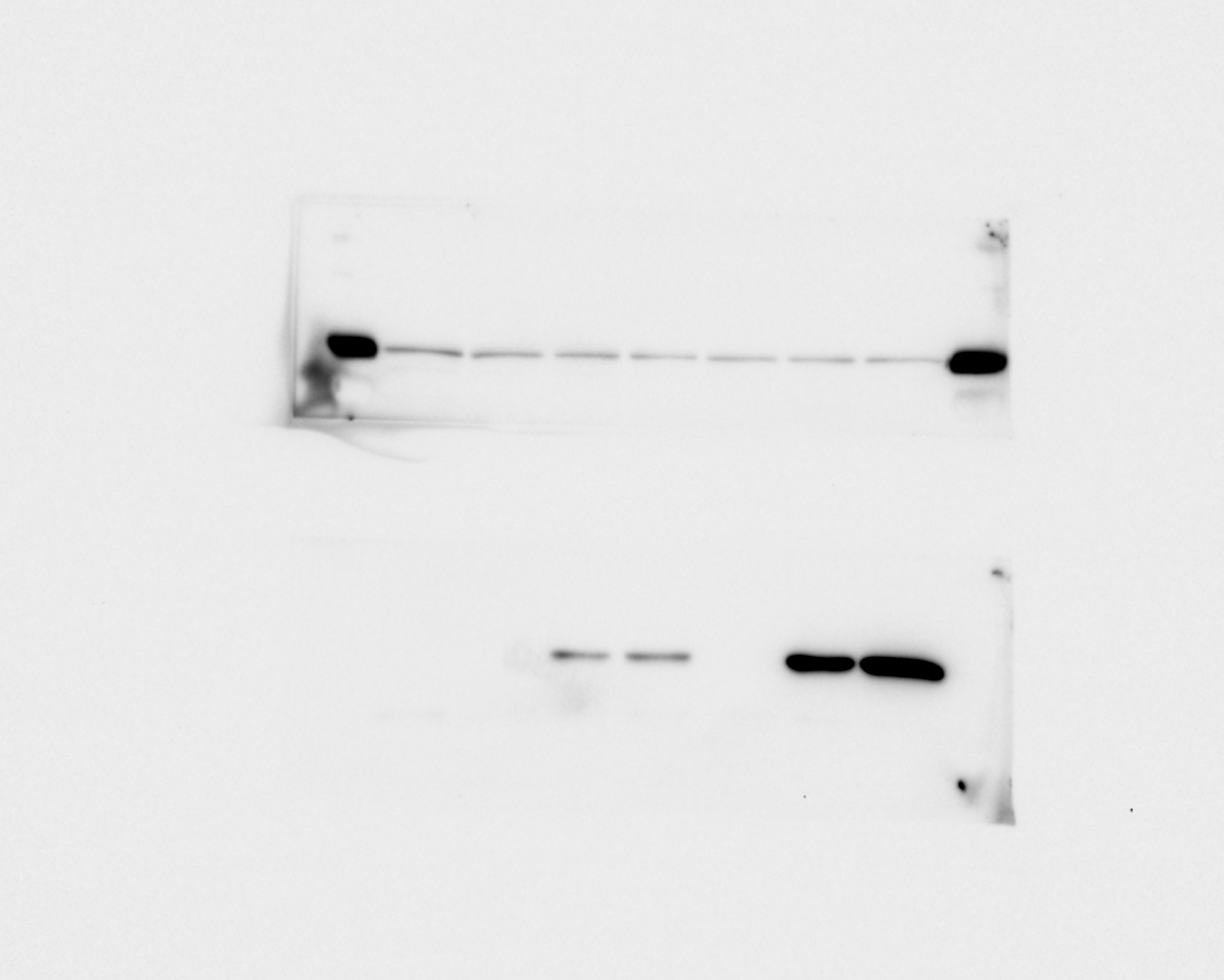

Supplement: Supplementary file 3 — Source Data for Figure 1 [file EMBR-23-e53400-s007.zip › Figure 1/1E/WB @ GAG + RDR6 Exp1.tif]

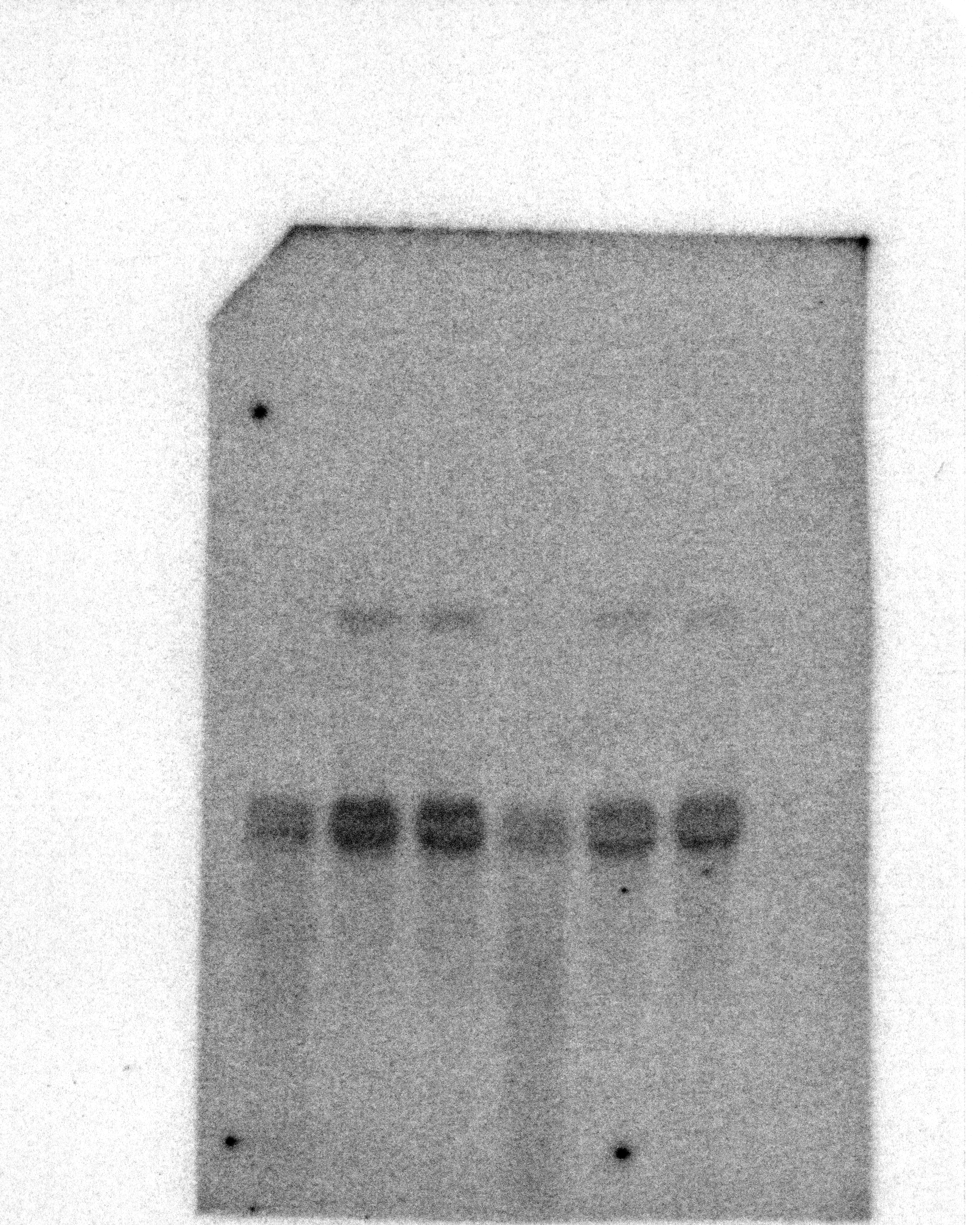

Supplement: Supplementary file 3 — Source Data for Figure 1 [file EMBR-23-e53400-s007.zip › Figure 1/1C/HMW-NB @ ACT2.tif]

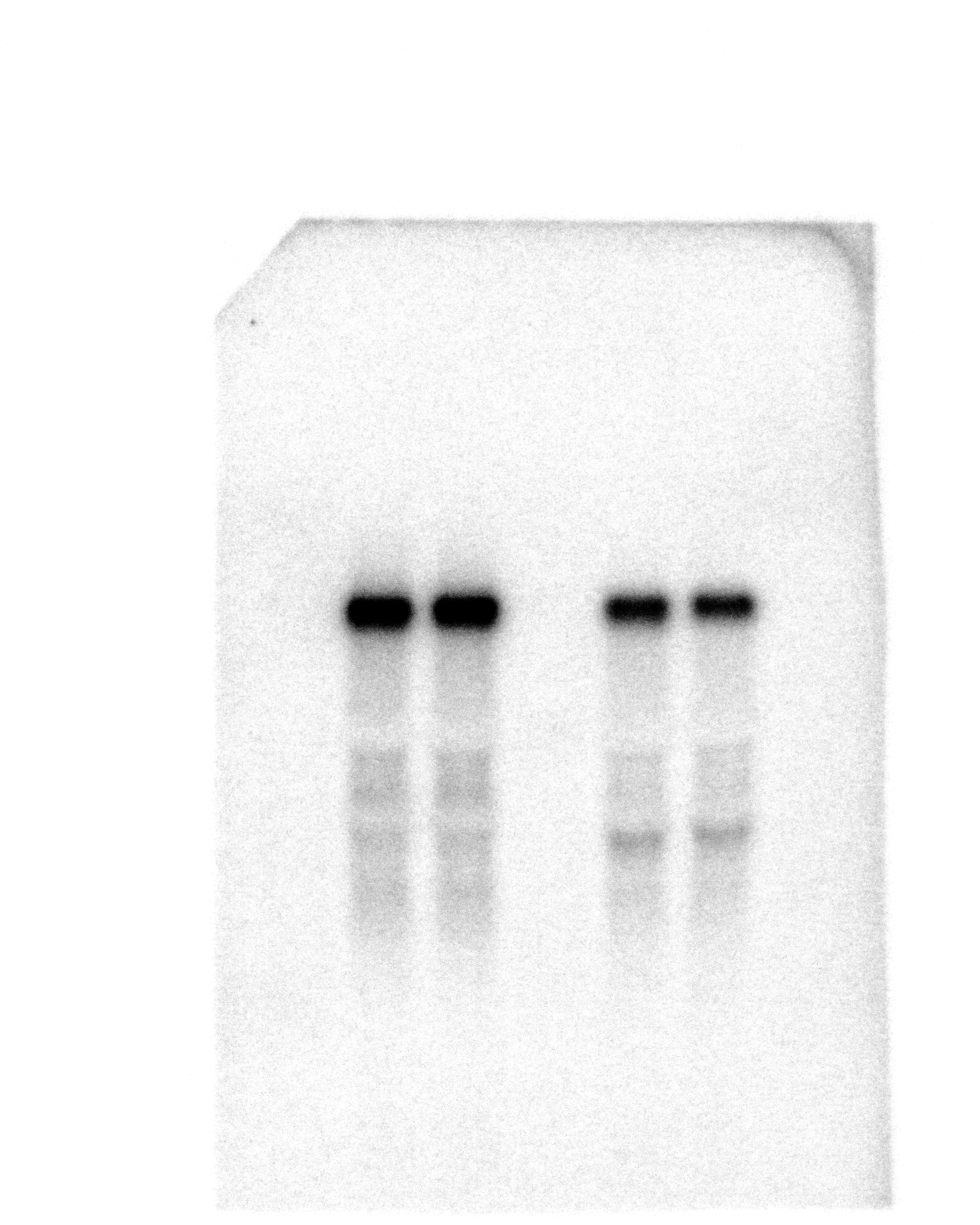

Supplement: Supplementary file 3 — Source Data for Figure 1 [file EMBR-23-e53400-s007.zip › Figure 1/1C/HMW-NB @ GAG.tif]

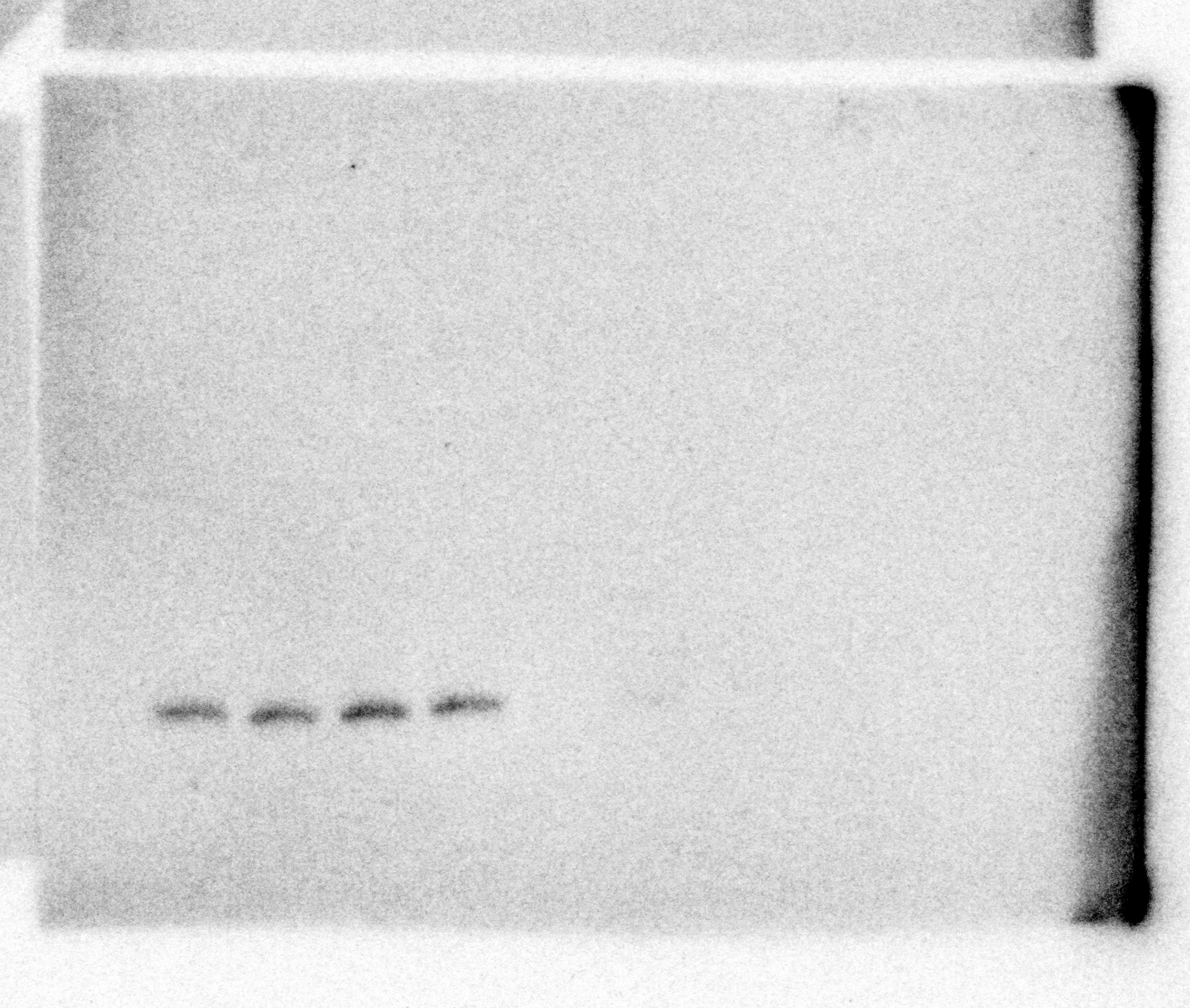

Supplement: Supplementary file 4 — Source Data for Figure 2 [file EMBR-23-e53400-s006.zip › Figure 2/2D/GEG T1 LMW @TAS1-[Phosphor].tif]

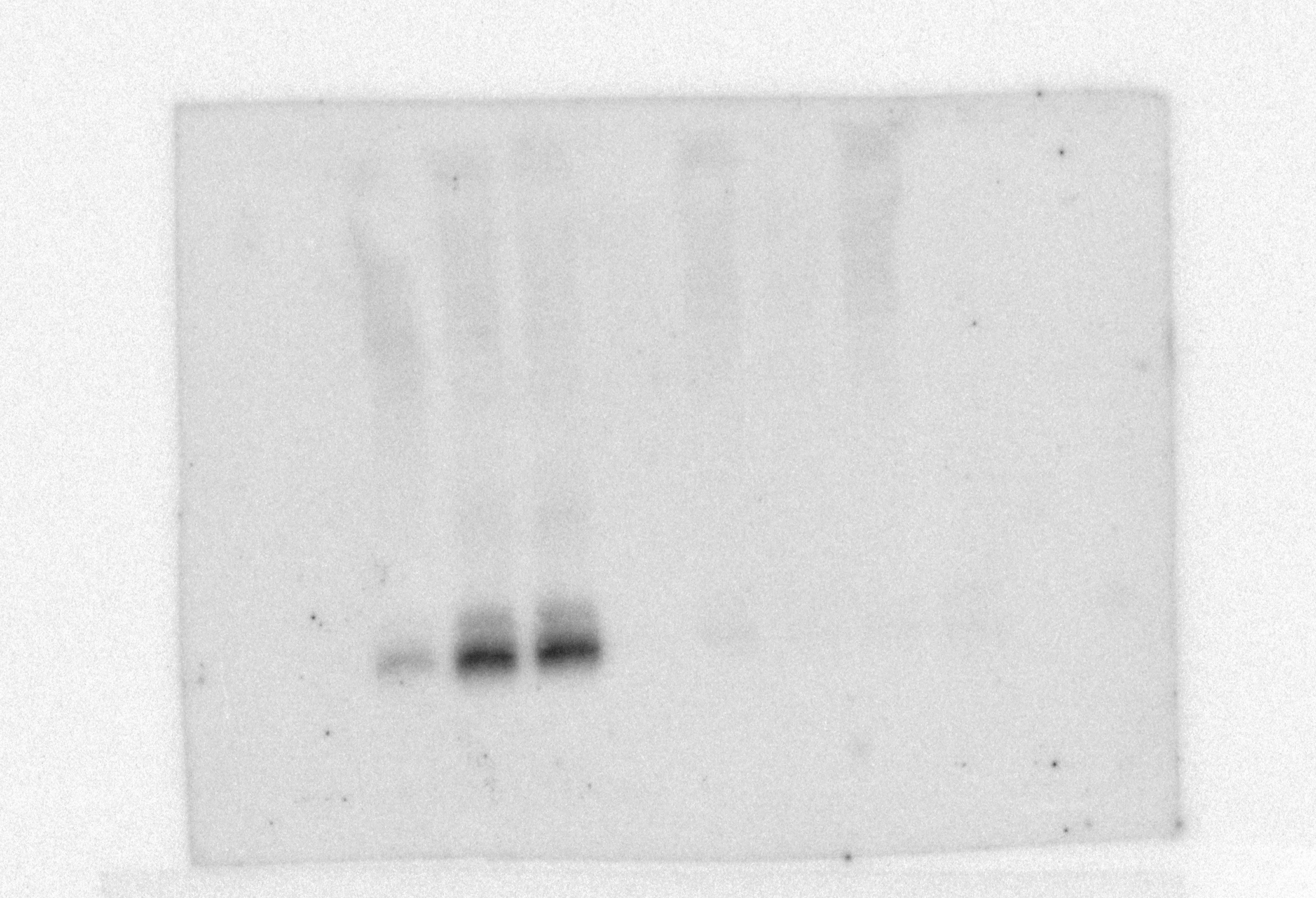

Supplement: Supplementary file 4 — Source Data for Figure 2 [file EMBR-23-e53400-s006.zip › Figure 2/2D/GEG T1 LMW @3GFP 180418-[Phosphor].tif]

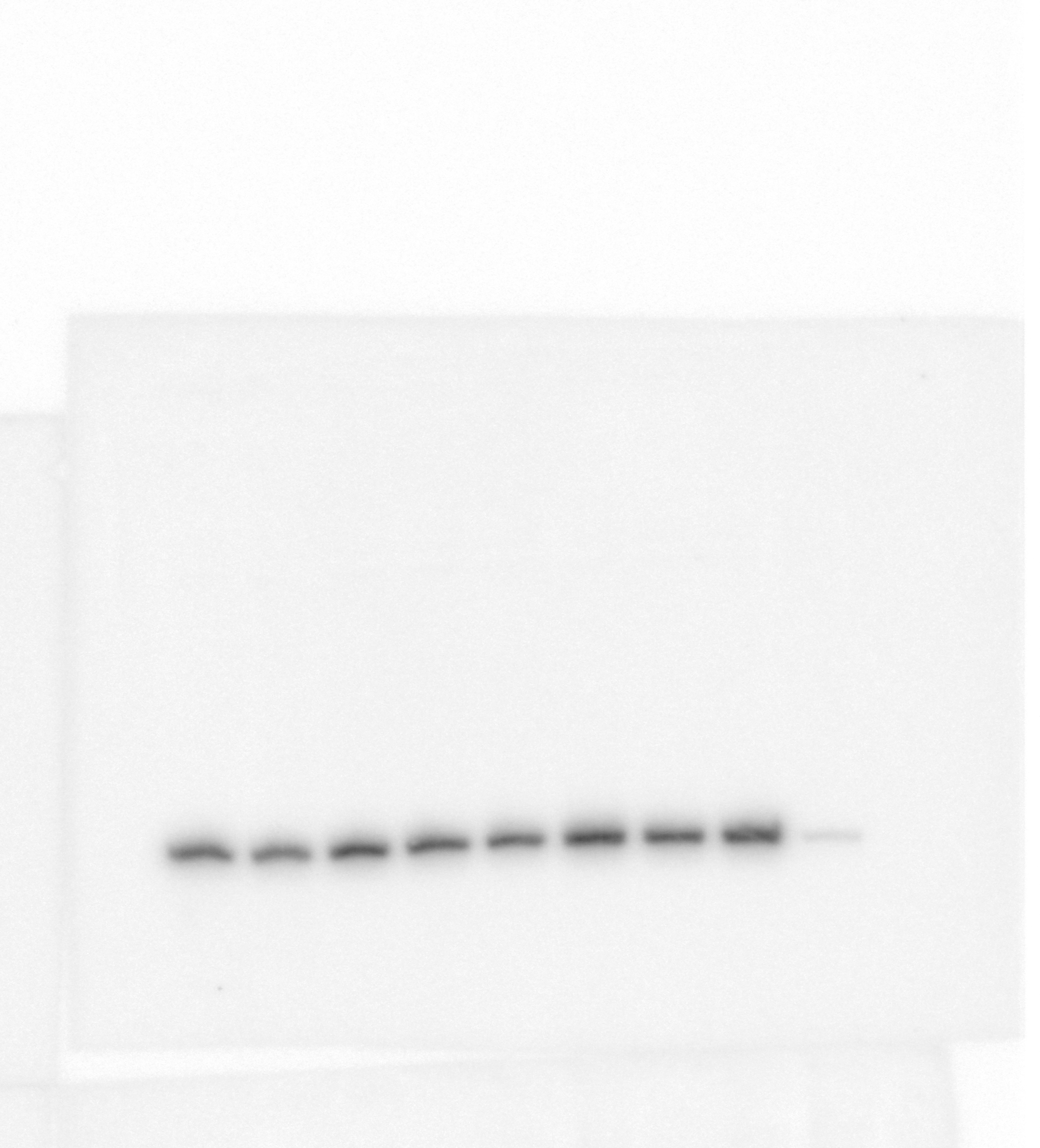

Supplement: Supplementary file 4 — Source Data for Figure 2 [file EMBR-23-e53400-s006.zip › Figure 2/2D/GEG T1 LMW @159 260317-[Phosphor].tif]

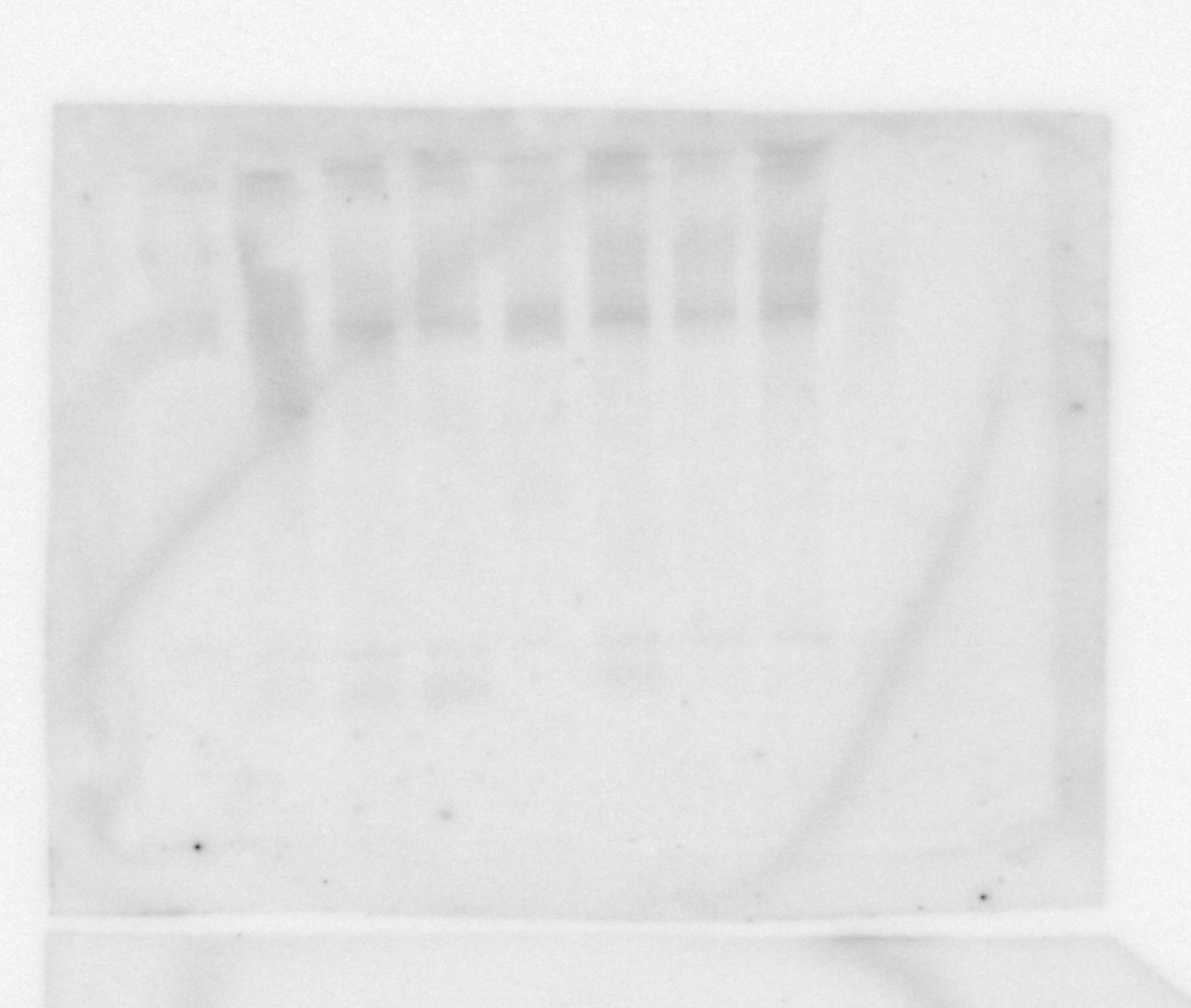

Supplement: Supplementary file 4 — Source Data for Figure 2 [file EMBR-23-e53400-s006.zip › Figure 2/2D/GEG T1 LMW @GUS3 070318-[Phosphor].tif]

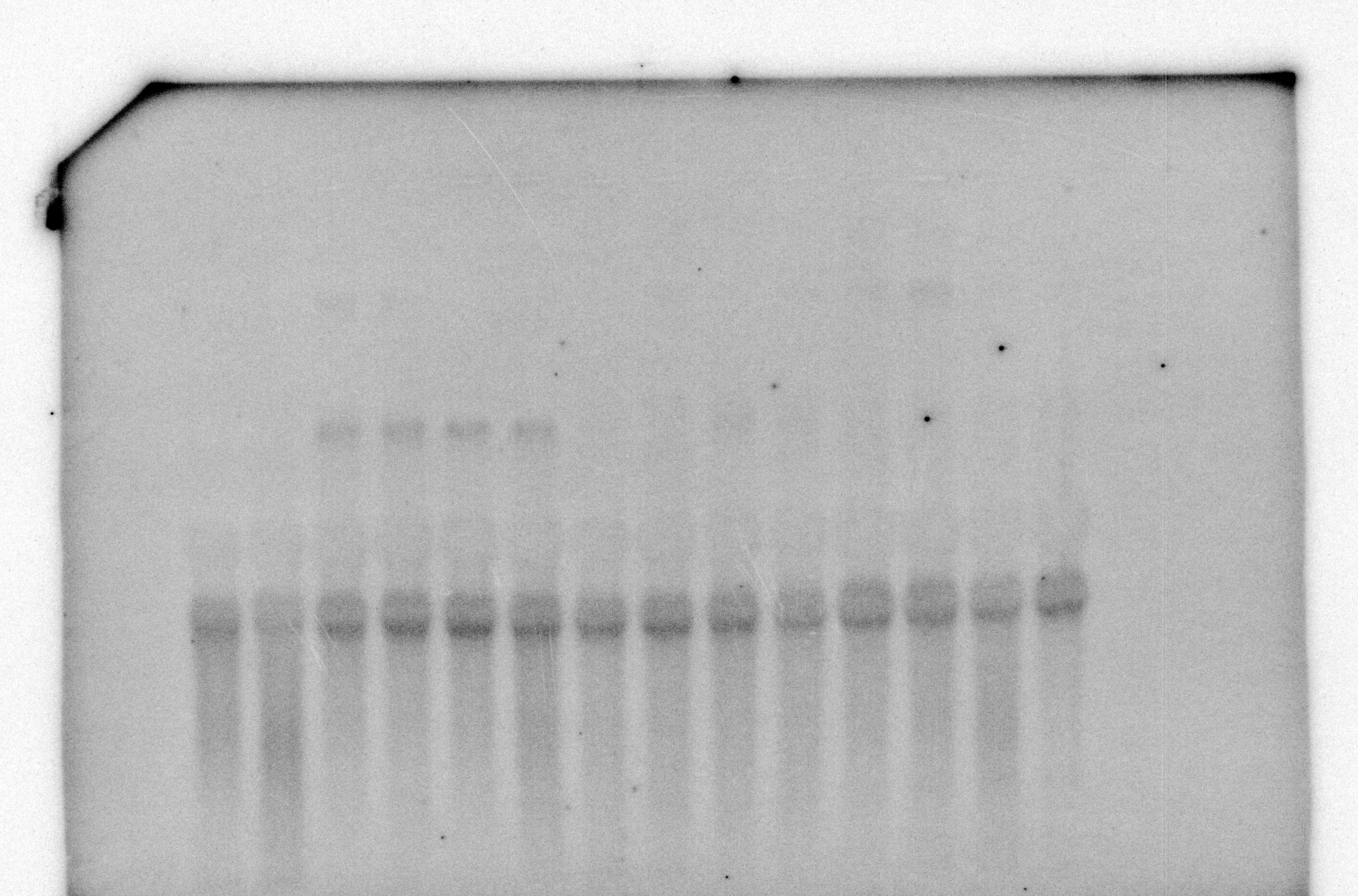

Supplement: Supplementary file 5 — Source Data for Figure 3 [file EMBR-23-e53400-s002.zip › Figure 3 (1:2)/3E/HMWNB EVD muts @ ACT2 .tif]

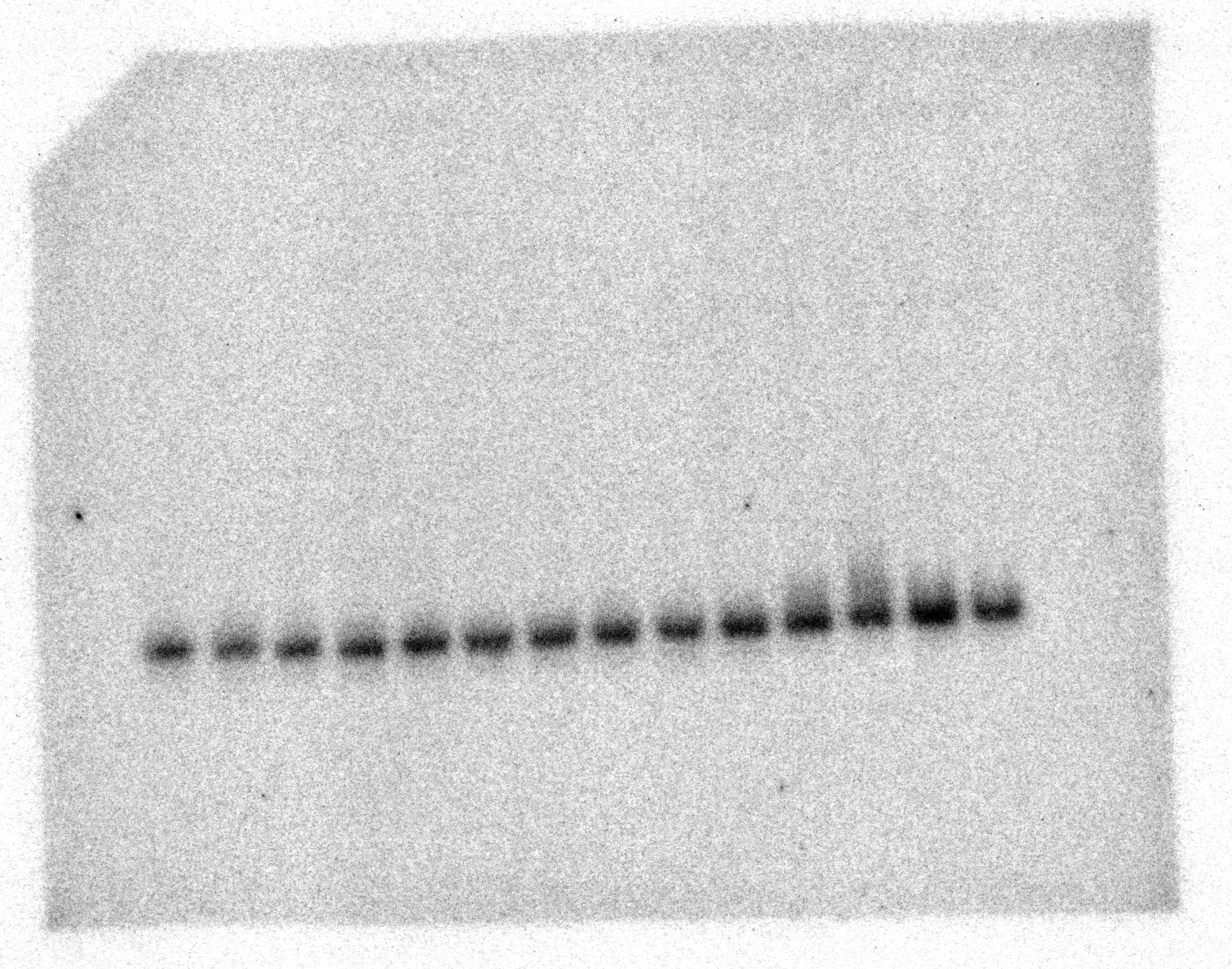

Supplement: Supplementary file 5 — Source Data for Figure 3 [file EMBR-23-e53400-s002.zip › Figure 3 (1:2)/3E/LMW-NB EVD muts @. miR173.tif]

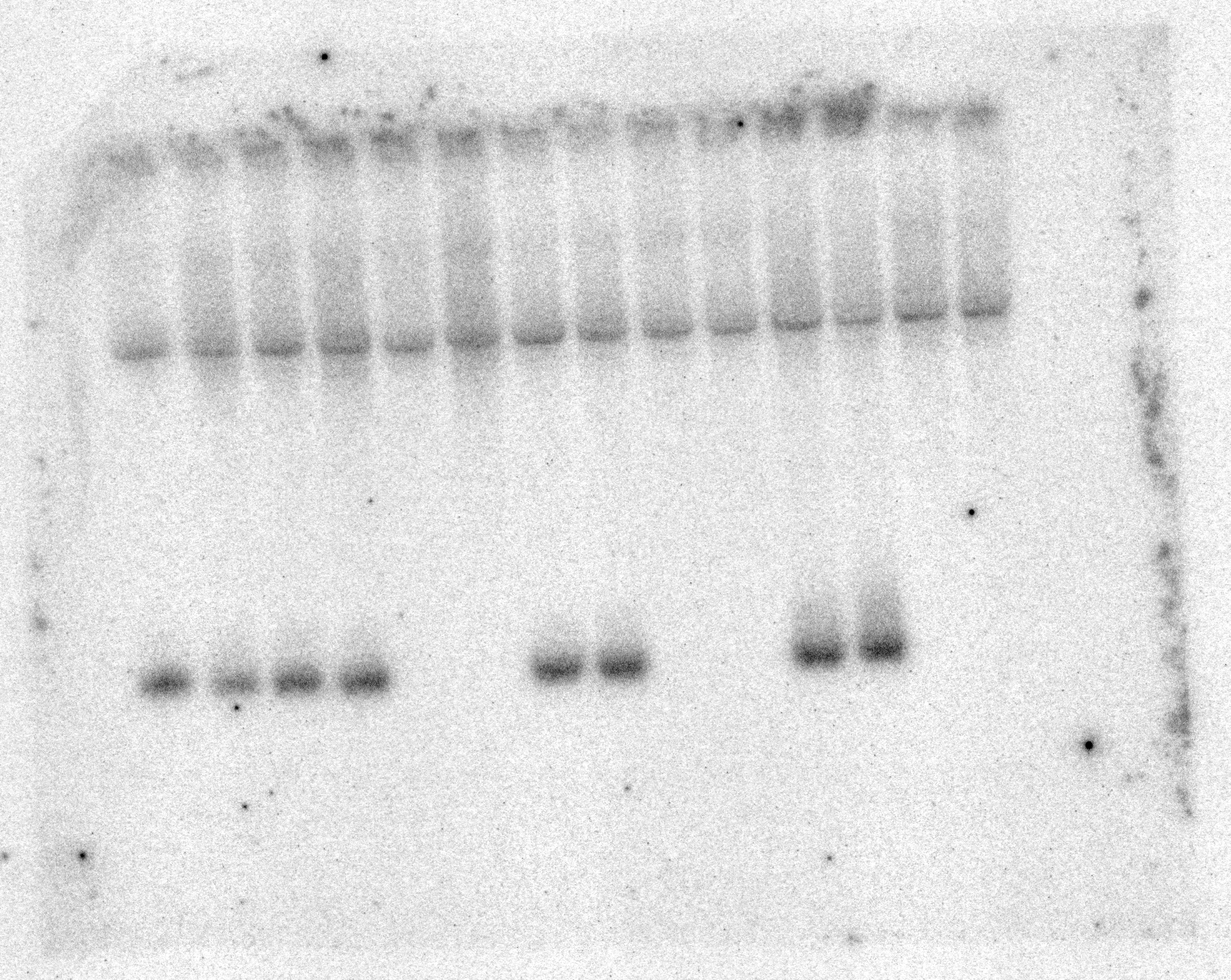

Supplement: Supplementary file 5 — Source Data for Figure 3 [file EMBR-23-e53400-s002.zip › Figure 3 (1:2)/3E/LMW-NB EVD muts @TAS255 + U6.tif]

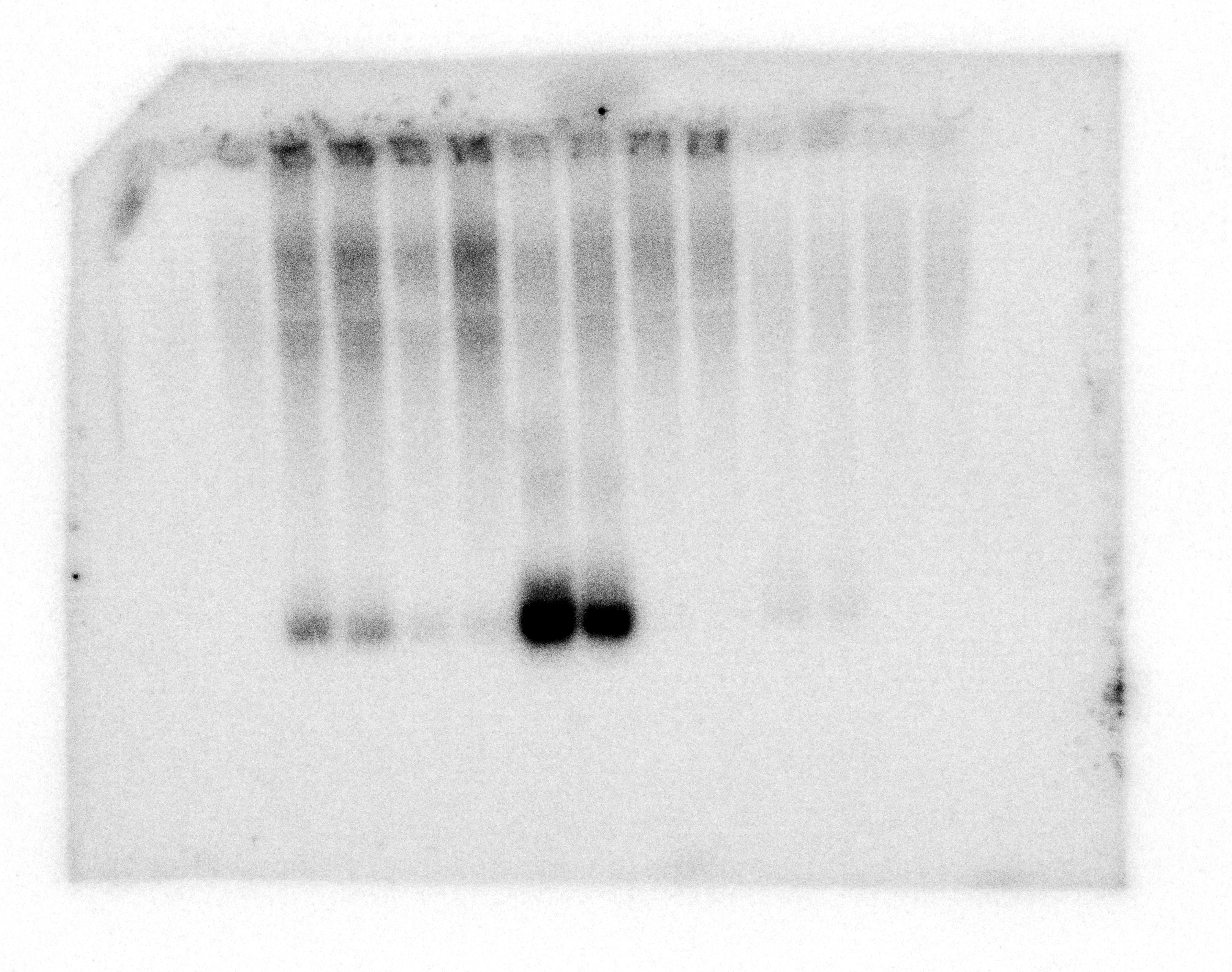

Supplement: Supplementary file 5 — Source Data for Figure 3 [file EMBR-23-e53400-s002.zip › Figure 3 (1:2)/3E/LMW-NB EVD muts @ GAG-in.tif]

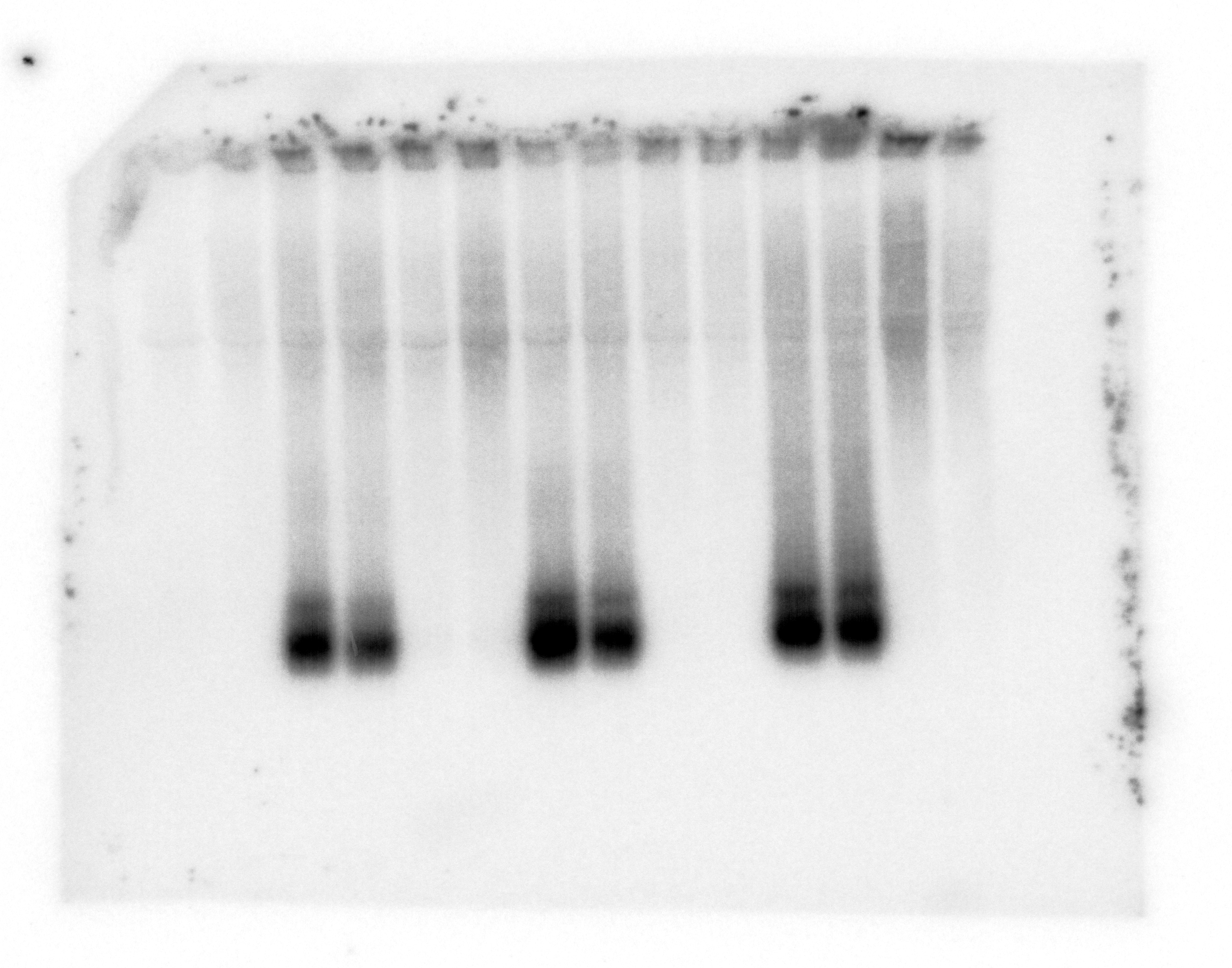

Supplement: Supplementary file 5 — Source Data for Figure 3 [file EMBR-23-e53400-s002.zip › Figure 3 (1:2)/3E/LMW-NB EVD muts @ GAG-ex1.tif]

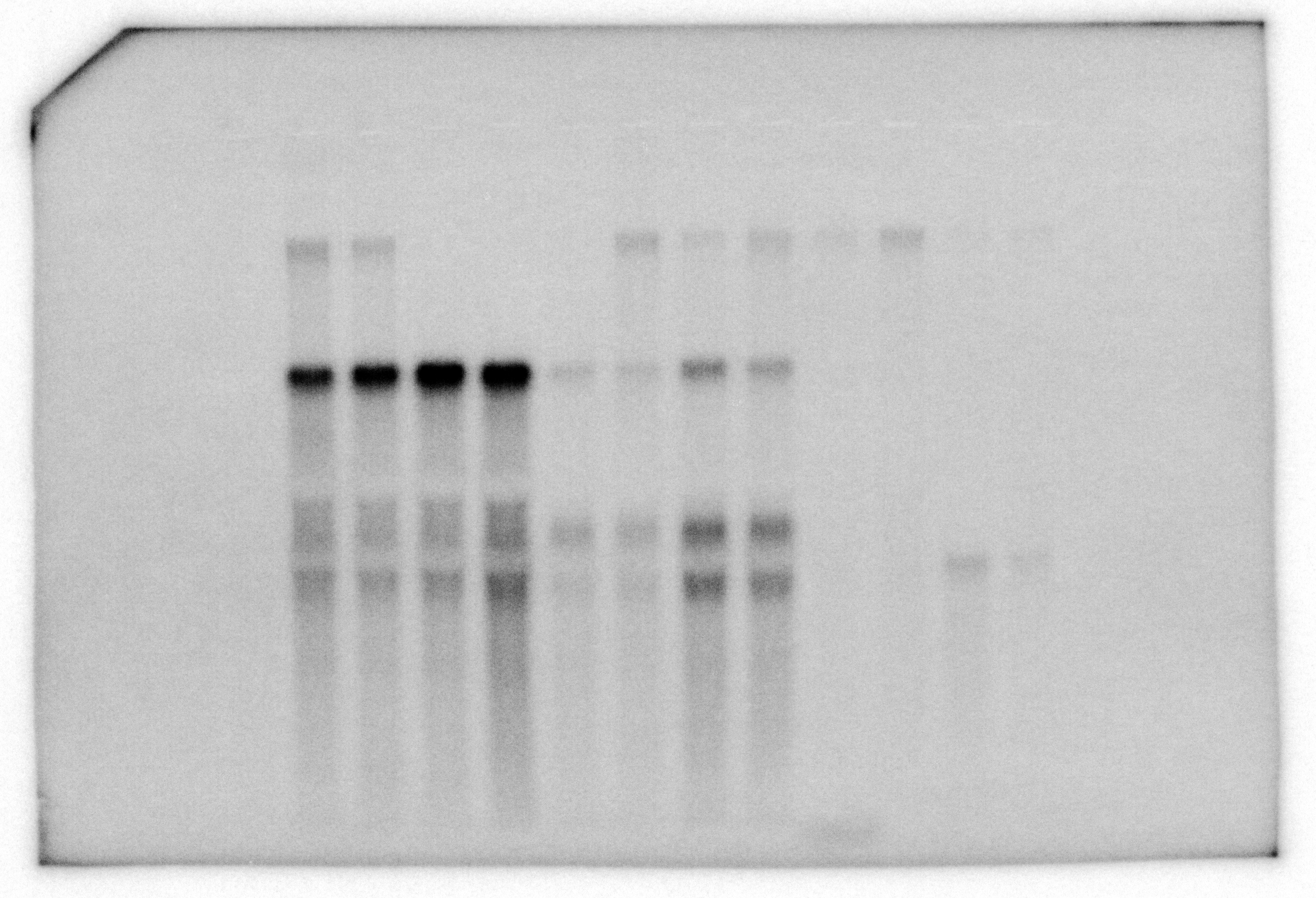

Supplement: Supplementary file 5 — Source Data for Figure 3 [file EMBR-23-e53400-s002.zip › Figure 3 (1:2)/3E/HMWNB EVD muts @ GAGin.tif]

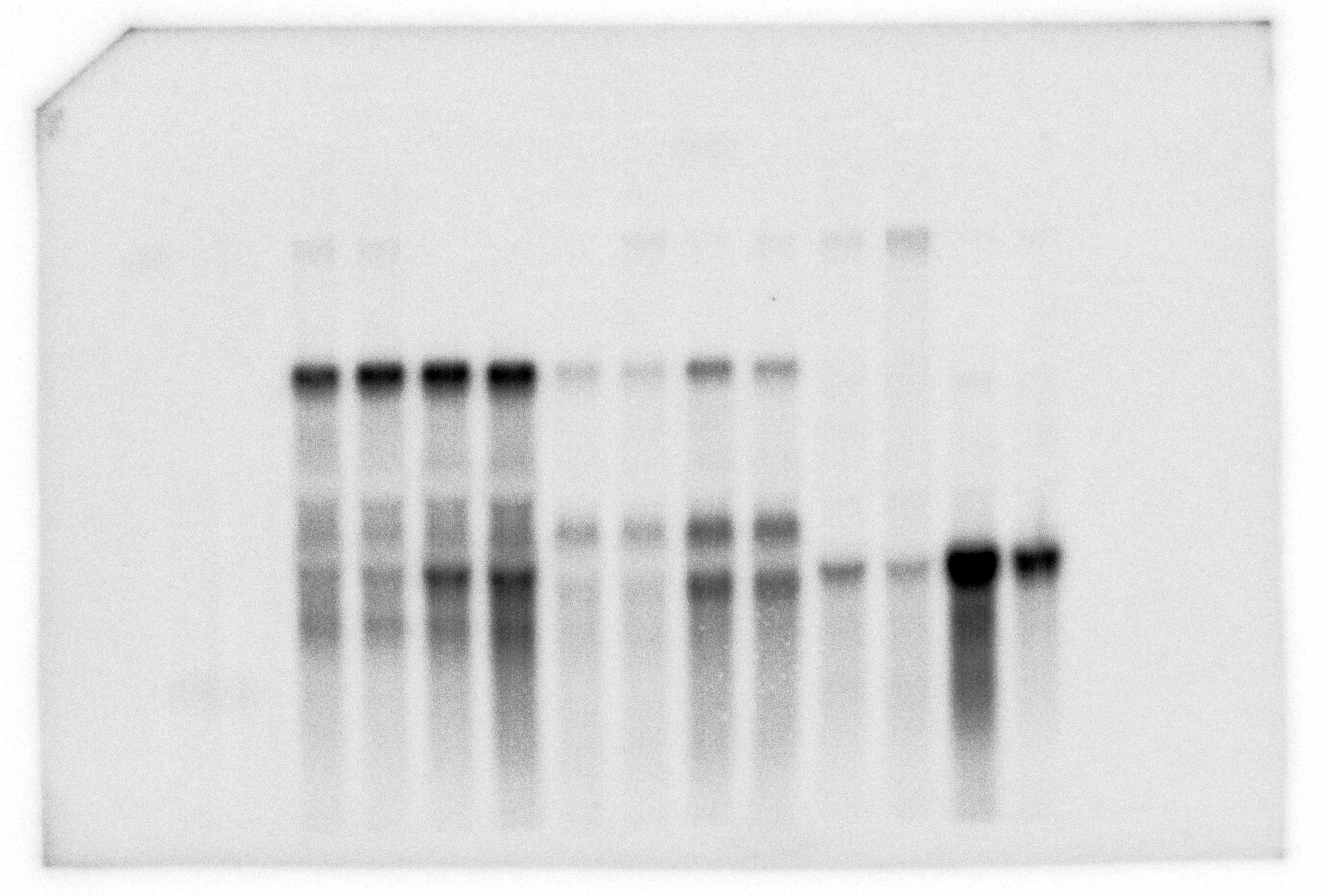

Supplement: Supplementary file 5 — Source Data for Figure 3 [file EMBR-23-e53400-s002.zip › Figure 3 (1:2)/3E/HMWNB EVD muts @ GAGex1.tif]

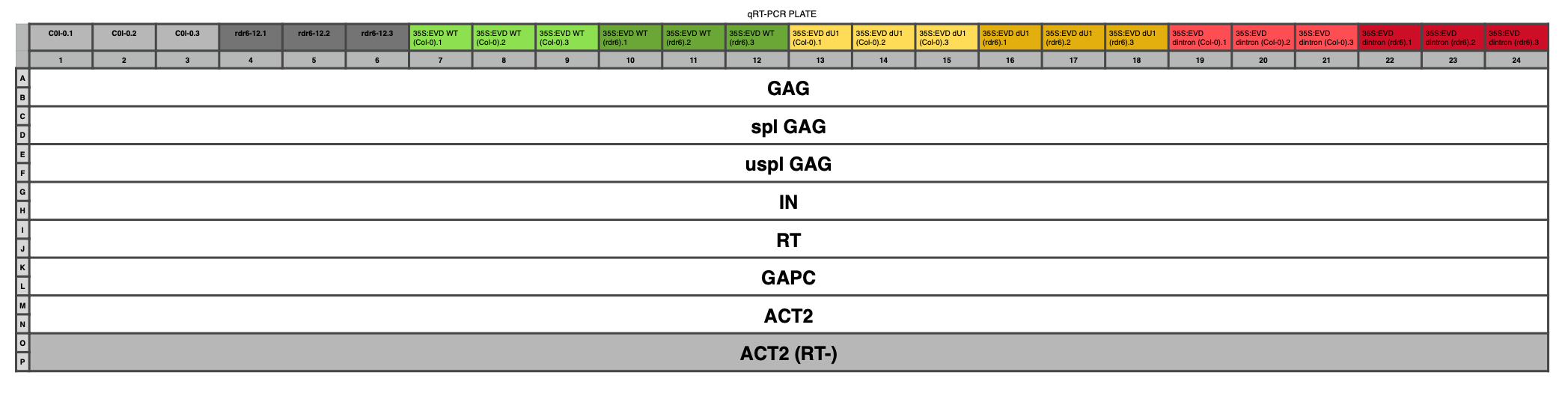

Supplement: Supplementary file 5 — Source Data for Figure 3 [file EMBR-23-e53400-s002.zip › Figure 3 (1:2)/3D/qPCR plate distribution.png]

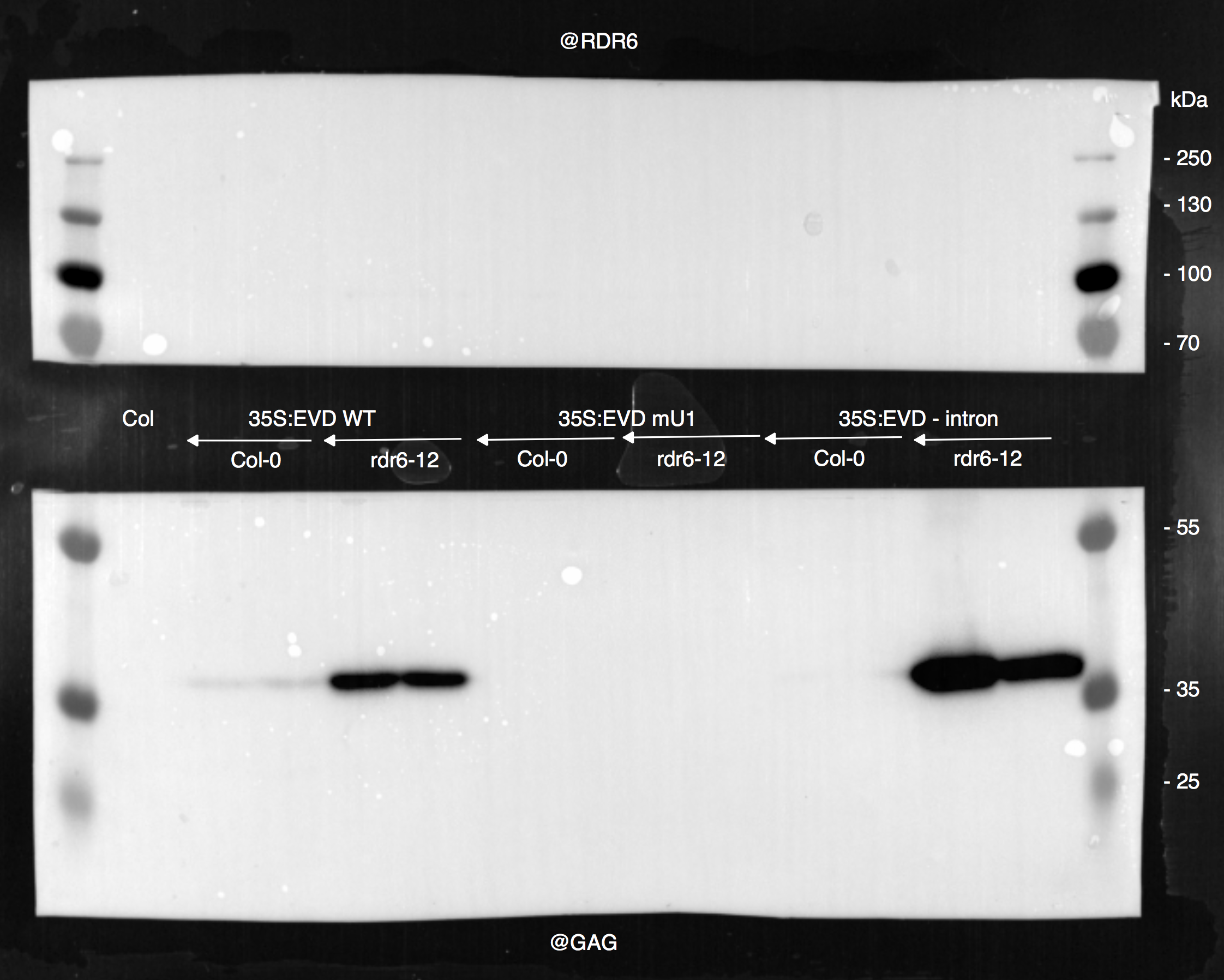

Supplement: Supplementary file 5 — Source Data for Figure 3 [file EMBR-23-e53400-s002.zip › Figure 3 (1:2)/3F/merged - Sample-antibody distribution on membrane.tif]

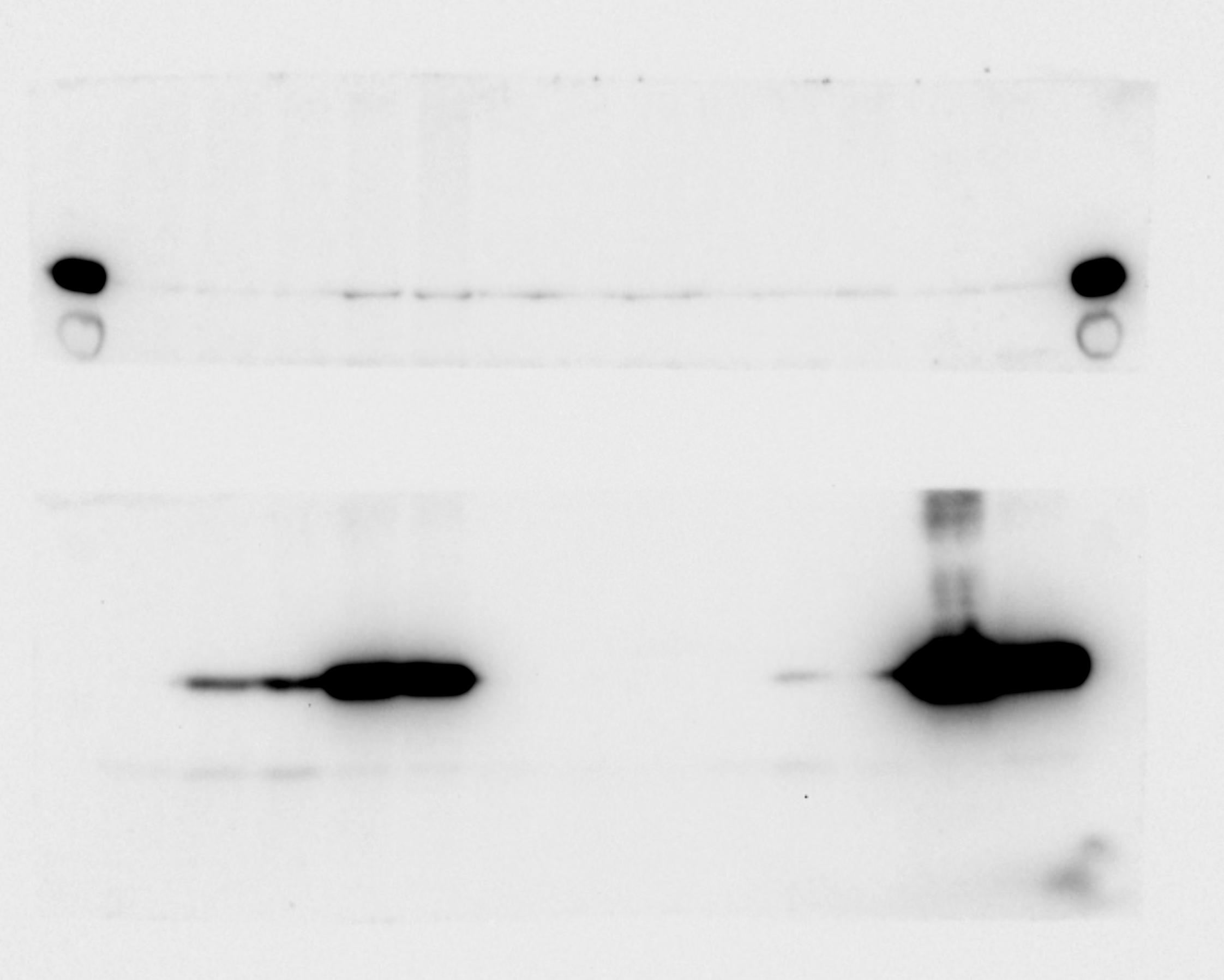

Supplement: Supplementary file 5 — Source Data for Figure 3 [file EMBR-23-e53400-s002.zip › Figure 3 (1:2)/3F/WB EVD muts @ Gag.tif]

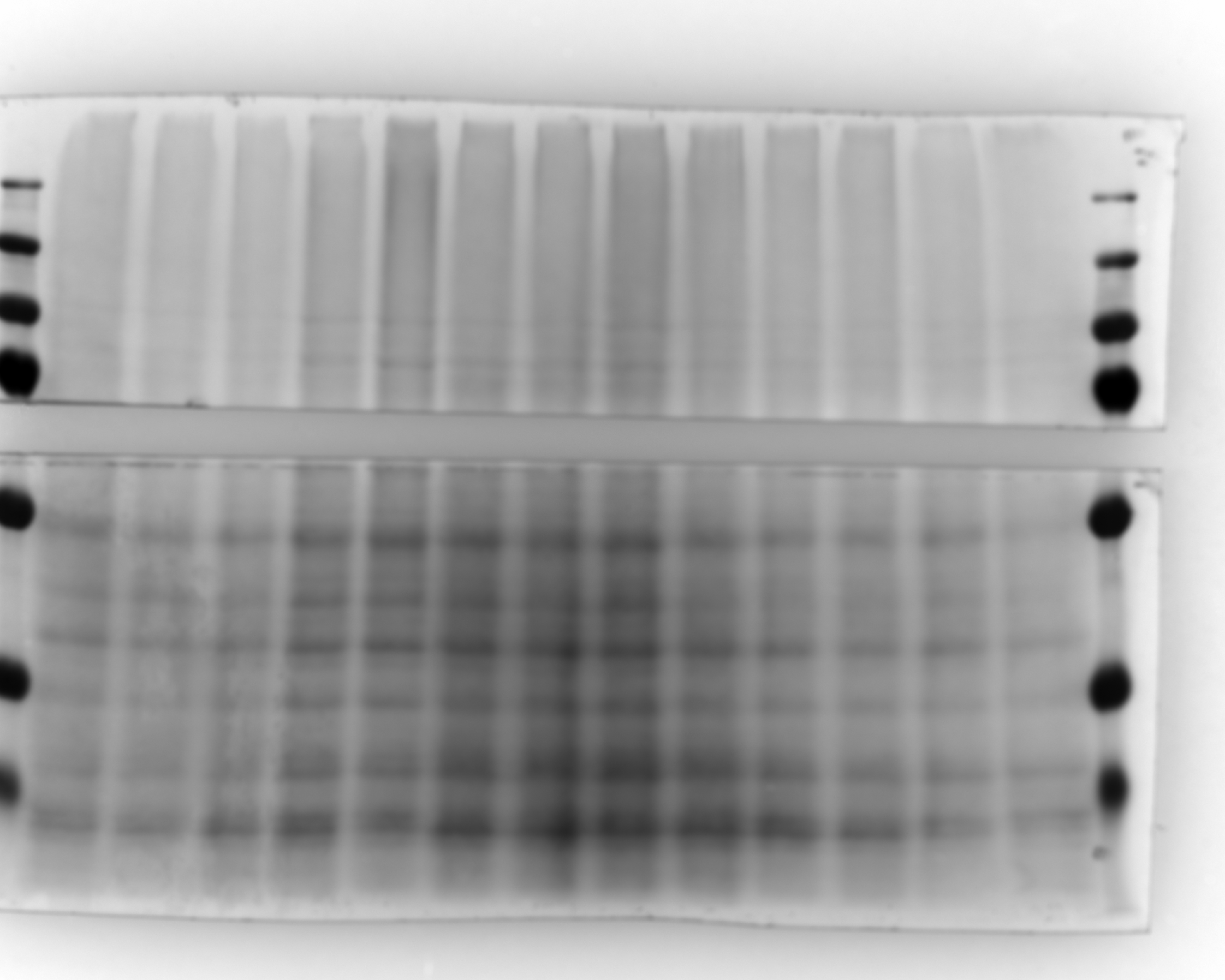

Supplement: Supplementary file 5 — Source Data for Figure 3 [file EMBR-23-e53400-s002.zip › Figure 3 (1:2)/3F/WB EVD muts Coom staining.tif]

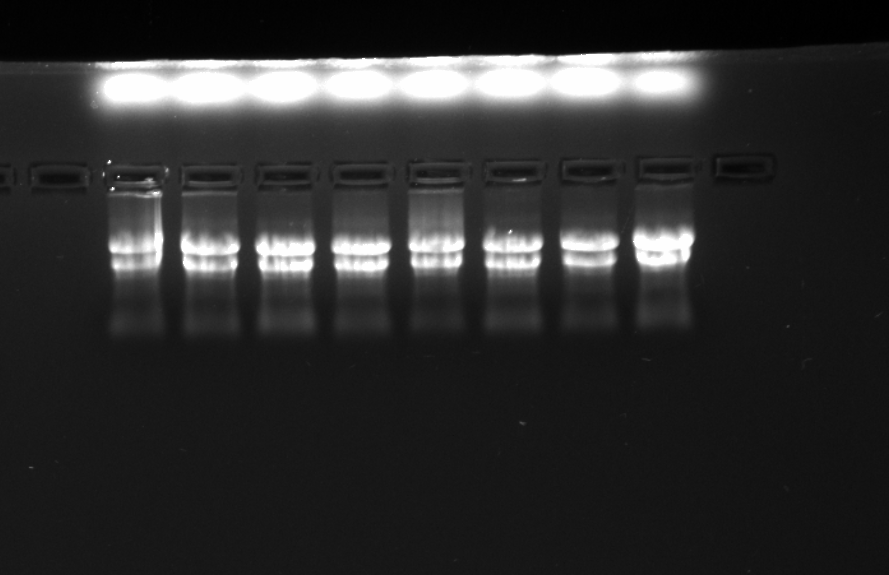

Supplement: Supplementary file 6 — Source Data for Figure 5 [file EMBR-23-e53400-s003.zip › Figure 5/5A/HMW-NB EtBr.tif]

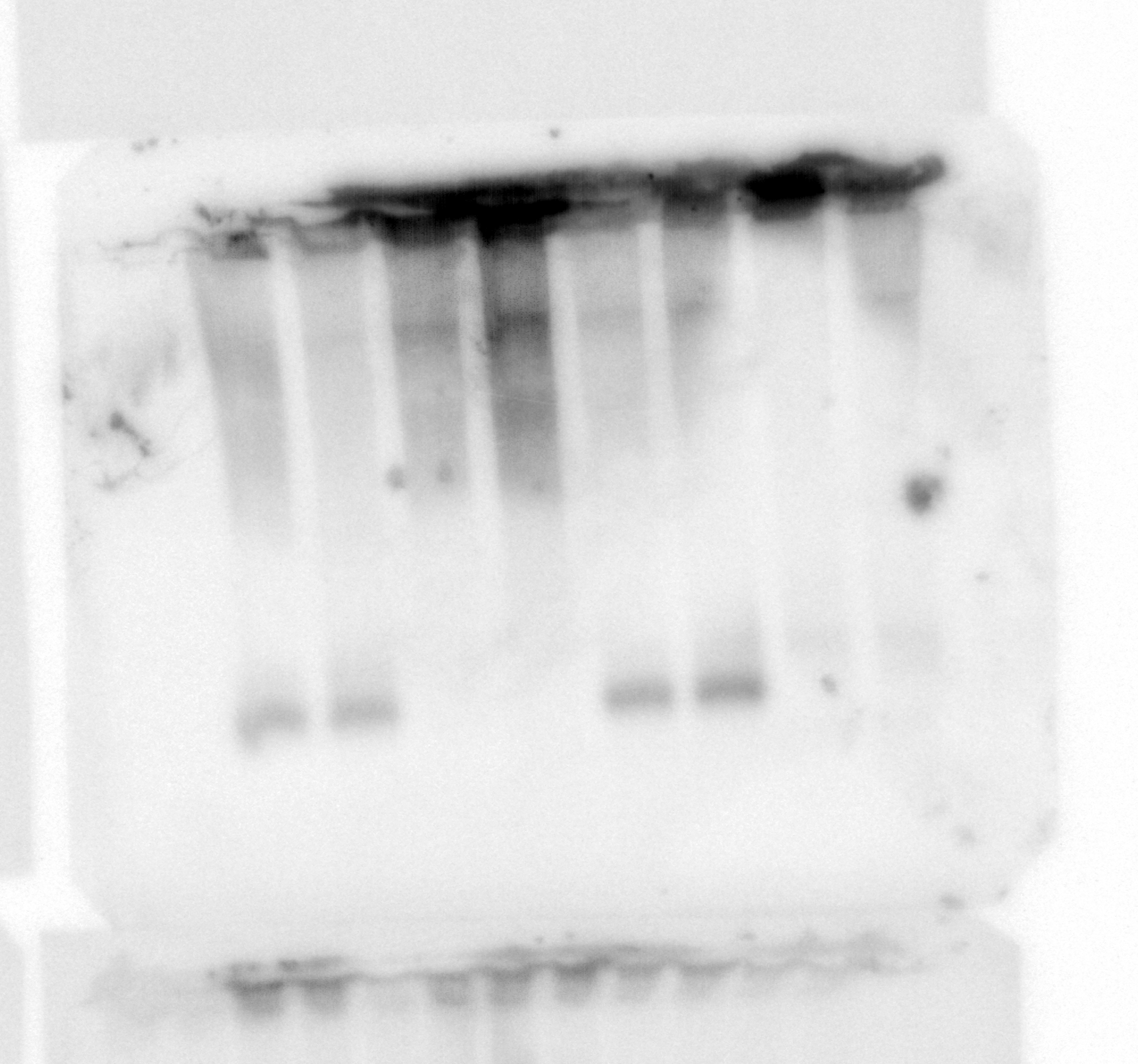

Supplement: Supplementary file 6 — Source Data for Figure 5 [file EMBR-23-e53400-s003.zip › Figure 5/5A/LMW-NB @ GFP.tif]

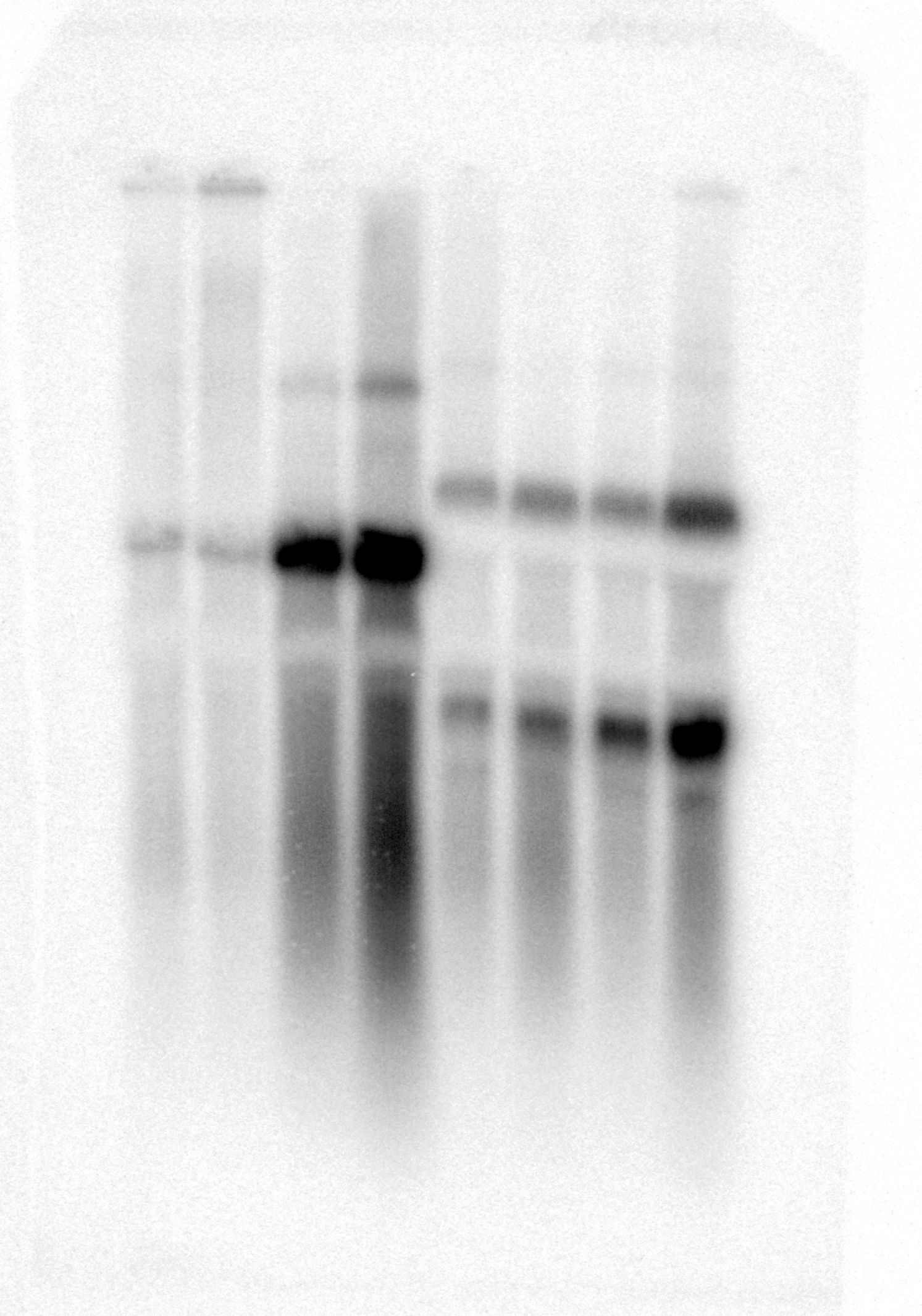

Supplement: Supplementary file 6 — Source Data for Figure 5 [file EMBR-23-e53400-s003.zip › Figure 5/5A/HMW-NB@GFP.tif]

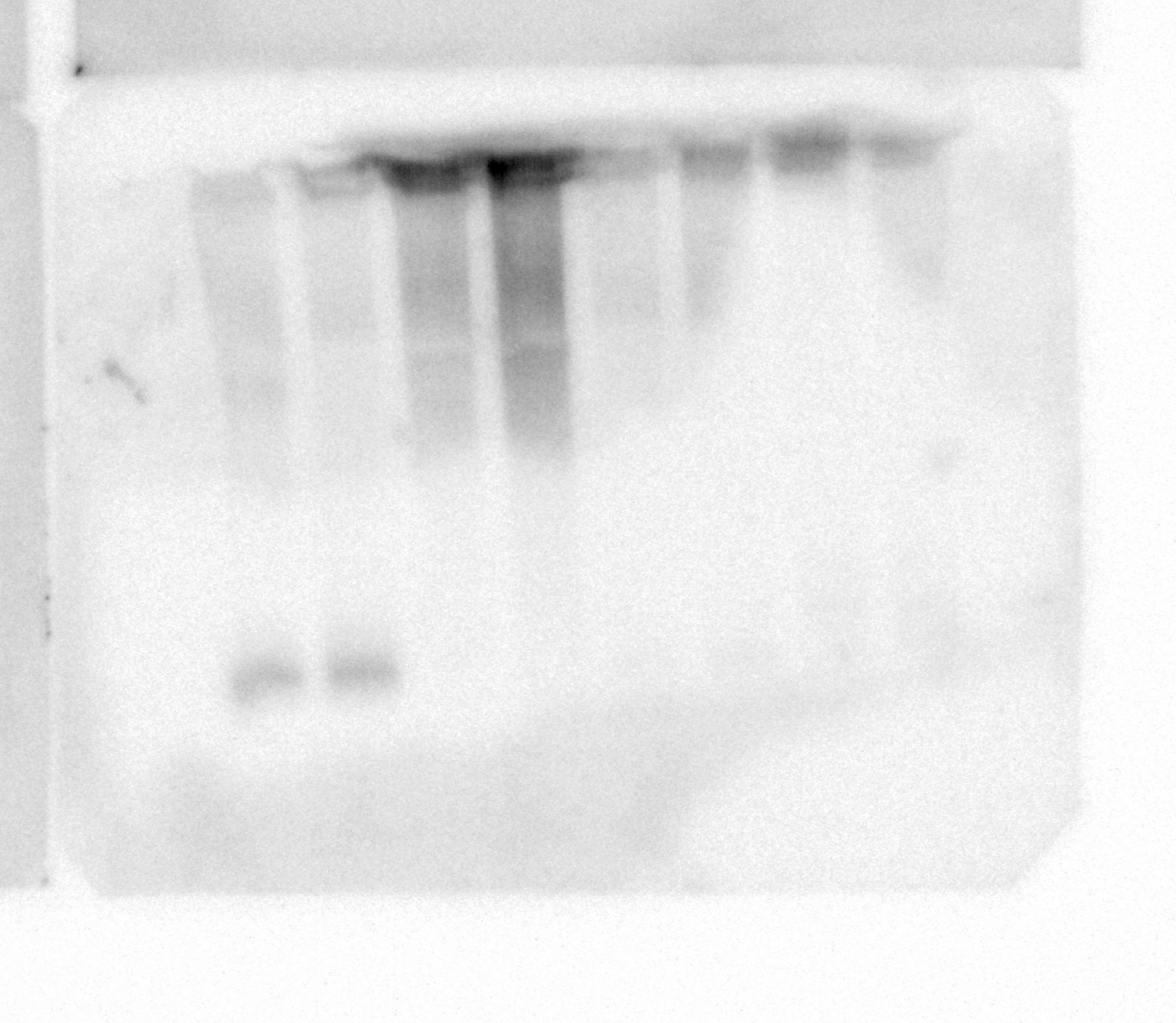

Supplement: Supplementary file 6 — Source Data for Figure 5 [file EMBR-23-e53400-s003.zip › Figure 5/5A/LMW-NB @ GUS.tif]

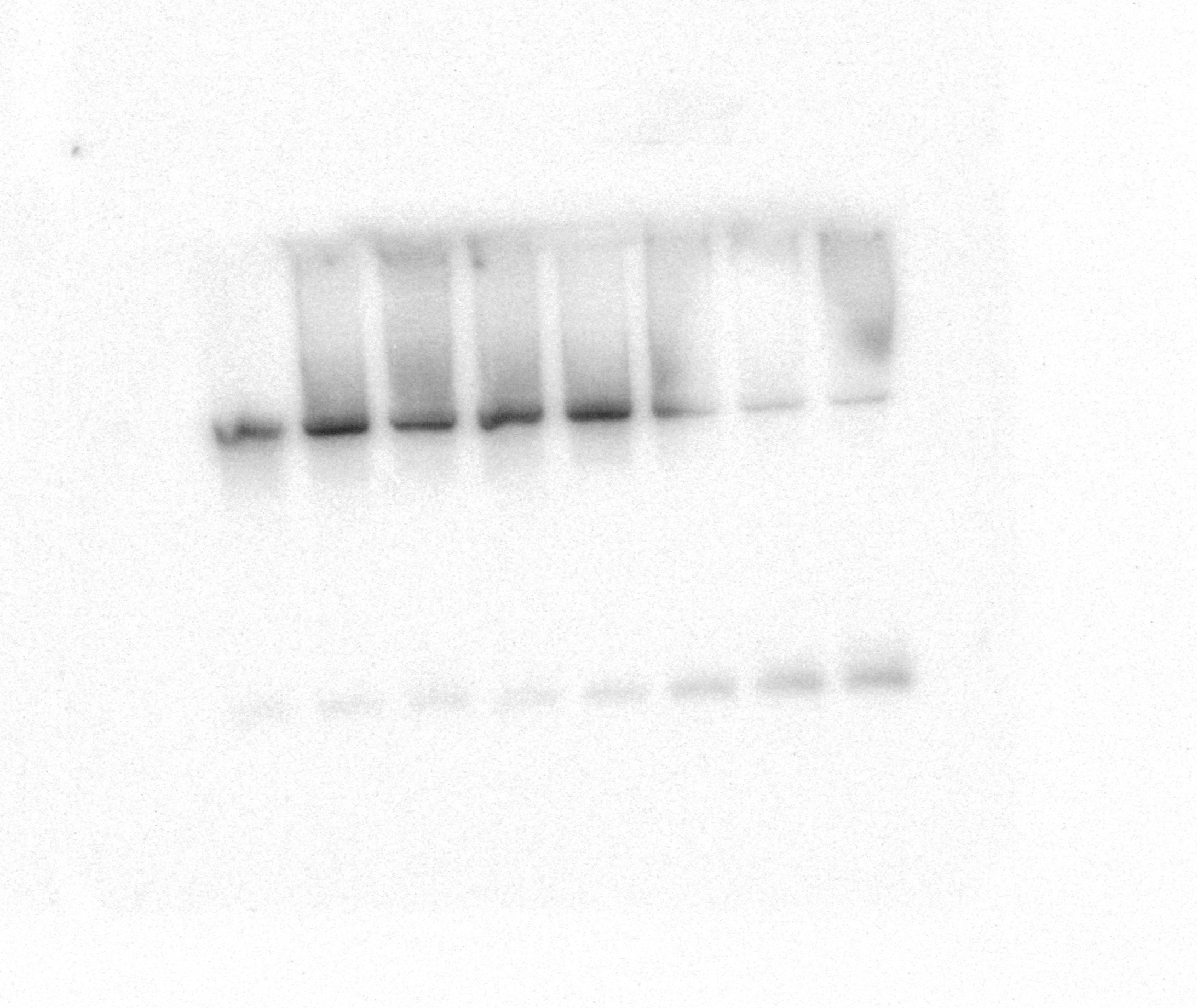

Supplement: Supplementary file 6 — Source Data for Figure 5 [file EMBR-23-e53400-s003.zip › Figure 5/5A/LMW-NB @ miR171.tif]

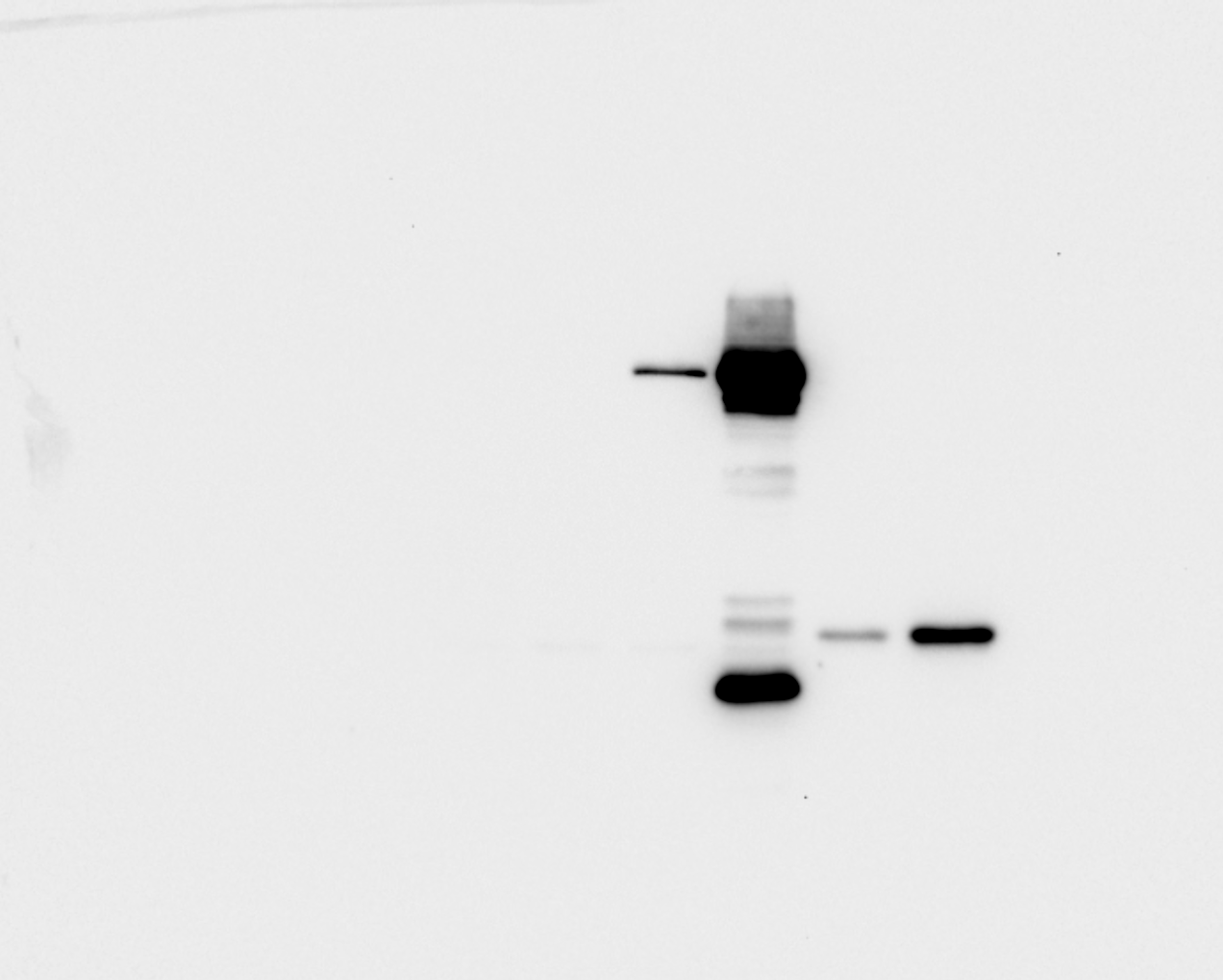

Supplement: Supplementary file 6 — Source Data for Figure 5 [file EMBR-23-e53400-s003.zip › Figure 5/5B/WB @ GAG.tif]

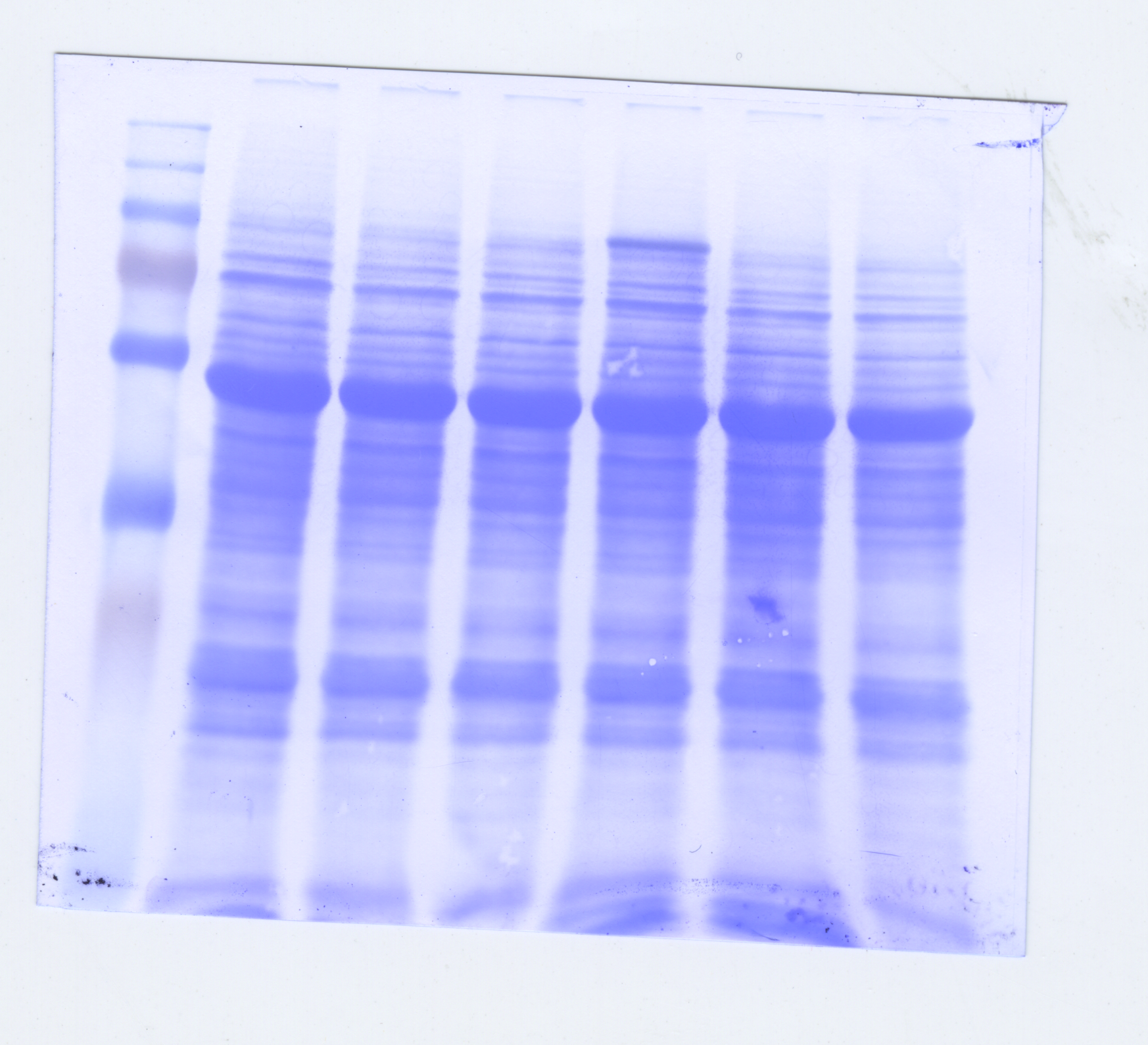

Supplement: Supplementary file 6 — Source Data for Figure 5 [file EMBR-23-e53400-s003.zip › Figure 5/5B/GAG WB Coom.tiff]
