# Supplementary material for: Innate, translation‐dependent silencing of an invasive transposon in Arabidopsis
Source: EMBO Rep. 2021 Dec 21;23(3):e53400. doi: 10.15252/embr.202153400 (PMC8892269; doi:10.15252/embr.202153400)
Supplement: Supplementary file 6 — Source Data for Figure 5 [file EMBR-23-e53400-s003.zip › Figure 5/5D/PlatePlanIII_EVD-GEG-rdr6_PolyAcDNA.pdf]

Plate III: EVD, rdr6, GEG PolyA cDNA

Plate plan

	1	2	3	4	5	6	7	8	9	10	11	12	13	14	15	16	17	18	19	20	21	22	23	24
A	A1	B1	C1	A1	B1	C1	A1	B1	C1	A1	B1	C1	A1	C1	A1	C1	B1	C1	B1	C1	A9	A9	A9	B9
B	A1	B1	C1	A1	B1	C1	A1	B1	C1	A1	B1	C1	A1	C1	A1	C1	B1	C1	B1	C1	A9	A9	A9	B9
C	A2	B2	C2	A2	B2	C2	A2	B2	C2	A2	B2	C2	A2	C2	A2	C2	B2	C2	B2	C2	B9	B9	C9	C9
D	A2	B2	C2	A2	B2	C2	A2	B2	C2	A2	B2	C2	A2	C2	A2	C2	B2	C2	B2	C2	B9	B9	C9	C9
E	A3	B3	C3	A3	B3	C3	A3	B3	C3	A3	B3	C3	A3	C3	A3	C3	B3	C3	B3	C3	C9	C9	water	water
F	A3	B3	C3	A3	B3	C3	A3	B3	C3	A3	B3	C3	A3	C3	A3	C3	B3	C3	B3	C3	C9	C9	water	water
G	A4	B4	C4	A4	B4	C4	A4	B4	C4	A4	B4	C4	A4	C4	A4	C4	B4	C4	B4	C4	water	water	A9	B9
H	A4	B4	C4	A4	B4	C4	A4	B4	C4	A4	B4	C4	A4	C4	A4	C4	B4	C4	B4	C4	water	water	A9	B9
I	A5	B5	C5	A5	B5	C5	A5	B5	C5	A5	B5	C5	A5	C5	A5	C5	B5	C5	B5	C5	A9	A9	C9	C9
J	A5	B5	C5	A5	B5	C5	A5	B5	C5	A5	B5	C5	A5	C5	A5	C5	B5	C5	B5	C5	A9	A9	C9	C9
K	A6	B6	C6	A6	B6	C6	A6	B6	C6	A6	B6	C6	A6	C6	A6	C6	B6	C6	B6	C6	B9	B9	water	water
L	A6	B6	C6	A6	B6	C6	A6	B6	C6	A6	B6	C6	A6	C6	A6	C6	B6	C6	B6	C6	B9	B9	water	water
M	A7	B7	C7	A7	B7	C7	A7	B7	C7	A7	B7	C7	A7	C7	A7	C7	B7	C7	B7	C7	C9	C9		
N	A7	B7	C7	A7	B7	C7	A7	B7	C7	A7	B7	C7	A7	C7	A7	C7	B7	C7	B7	C7	C9	C9		
O	A8	B8	C8	A8	B8	C8	A8	B8	C8	A8	B8	C8	A8	C8	A8	C8	B8	C8	B8	C8	water	water		
P	A8	B8	C8	A8	B8	C8	A8	B8	C8	A8	B8	C8	A8	C8	A8	C8	B8	C8	B8	C8	water	water		

Primers

- Actin total
- Actin unspliced
- Amp (spike)-1
- GFP-intron
- EVD spliced
- EVD unspliced
- GFP Junction
- no RT (control)

Name	Sample name	Background	BioRep	Fraction
A1	VcD0121A1	EVD_Total1	1	Total
A2	VcD0121A2	EVD_Total2	2	Total
A3	VcD0121A3	EVD_Total3	3	Total
A4	VcD0121A4	EVD_Cyto1	1	Cytoplasm
A5	VcD0121A5	EVD_Cyto2	2	Cytoplasm
A6	VcD0121A6	EVD_Cyto3	3	Cytoplasm
A7	VcD0121A7	EVD_Nuc1	1	Nuclear
A8	VcD0121A8	EVD_Nuc2	2	Nuclear
A9	VcD0121A9	EVD_Nuc3	3	Nuclear
B1	VcD0121B1	GEG_Total1	1	Total
B2	VcD0121B2	GEG_Total2	2	Total
B3	VcD0121B3	GEG_Total3	3	Total
B4	VcD0121B4	GEG_Cyto1	1	Cytoplasm
B5	VcD0121B5	GEG_Cyto2	2	Cytoplasm
B6	VcD0121B6	GEG_Cyto3	3	Cytoplasm
B7	VcD0121B7	GEG_Nuc1	1	Nuclear
B8	VcD0121B8	GEG_Nuc2	2	Nuclear
B9	VcD0121B9	GEG_Nuc3	3	Nuclear
C1	VcD0121C1	rdr6_Total1	1	Total
C2	VcD0121C2	rdr6_Total2	2	Total
C3	VcD0121C3	rdr6_Total3	3	Total
C4	VcD0121C4	rdr6_Cyto1	1	Cytoplasm
C5	VcD0121C5	rdr6_Cyto2	2	Cytoplasm
C6	VcD0121C6	rdr6_Cyto3	3	Cytoplasm
C7	VcD0121C7	rdr6_Nuc1	1	Nuclear
C8	VcD0121C8	rdr6_Nuc2	2	Nuclear
C9	VcD0121C9	rdr6_Nuc3	3	Nuclear
