# Supplementary material for: Innate, translation‐dependent silencing of an invasive transposon in Arabidopsis
Source: EMBO Rep. 2021 Dec 21;23(3):e53400. doi: 10.15252/embr.202153400 (PMC8892269; doi:10.15252/embr.202153400)
Supplement: Supplementary file 8 [file EMBR-23-e53400-s004.pdf]

EMBO reports

Innate, translation-dependent silencing of an invasive transposon in Arabidopsis

Stefan Oberlin, Rajendran Rajeswaran, Marieke Trasser, Verónica Barragán-Borrero, Michael Schon, Alexsandra Plotnikova, Lukas Loncsek, Michael Nodine, Arturo Marí-Ordóñez, and Olivier Voinnet
DOI: 10.15252/embr.202153400

Corresponding author(s): Olivier Voinnet (voinneto@ethz.ch) , Arturo Marí-Ordóñez (arturo.mari-ordonez@gmi.oeaw.ac.at)

Review Timeline:

Submission Date:	7th Jun 21
Editorial Decision:	15th Jul 21
Revision Received:	15th Oct 21
Editorial Decision:	1st Dec 21
Revision Received:	5th Dec 21
Accepted:	6th Dec 21

Editor: Esther Schnapp

Transaction Report:

(Note: With the exception of the correction of typographical or spelling errors that could be a source of ambiguity, letters and reports are not edited. Depending on transfer agreements, referee reports obtained elsewhere may or may not be included in this compilation. Referee reports are anonymous unless the Referee chooses to sign their reports.)

1st Editorial Decision 15th Jul 2021

Dear Olivier,

Thank you for the transfer of your manuscript to EMBO reports and the friendly video chat about the revisions. I paste again the referee reports below.

As we discussed, please proceed with the revisions and address all referee concerns in a detailed point-by-point response. It will be important that the main body of the manuscript is concise and clear for the general reader, and that you place your work in the context of recent related findings.

Acceptance of the manuscript will depend on a positive outcome of a second round of review. It is EMBO reports policy to allow a single round of major revision only and acceptance or rejection of the manuscript will therefore depend on the completeness of your responses included in the next, final version of the manuscript.

Revised manuscripts should be submitted within three months of a request for revision; they will otherwise be treated as new submissions. Please contact us if a 3-months time frame is not sufficient for the revisions so that we can discuss this further. You can either publish the study as a short report or as a full article. For short reports, the revised manuscript should not exceed 27,000 characters (including spaces but excluding materials & methods and references) and 5 main plus 5 expanded view figures. The results and discussion sections must further be combined, which will help to shorten the manuscript text by eliminating some redundancy that is inevitable when discussing the same experiments twice. For a normal article there are no length limitations, but it should have more than 5 main figures and the results and discussion sections must be separate. In both cases, the entire materials and methods should be included in the main manuscript file.

Regarding data quantification, please specify the number "n" for how many independent experiments were performed, the bars and error bars (e.g. SEM, SD) and the test used to calculate p-values in the respective figure legends. This information must be provided in the figure legends. Please also include scale bars in all microscopy images.

IMPORTANT NOTE: we perform an initial quality control of all revised manuscripts before re-review. Your manuscript will FAIL this control and the handling will be DELAYED if the following APPLIES:
1) A data availability section providing access to data deposited in public databases is missing. If you have not deposited any data, please add a sentence to the data availability section that explains that.
2) Your manuscript contains statistics and error bars based on n=2. Please use scatter blots in these cases. No statistics should be calculated if n=2.

When submitting your revised manuscript, please carefully review the instructions that follow below. Failure to include requested items will delay the evaluation of your revision.

1) a .docx formatted version of the manuscript text (including legends for main figures, EV figures and tables). Please make sure that the changes are highlighted to be clearly visible.

2) individual production quality figure files as .eps, .tif, .jpg (one file per figure).
See https://wol-prod-cdn.literatumonline.com/pb-assets/embo-site/EMBOPress_Figure_Guidelines_061115-1561436025777.pdf for more info on how to prepare your figures.

3) We replaced Supplementary Information with Expanded View (EV) Figures and Tables that are collapsible/expandable online. A maximum of 5 EV Figures can be typeset. EV Figures should be cited as 'Figure EV1, Figure EV2" etc... in the text and their respective legends should be included in the main text after the legends of regular figures.

- For the figures that you do NOT wish to display as Expanded View figures, they should be bundled together with their legends in a single PDF file called *Appendix*, which should start with a short Table of Content. Appendix figures should be referred to in the main text as: "Appendix Figure S1, Appendix Figure S2" etc. See detailed instructions regarding expanded view here: <https://www.embopress.org/page/journal/14693178/authorguide#expandedview>

- Additional Tables/Datasets should be labeled and referred to as Table EV1, Dataset EV1, etc. Legends have to be provided in a separate tab in case of .xls files. Alternatively, the legend can be supplied as a separate text file (README) and zipped together with the Table/Dataset file.

4) a .docx formatted letter INCLUDING the reviewers' reports and your detailed point-by-point responses to their comments. As part of the EMBO Press transparent editorial process, the point-by-point response is part of the Review Process File (RPF), which will be published alongside your paper.

5) a complete author checklist, which you can download from our author guidelines <https://www.embopress.org/page/journal/14693178/authorguide>. Please insert information in the checklist that is also

reflected in the manuscript. The completed author checklist will also be part of the RPF.

6) Please note that all corresponding authors are required to supply an ORCID ID for their name upon submission of a revised manuscript (<https://orcid.org/>). Please find instructions on how to link your ORCID ID to your account in our manuscript tracking system in our Author guidelines
<https://www.embopress.org/page/journal/14693178/authorguide#authorshipguidelines>

7) Before submitting your revision, primary datasets produced in this study need to be deposited in an appropriate public database (see https://www.embopress.org/page/journal/14693178/authorguide#datadeposition). Please remember to provide a reviewer password if the datasets are not yet public. The accession numbers and database should be listed in a formal "Data Availability" section placed after Materials & Method (see also https://www.embopress.org/page/journal/14693178/authorguide#datadeposition). Please note that the Data Availability Section is restricted to new primary data that are part of this study. * Note - All links should resolve to a page where the data can be accessed. *
If your study has not produced novel datasets, please mention this fact in the Data Availability Section.

8) We would also encourage you to include the source data for figure panels that show essential data. Numerical data should be provided as individual .xls or .csv files (including a tab describing the data). For blots or microscopy, uncropped images should be submitted (using a zip archive if multiple images need to be supplied for one panel). Additional information on source data and instruction on how to label the files are available at
<https://www.embopress.org/page/journal/14693178/authorguide#sourcedata>.

9) Our journal also encourages inclusion of *data citations in the reference list* to directly cite datasets that were re-used and obtained from public databases. Data citations in the article text are distinct from normal bibliographical citations and should directly link to the database records from which the data can be accessed. In the main text, data citations are formatted as follows: "Data ref: Smith et al, 2001" or "Data ref: NCBI Sequence Read Archive PRJNA342805, 2017". In the Reference list, data citations must be labeled with "[DATASET]". A data reference must provide the database name, accession number/identifiers and a resolvable link to the landing page from which the data can be accessed at the end of the reference. Further instructions are available at https://www.embopress.org/page/journal/14693178/authorguide#referencesformat

We would also welcome the submission of cover suggestions, or motifs to be used by our Graphics Illustrator in designing a cover.

As part of the EMBO publication's Transparent Editorial Process, EMBO reports publishes online a Review Process File (RPF) to accompany accepted manuscripts. This File will be published in conjunction with your paper and will include the referee reports, your point-by-point response and all pertinent correspondence relating to the manuscript.

You are able to opt out of this by letting the editorial office know (emboreports@embo.org). If you do opt out, the Review Process File link will point to the following statement: "No Review Process File is available with this article, as the authors have chosen not to make the review process public in this case."

I look forward to seeing a revised version of your manuscript when it is ready. Please let me know if you have questions or comments regarding the revision.

Kind regards,
Esther

Esther Schnapp, PhD
Senior Editor
EMBO reports

Referee #1:

In this ms, the authors analyze how reactivated copies of the Arabidopsis transposable element (TE) EVD (a Ty/Copia-like retrotransposon) are targeted for resilencing. They show that a splicing-coupled premature cleavage and polyadenylation event provokes the production of a short mRNA that is efficiently exported in the cytoplasm. This short mRNA is translated but an expected and unexplained, discrete and intense, ribosome stalling causes the cleavage of the mRNA and the liberation of a 3' fragment bearing a 5'OH end that escapes degradation by the exoribonuclease XRN4 (which activity requires a 5'P). As a consequence, this fragment is efficiently transformed into double-stranded RNA by the cellular RNA-dependent RNA polymerase RDR6 to initiate silencing (explaining why siRNAs come exclusively from this fragment and not from the full EVD RNA). Importantly, they recapitulate these findings by introducing the splicing-coupled premature cleavage and polyadenylation site in a reporter transgene.

The amount of work is impressive. The data are solid and support the conclusions of the paper. Coincidently, a paper that is almost theorical was recently published, hypothesizing that suboptimal codon usage and low GC content were causing inefficient translation, ribosome stalling and RNA cleavage to initiate TE silencing (Kim et al, 2021). At least in the case of EVD, this theory seems wrong, which makes this very paper important.

Three big questions remain: 1) what is the determinant that causes ribosome stalling? 2) How does RNA cleavage occur? 3) How general is the model that apply to EVD? I don't expect the authors to address the first two questions in this paper. However, I think it is important that they elaborate on the third question. At first, the ms is written as if EVD was the only model of TE copy number-dependent de novo resilencing. For example, the authors ignore work done with the tobacco Tnt1 element (another Copia-like retrotransposon), which becomes silenced in a copy number-dependent manner when introduced in Arabidopsis (Perez-Hormaeche et al, 2008). I am not expecting the authors to look for Tnt1 RNA fragments bearing a 5'OH end either in Arabidopsis transgenics or in tobacco protoplasts where the expression of Tnt1 is induced, but at least they should discuss this paper. In addition, the authors propose that this model also applies to transgenes that frequently undergo silencing. Did they found ribosome stalling or transgene RNA fragments bearing a 5'OH in silenced transgenic plants ?

Minor comment: there is a problem to fix between Figures S9 and S10.

Referee #2:

In this manuscript, the authors aimed to understand how transposons induce PTGS in plants. They rejected several previously proposed mechanisms of PTGS (e.g., saturation of the mRNA decay pathway), based on the failure to detect apparent correlations between their occurrence and siRNA production. On other hand, they observed that ribosome stalling and production of 5'-OH RNA fragments correlate with RDR6-dependent siRNA accumulation, which led them to propose "a hitherto-unrecognized" translation-dependent silencing mechanism (TdS). Although the connection between ribosome stalling and siRNA production has already been reported (Kim et al., Nature Plants 2021; Iwakawa et al., BioRxiv 2020), this study has an interesting point in that they found the presence of 5'OH RNA fragments that coincide with ribosome stalling sites. However, the current manuscript merely describes apparent correlations, and the proposed model lacks direct evidence.

Major Points:
1. The biggest problem of this manuscript is that the causal relationship (or perhaps the absence of the direct link) between 5'OH RNAs and PTGS remains unexplored. The authors should block translation of shGAG by introducing a stable stem loop(s) or a stop codon(s) and check the production and 5'OH RNAs and siRNAs.
2. The authors concluded that shGAG siRNA production is miRNA-independent by using dcl1 and hyl1 mutants. However, it is still possible that a background level of siRNAs, which are produced from single-stranded EVD RNAs independently of RDR6 (Fig 1B), prime the production of abundant shGAG siRNAs by recruiting RDR6 to the EVD RNAs. A recent preprint showed that ribosome stalling is a positive modulator, but not a trigger of secondary siRNA (tasiRNA) biogenesis, which is initiated by RISC binding (Iwakawa et al., bioRxiv 2020). shGAG siRNAs might also be this type of secondary siRNAs, whose production is triggered by siRNA-RISC binding and enhanced by ribosome stalling. 5' OH RNAs might just be a byproduct of RISC-mediated ribosome stalling. To rule out this possibility, the anti-sense strand of EVD RNA should be detected in dcl2/3/4 and ago1-27 mutants. Otherwise, the conclusion that 5' OH RNAs "trigger" shGAG siRNA production should be significantly weakened and the above discussion should be included.
3. Fig. 5A demonstrates strong accumulation of siRNAs even from 35S:GFP-GUS, which lacks any elements of EVD or other transposons. Do the authors detect ribosome stalling and corresponding 5'OH RNAs upstream of the siRNA production site on 35S:GFP-GUS?
4. The authors often use "RDR6 recruitment" and "PTGS initiation", but in fact they do not discriminate between synthesis of the anti-sense strand and amplification of secondary siRNAs. If they wish to use these terms, they should detect the anti-sense RNAs in dcl2/3/4 mutant background (as in Rajeswaran et al., NAR 2012).

Referee #3:

This is the latest in a series of interesting and probing papers from Mari-Ordonez and colleagues who have used the EVADE transposon to understand how a host may control transposons. I congratulate them on what seems to be a large body of work that may bring translation into the sphere of TE control. I understand the individual experiments but must confess that I am still a little fuzzy about some parts (outlined below). Its entirely possible that the pandemic has dulled my critical thinking and I am missing something here and any clarifications the authors provide would be greatly helpful.
Overall, I feel that this study provides many valuable observations and definitely deserves to be published. I dislike having to ask for more experiments but have some comments outlined below that I feel may make this paper better.
Major issues
1. Can the authors better explain the results and the conclusions drawn from Figure 3. It is commendable that the authors did the experiment of both inserting an intron and removing the intron or splicing. While these results are interesting, they are hard

to understand. What is the difference between "trigger" and "initiate"? You say its splicing coupled PCPA (for example in line 245 on page 12) but you can get rid of splicing and still have the sRNA production.

2. The authors suggest that translation may be necessary for recruitment of RDR6. Is the mU1 version of shGAG, which makes no proteins but still makes sRNAs, associated with polysomes?

3. The authors have shown that FL GAG-Pol is expressed at much higher levels (~9x more in WT?) than shGAG. How does this factor into interpretation of the role of nuclear sequestration of FL-GAG in differential translation? A back of the envelope calculation suggests that there is still more FL-GAG in the cytoplasm then there is shGAG. To me, that suggests that there are perhaps other mechanisms that also contribute to the differential translation.

4. The manuscript cites studies in which splicing promoters nuclear export. Are the mU1/intron deleted GAG exported efficiently to the cytoplasm?

5. The ribosome stalling data looks solid and perhaps could be the end of the paper.
The conclusions that can be drawn from observations of ribothrypsis are vague and makes it difficult for the reader to wrap their head around the result. For example, Fig S11a suggests that the transcripts that are breaking don't have poly(A) tails. However, on Page 10, lines 188-190, the authors conclude that polyadenylation defects don't contribute to the PTGS. Its ofcourse possible that small RNAs are being produced from a small subset of non-polyadenylated shGAG
Minor Issues
1. I would suggest to the authors to add all gels(in their whole and unspliced form to supplements).

2. Figure S3 is hard to interpret/understand. In S3a, what are the two bars for each genotype? What are the error bars? Were technical replicates done for the qPCR? Also, in the interest of easy to understand figure design, it might be useful if the "-" in poly (A) is superscripted and % of input is shifted over to the next line. I also suggest that the authors show both duplicates as separate graphs instead of clubbing them and calculating standard error. I am not sure its good practice to calculate standard error for N=2.
3. Fig S3a suggests that there was low rRNA in the non-Poly(a) fraction in rdr6 relative to wild-type . Does this represent something biological variation or is it technical variation? If its technical variation, is the difference so huge that it suggests some sort of failure to isolate the non-PolyA fraction in rdr6 and does this mean that you cant draw meaningful conclusions about the shgag levels in non-poly(A)? Is it better instead to measure tail length of shgag in wild-type and rdr6? If your model is right, then tail length should be similar between wt and rdr6. However, if non poly(A) shgag is increased in levels, then tail length can reduce.

4. In Figure 2, it might be useful to indicate the position of the PCPA signal.

5. In Figure S4B, the fraction of small RNAs that are intron spanning (i.e exon-exon) vs ones that are exon-intron might be important. This was also missing in Oberlin et al (2017). Its unclear if its just a handful of junction spanning reads that are found or its actually quite a large number and represent something meaningful.

6. Overall, I feel like the manuscript can be greatly improved by reorganizing the paper a bit and by proof reading (for example, I suspect that Page10, line 188 may be missing "out" from " We ruled that ....") and better sentence construction. It's a complicated topic and better organization and language will make this manuscript a more pleasurable read. In other examples, can you change the colors in Fig3D? The splice and unspliced GAG are colored using a scheme which makes it hard for some people to distinguish. See also heading on line 351, page 16. It probably should read "unlikely to trigger".

Point-by-point COMMENTS TO REVIEWERS:

Referee 1:

In this ms, the authors analyze how reactivated copies of the Arabidopsis transposable element (TE) EVD (a Ty/Copia-like retrotransposon) are targeted for resilencing. They show that a splicing-coupled premature cleavage and polyadenylation event provokes the production of a short mRNA that is efficiently exported in the cytoplasm. This short mRNA is translated but an expected and unexplained, discrete and intense, ribosome stalling causes the cleavage of the mRNA and the liberation of a 3' fragment bearing a 5'OH end that escapes degradation by the exoribonuclease XRN4 (which activity requires a 5'P). As a consequence, this fragment is efficiently transformed into double-stranded RNA by the cellular RNA-dependent RNA polymerase RDR6 to initiate silencing (explaining why siRNAs come exclusively from this fragment and not from the full EVD RNA). Importantly, they recapitulate these findings by introducing the splicing-coupled premature cleavage and polyadenylation site in a reporter transgene.

The amount of work is impressive. The data are solid and support the conclusions of the paper. Coincidently, a paper that is almost theorical was recently published, hypothesizing that suboptimal codon usage and low GC content were causing inefficient translation, ribosome stalling and RNA cleavage to initiate TE silencing (Kim et al, 2021). At least in the case of EVD, this theory seems wrong, which makes this very paper important.

Three big questions remain:
1) what is the determinant that causes ribosome stalling?
2) How does RNA cleavage occur?
3) How general is the model that apply to EVD?

Since receiving the referees' reports we did look at the possible causes of stalling by either replacing the suspected Proline-Glycine-associated main stalling site detected in *EVD* or deleting a strong G-quadruplex-promoting sequence located just upstream of the main stalling site detected in the *GFP-EVD$_{in/ter}$-GUS* reporter. These new experiments are shown in a new version of Supplemental Information 10, but they did not yield the expected results, with siRNAs being equally generated from both original and modified versions of the constructs in bulked T1 analyses. Thus, this question, just like question 2 (we are also working on it) remains to be addressed by future work, as conceded by the referee.

I don't expect the authors to address the first two questions in this paper. However, I think it is important that they elaborate on the third question. At first, the ms is written as if EVD was the only model of TE copy number-dependent de novo resilencing. For example, the authors ignore work done with the tobacco Tnt1 element (another Copia-like retrotransposon), which becomes silenced in a copy number-dependent manner when introduced in Arabidopsis (Perez-Hormaeche et al, 2008). I am not expecting the authors to look for Tnt1 RNA fragments bearing a 5'OH end either in Arabidopsis transgenics or in tobacco protoplasts where the expression of Tnt1 is induced, but at least they should discuss this paper. In addition, the authors propose that this model also applies to transgenes that frequently undergo silencing. Did they found ribosome stalling or transgene RNA fragments bearing a 5'OH in silenced transgenic plants ?

In response to the referee's comment, we have removed the speculation on TdS being a source of PTGS from transgenes in the very end of the discussion, because we did not check this in details beyond mere correlations we could establish on some, but not other, transgenes between initiation of siRNA production and suboptimal codons. We have added, instead, the relevant discussion on *Tnt1* (and, as we found, also *Tto1*) on lines 645-654. In fact, we were already citing the work from 2000 but not explicitly emphasizing the results.

Minor comment: there is a problem to fix between Figures S9 and S10.
These errors have now been rectified.

Referee 2:

In this manuscript, the authors aimed to understand how transposons induce PTGS in plants. They rejected several previously proposed mechanisms of PTGS (e.g., saturation of the mRNA decay pathway), based on the failure to detect apparent correlations between their occurrence and siRNA production. On other hand, they observed that ribosome stalling and production of 5'-OH RNA fragments correlate with RDR6-dependent siRNA accumulation, which led them to propose "a hitherto-unrecognized" translation-dependent silencing mechanism (TdS). Although the connection between ribosome stalling and siRNA production has already been reported (Kim et al., Nature Plants 2021; Iwakawa et al., BioRxiv 2020), this study has an interesting point in that they found the presence of 5'OH RNA fragments that coincide with ribosome stalling sites. However, the current manuscript merely describes apparent correlations, and the proposed model lacks direct evidence.

We fully acknowledge that, as of now, we have only gathered correlative as opposed to causal, evidence in support of our model. Hence we were very careful in the originally submitted version of the manuscript to avoid terms inferring causality and have been even more careful in the revised version (see in particular our response below to point 4) in light of the unconclusive results obtained after altering possible stalling-inducing features of *shGAG* and *shGFP* (see our response to point one, below). As pointed out in the discussion, we will further investigate this issue in the future to get insights into the mechanism and generality of the model.

Major Points:

1. The biggest problem of this manuscript is that the causal relationship (or perhaps the absence of the direct link) between 5'OH RNAs and PTGS remains unexplored. The authors should block translation of shGAG by introducing a stable stem loop(s) or a stop codon(s) and check the production and 5'OH RNAs and siRNAs.

As *shGAG* is already undergoing substantial ribosome stalling, we have now conducted the opposite experiment by introducing mutations at the Proline-Glycine-codons where the main stalling occurs, in order to reduce/abolish the process and, if our model is correct, siRNA biogenesis. In parallel, we had noticed a G-quadruplex-promoting region located just upstream of the main stalling event seen on *shGFP* from the *GFP-EVD*$_{in/ter}$*-GUS* reporter, which we deleted. All these new experiments are now depicted in a new version of Supplemental Information 10, but they did not yield the expected results, with siRNAs being equally generated from both original and modified versions of the constructs in bulked T1 analyses. Thus, the origins of ribosome stalling remain unknown, as is therefore its causality in 5’OH RNA generation and RDR6 stimulation. We have now recognized this in full, on lines 651-658 of the revised manuscript : “*… leading us to consider a potential causal role for stalling-coupled 5’-OH RNA fragment generation in stimulating RDR6 activity. Since we were unable, however, to genetically impede stalling (Figure SX), we can only speculate in the model described in the next section and emphasize that, at this stage, 5’OH RNA fragments might be mere byproducts of ribosome stalling.*

2. The authors concluded that shGAG siRNA production is miRNA-independent by using dcl1 and hyl1 mutants. However, it is still possible that a background level of siRNAs, which are produced from single-stranded EVD RNAs independently of RDR6 (Fig 1B), prime the production of abundant shGAG siRNAs by recruiting RDR6 to the EVD RNAs. A recent preprint showed that ribosome stalling is a positive modulator, but not a trigger of secondary siRNA (tasiRNA) biogenesis, which is initiated by RISC binding (Iwakawa et al., bioRxiv 2020). shGAG siRNAs might also be this type of secondary siRNAs, whose production is triggered by siRNA-RISC binding and enhanced by ribosome stalling. 5' OH RNAs might just be a byproduct of RISC-mediated ribosome stalling. To rule out this possibility, the anti-sense strand of EVD RNA should be detected in dcl2/3/4 and ago1-27 mutants. Otherwise, the conclusion that 5' OH RNAs "trigger" shGAG siRNA production should be significantly weakened and the above discussion should be included.

For the sake of space, the interesting alternative scenario suggested by reviewer 2 is now developed in Supplementary discussion added to Supplementary information, which is called upon, in the cognate context of the now published work of Iwakawa *et al*. (2021), on line 655 of the main text of the revised manuscript (also now cited on lines 547-549, 655-657). Briefly, we explain, in the supp. Discussion, that for these ‘primal’ sRNAs to trigger silencing, they would have to be *antisense* to the *EVD sense* RNA as well as being RDR6-independent because stalling occurs in the *rdr6* background (Figure 6B). Meticulous mining of our deep-seq data indicates that the background-level sRNAs produced in *rdr6* from both endogenous and transgenic *EVD* show no obvious enrichment downstream of the main ribosome stalling site and concerns species in the *sense*, as opposed to *antisense*, orientation (Fig.1B, Supp.Fig. 1C). Furthermore, over the coding region, this very low background is equally visible along *GAG* and *POL* regions (Suppl.Fig.13), yet only *shGAG* spawns RDR6-dependent siRNAs. We explain that these features evoke those of normal turnover products of the sense *shGAG* and *flGAG-POL* transcripts, making it therefore unlikely –albeit still hypothetically possible, that stalling is initiated in the manner suggested by reviewer 2. In light of the above observations, we did not perform the suggested genetic experiments, also because *ago1-27* (one of few hypomorphic, fertile Arabidopsis *ago1* mutants) is not compromised for si/miRNA loading, but only for slicing, which might be unnecessary for stalling according to the model of Iwakawa *et al*. (2021).

To further take into consideration reviewer 2's correct contention that our hypothesis for a role for 5'OH RNA fragments remains a mere model at this stage, we have considerably reduced what used to form the last section of the discussion on this specific topic (formally 817-, now reduced to 380-words).

3. Fig. 5A demonstrates strong accumulation of siRNAs even from 35S:GFP-GUS, which lacks any elements of EVD or other transposons. Do the authors detect ribosome stalling and corresponding 5'OH RNAs upstream of the siRNA production site on 35S:GFP-GUS?

No, we have not investigated ribosome profiling on the *GFP-GUS* transgene, which is used as a mere negative control for experiments conducted with the $GFP\text{-}EVD_{in/ter}\text{-}GUS$ construct. As explained in the main text, transgenic lines exhibiting sense-PTGS of *GFP-GUS* were selected to emphasize the key difference in the siRNA pattern generated: with *GFP-GUS* it covers the entirety of the construct, whereas it only encompasses the *shGAG*-like *shGFP* in $GFP\text{-}EVD_{in/ter}\text{-}GUS$. The trigger for siRNA production from *GFP-GUS* remains unknown to us, but it may pertain to saturation of RNA-quality control (RQC) as strongly suggested with the famous L1 S-PTGS *GUS* line from the Vaucheret group (Gy *et al*., 2007, The Plant Cell).

4. The authors often use "RDR6 recruitment" and "PTGS initiation", but in fact they do not discriminate between synthesis of the anti-sense strand and amplification of secondary siRNAs. If they wish to use these terms, they should detect the anti-sense RNAs in dcl2/3/4 mutant background (as in Rajeswaran et al., NAR 2012).

Although we were cautious not to imply causality in the first manuscript's draft, we list below all instances where a correlation is evoked in the revised manuscript, of which some have now been amended to further tone down:

- Line 13 in the **abstract**: "…during which an unusually intense ribosomal stalling event coincides precisely with the starting-point of sRNA production exclusively on shGAG. mRNA breakage occurring at this starting-point yields unconventional 5'OH RNA fragments that evade RNA-quality-control and concomitantly likely stimulate RNA-DEPENDENT-RNA-POLYMERASE-6 (RDR6) to initiate sRNA production."
  has now been replaced with:
  "During this process, an unusually intense ribosomal stalling event coincides with mRNA breakage yielding unconventional 5'OH RNA fragments that evade RNA-quality-control. The starting-point of sRNA production by RNA-DEPENDENT-RNA-POLYMERASE-6 (RDR6), exclusively on *shGAG*, occurs precisely at this breakage point."

- Line 131: "…we suggest that the 5'OH status of breakage fragments licenses their conversion into dsRNA by RDR6" has now been replaced by: "…we suggest that that the 5'OH status of breakage fragments **contributes** to their conversion into dsRNA by RDR6…".

- Line 412 "the initiation of RDR6 activity **coincides** with …". Not changed;

- Line 436: "Stalling is likely causal, not a consequence of RDR6 recruitment, because it also occurs in the *rdr6* background (Fig.7A)" is now simply rephrased as: "**Stalling is not a consequence** of RDR6 recruitment, because it also occurs in the *rdr6* background (Fig.7A)."

- Line 439:" To explore further a possible link between discrete, intense ribosome stalling and RDR6 recruitment…" has now been replaced by: "To explore further a possible link between discrete, intense ribosome stalling and RDR6 **activity**…";

- Line 482: "RDR6-dependent production of shGAG- or shGFP-only siRNAs **coincides** with highly localized and unusually intense ribosome stalling events". Not changed;

- Line 489: " …5'-hydroxy 3'-cleavage fragments that possibly serve as RDR6 substrates". Not changed;

- Line 502: " …might constitute RDR6 templates…". Not changed;

- Line 555: "This process, however, concomitantly stimulates RDR6 activity" has now been replaced by: "This process, however, concomitantly **correlates with** RDR6 activity";

- Line 558: "Rather, an exceptionally intense and highly discrete ribosome stalling event predisposes *shGAG* to RDR6-dependent PTGS" has been replaced with "Rather, an exceptionally intense and highly discrete ribosome stalling event **coincides with** RDR6-dependent PTGS of *shGAG*".

Referee 3:

This is the latest in a series of interesting and probing papers from Mari-Ordonez and colleagues who have used the EVADE transposon to understand how a host may control transposons. I congratulate them on what seems to be a large body of work that may bring translation into the sphere of TE control. I understand the individual experiments but must confess that I am still a little fuzzy about some parts (outlined below). Its entirely possible that the pandemic has dulled my critical thinking and I am missing something here and any clarifications the authors provide would be greatly helpful.

Overall, I feel that this study provides many valuable observations and definitely deserves to be published.

We thank the reviewer for this positive evaluation of our work.

I dislike having to ask for more experiments but have some comments outlined below that I feel may make this paper better.

Major issues

1. Can the authors better explain the results and the conclusions drawn from Figure 3. It is commendable that the authors did the experiment of both inserting an intron and removing the intron or splicing. While these results are interesting, they are hard to understand.

The *EVD* splicing mutant lines were fully described in our previous work (Oberlin et al., 2017) where we dissected how TE splicing mediates premature cleavage and polyadenylation (PCPA) in order to generate *shGAG*-only mRNAs. A graphical summary of the findings presented in our previous manuscript was originally added to Figure 3. We thus might have taken for granted that the reader is familiar with such a process. Figure 3 was not intended to explain or explore the role and mechanisms of *EVD* splicing as it was extensively discussed in Oberlin *et al*., but it's potential role in triggering PTGS. This was important to investigate, given that work in yeast previously showed that abnormal TE splicing can initiate RdRP-dependent siRNA biogenesis (Dumesic *et al.,* 2013, Cell). In fact, splicing does not influence siRNA production as long as the PCPA takes place to generate *shGAG* and this can happen in the absence of splicing, as long as U1 snoRNA-binding is impeded (Oberlin et al., 2017). Altogether, these experiments strongly suggest that initiation of PTGS on *EVD* is independent of the transposon's unique expression and splicing mechanism sand always using *shGAG* transcripts as substrates for RDR6 with or without splicing as concluded in lines 237-241.

What is the difference between "trigger" and "initiate"?
These words were, in fact, used equivalently in the first version of the manuscript. However, we agree with reviewer 2 that our work merely established correlations between stalling, 5'OH RNA fragments and RDR6 recruitment. They did not establish causality, for which abrogating stalling would first be required. Yet, despite intense work during the revisions (now presented in Supplementary Figure 10) we failed to identify the cause for stalling on *shGAG* and *shGFP*. This has led us to be more prudent in the wording of the revised version, where we have avoided words such as "trigger", or "initiate", now replaced with "coincide" or more indirect wording. Establishing causality will await further experiments and we may be more lucky by taking the cleavage approach because we now have a credible candidate for the nuclease involved, but it is still very preliminary.

You say its splicing coupled PCPA (for example in line 245 on page 12) but you can get rid of splicing and still have the sRNA production.
This point was developed extensively in Oberlin *et al*., 2017. One of the roles of U1 snoRNA is to inhibit premature termination and polyadenylation at AT-rich intron sequences. Preventing splicing by mutating U1 binding site leads to the generation of both *flGAG-POL* and *shGAG* unspliced, as U1 can't prevent anymore the recognition of the proximal PAS as explained in lines 224-230. Indeed, the unspliced *shGAG* mRNA still produces siRNAs. In the absence of splicing this *shGAG* transcript lacks stop codon, probably triggering additional no-stop RNA decay (lines 230-234), see also reply to next comment.

2. The authors suggest that translation may be necessary for recruitment of RDR6. Is the mU1 version of shGAG, which makes no proteins but still makes sRNAs, associated with polysomes?
Although we have not performed polysome pull-down experiments in such lines, our experiments show that:

- *ShGAG* is indeed produced in the absence of splicing from the *EVD mU1* construct (Figure 3C,E);
- Lack-of-splicing generates a *shGAG* mRNA lacking a stop codon (Figure 3C, Oberlin et al 2017);
- Intron-containing *shGAG* from *mU1 EVD* is translated as a larger, intron-containing, Gag protein as shown in the longer exposure of the original Supplementary Figure 5B. However, lack of stop codon likely results in protein turnover yielding lower Gag levels.
- We also explained in the original text (this is now on line 239 onward) that " *Impeding U1 binding and its inhibitory action on PCPA causes a complete lack of splicing in EVDmU1 (Fig.3C-D). This generates short unspliced transcripts, alternatively terminated at the cognate shGAG terminator or at an intronic cryptic site previously mapped by 3' RACE (Oberlin et al, 2017), both detected here by northern analysis (Fig.3C-E, Fig.S5A). Both alternatively terminated transcripts likely undergo translation, albeit largely unproductively*

(Fig.3F), because low levels of cryptic GAG translation products were detectable in rdr6 compared to WT (Fig.S5B)".

To help the reader, we have now added the anti-gag western blot formerly depicted in Figure S5B to the main Figure 3, where it is now panel F.

3. The authors have shown that FL GAG-Pol is expressed at much higher levels (~9x more in WT?) than shGAG. How does this factor into interpretation of the role of nuclear sequestration of FL-GAG in differential translation? A back of the envelope calculation suggests that there is still more FL-GAG in the cytoplasm then there is shGAG. To me, that suggests that there are perhaps other mechanisms that also contribute to the differential translation.

Although we believe our own calculations indicate that *shGAG* is more abundant than *flGAG-POL* in the cytosol, indeed other mechanisms can affect differential translation, including that *GAG-POL* might have different translation re-initiation dynamics given that the polyprotein ORF is larger than that of *shGAG.* In fact it is precisely for this reason that we were cautious in our interpretation and wrote (now on line 351 onward):" *This is likely contributing to the disproportionate translation of shGAG over flGAG-POL**, although we do not exclude the involvement of other processes***". Taking the reviewer's valid comment into consideration we have added an example of such "other process" on line 352 onward: "*For instance, in animal cells, exon-junction complex (EJC) deposition enhances translation of mRNAs even when tethered to intron-less transcripts (Nott et al, 2004). This could also contribute to enhance translation of the splicing-dependent shGAG isoform".*

4. The manuscript cites studies in which splicing promoters nuclear export. Are the mU1/intron deleted GAG exported efficiently to the cytoplasm?

mU1 EVD produces both unspliced *shGAG* and *flGAG-POL*. Although this would intuitively suggest that both transcripts remain nuclear, the literature indicates that U1 binding is required for nuclear retention such that mRNAs lacking U1 binding sites would be exported to the cytoplasm the same way intron-less transgene mRNAs do. It is therefore normal for mU1 *flGAG-POL* to exit the nucleus although this was not tested formally by us but is a planned experiment for a follow up. Likewise intron-less *EVD* only generates *shGAG* (Figure 3) and produces high levels of protein, indicating efficient nuclear export and translation.

5. The ribosome stalling data looks solid and perhaps could be the end of the paper. The conclusions that can be drawn from observations of ribothrypsis are vague and makes it difficult for the reader to wrap their head around the result. For example, Fig S11a suggests that the transcripts that are breaking don't have poly(A) tails. However, on Page 10, lines 188-190, the authors conclude that polyadenylation defects don't contribute to the PTGS. Its of course possible that small RNAs are being produced from a small subset of non-polyadenylated shGAG

The text (lines 183-186, previously 188-190) and experiments presented in Supplementary Figure 3 were intended to rule out that a bulk of non-polyadenylated transcripts, generated as a result of general aberrant transcription (i.e. way upstream translation) might be the cause of siRNA production from *shGAG* as this has been previously shown to stimulate RDR6 activity during transgene PTGS (Luo & Chen, 2007, The Plant Cell). Figure S3 rules out that this is the case since the PolyA(-) RNA level does not increase in *rdr6* mutants. In fact, an intact PolyA tail **is expectedly required** for translation and, hence, **TdS**. However, as a result of stalling a (probably minute, as suggested by the reviewer) subfraction of cleaved RNA is 5'OH and likely also lacks a polyA tail. Given that PolyA tails inhibit RDR6 activity (Baeg *et al*., 2017, Nature Plants), the fact that *shGAG* and *shGFP* siRNAs are translation-dependent and that de-adenylation of mRNAs takes place during co-translational RNA decay and other RNA quality control processes associated with defective translation (Collart & Weiss, 2019, NAR; Shcoenberg & Maquat, 2012, Nat Rev Genet); we reinvestigated at higher resolution, using acrylamide gel electrophoresis, the presence of *shGAG* derived PolyA(-) RNA fragments. We only suggest in the text that those might be the substrates for RDR6 as we also have not tested the concurrence on a same RNA molecule of lack of PolyA and presence of 5'OH ends (lines 536-541)

Minor Issues

1. I would suggest to the authors to add all gel (in their whole and unspliced form to supplements).
It might have escaped the reviewer's attention, but as indicated in the "data access" section of the original manuscript, we had deposited and made publicly available all raw data (including all uncropped gel/blot pictures) in the European repository Zenodo (www.zenodo.org) under the DOI: 10.5281/zenodo.5564305.

2. Figure S3 is hard to interpret/understand. In S3a, what are the two bars for each genotype?

As originally specified in the legend, one bar is for polyA+ and the other for polyA-.

What are the error bars?

As originally explained in the legend and detailed in the material & methods section, the error bars are standard errors of the means.

Were technical replicates done for the qPCR?

Yes, as explained in the material & methods section, three technical replicates were performed for each bio-rep.

I also suggest that the authors show both duplicates as separate graphs instead of clubbing them and calculating standard error. I am not sure its good practice to calculate standard error for N=2.

We agree with the referee that two bio-reps is suboptimal, but given our approach of splitting the sample in PolyA+/- from two independent lines (bulk of 10 plants each as depicted in material & methods) that is the best we could to fit all samples in a single qPCR plate. Additionally, we have RNA gel blot analyses of independent samples also probed against *EVD* but also *18S* and *ACT2*. We originally did not incorporate them in the final figure as they were somewhat redundant, but since they provide a third replicate, they are now depicted as panel B and C in the revised figure S3.

3. Fig S3a suggests that there was low rRNA in the non-Poly(a) fraction in rdr6 relative to wild-type . Does this represent something biological variation or is it technical variation? If its technical variation, is the difference so huge that it suggests some sort of failure to isolate the non-PolyA fraction in rdr6 and does this mean that you cant draw meaningful conclusions about the shgag levels in non-poly(A)?

We believe these are technical variations since the RNA gel blots show no difference in efficiency of polyA-isolation.

Is it better instead to measure tail length of shgag in wild-type and rdr6? If your model is right, then tail length should be similar between wt and rdr6. However, if non poly(A) shgag is increased in levels, then tail length can reduce.

This is a good point from the referee, which we indeed tried to tackle previously. In order to map in parallel 5' ends and PolyA tail lengths, we did attempt circular (c)RT-PCR. The technique consists in self ligating 5'- and 3'- of RNA molecules to PCR amplify the junction after cDNA conversion (Lange *et al.*, 2008, Mol Cell Biol). Unfortunately, we mostly obtained RNA concatemers that resulted from PCR artifacts. The few sequenced clones did not show differences in PolyA tail length. However, the number of successful clones was too low to draw any robust conclusion, unfortunately.

4. In Figure 2, it might be useful to indicate the position of the PCPA signal.

The PCAP site is now indicated on Figure 2.

5. In Figure S4B, the fraction of small RNAs that are intron spanning (i.e exon-exon) vs ones that are exon-intron might be important. This was also missing in Oberlin et al (2017). Its unclear if its just a handful of junction spanning reads that are found or its actually quite a large number and represent something meaningful.

We agree with the reviewer that this is a relevant matter, which we did not further develop in this manuscript because we had previously shown in Oberlin *et al.*, 2017 (Figure 7A, see image below) that the amount of junction reads was as abundant as that of those mapping to the exon, thus further supporting that spliced *shGAG* was the only source of siRNAs. We concluded in the previous manuscript by suggesting that this had to be further explored which we have done in the present work.

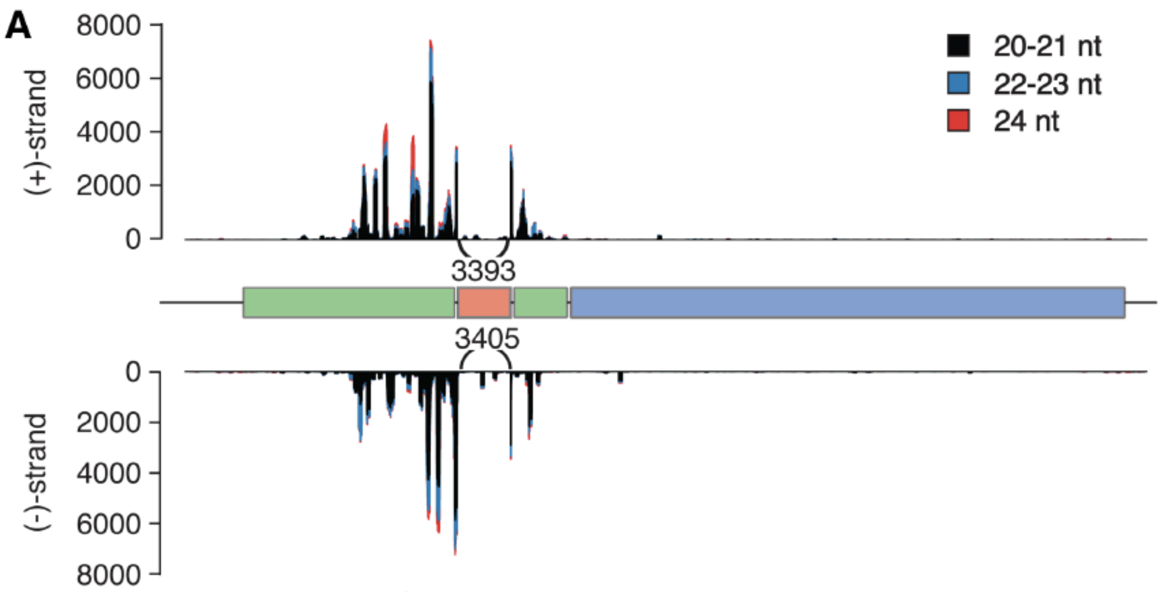

EVD 20- to 24-nt siRNA profile mapped with a splice-aware mapping software. Arcs display previously unmapped splice junction siRNA reads.

6. Overall, I feel like the manuscript can be greatly improved by reorganizing the paper a bit and by proof reading (for example, I suspect that Page10, line 188 may be missing "out" from " We ruled that ....") and better sentence construction. It's a complicated topic and better organization and language will make this manuscript a more pleasurable read. In other examples, can you change the colors in Fig3D? The splice and unspliced GAG are colored using a scheme which makes it hard for some people to distinguish. See also heading on line 351, page 16. It probably should read "unlikely to trigger".

- We did proofread as much as possible. Those specific mistakes have been corrected (“We ruled that” by “We ruled out that…”, now on line 184).
- The “unlikely triggers” by “is unlikely to trigger” on the heading in line 348 was also changed.
- We have now tried to decongestion the manuscript quite considerably;
- Figure 3D color scheme has been changed to grayscale so spliced and unspliced are easier to distinguish. In addition, as Figure 1D had the same color scheme, it has also been turned into grayscale.

Dear Olivier,

Thank you for the submission of your revised manuscript. We have now received the enclosed reports from referees 1 and 2. Given that the referees are not in agreement (referee 2 feels that the manuscript is better suited to publication in a more specialized journal), I have contacted an advisor, whose comments are also below. The advisor supports the publication of your work, if the overstatements are toned down, and we can therefore offer to publish your manuscript.

I think it is in both our interest to publish your paper online still this year. In order to do so, we need to receive all final and complete files latest on the 6th of December. I will go through your source data over the next days and let you know in case any corrections are required.

Please carefully address the advisor's comments in the final manuscript text.

A few other editorial requests also need to be addressed:

- Please add up to 5 keywords to the manuscript file.

- The "section B statistics" in the author checklist needs to be completed. You have applied statistics throughout your figures. Please upload a new and completed checklist with your final manuscript.

- Please upload all figures in separate files, i.e. one file per figure.

- Please add the legends of the Appendix figures directly below the figures and remove the legends from the main manuscript file. The Appendix also requires a table of content that lists all figures and page numbers, please add.

- I attach to this email a related ms file with comments by our data editors. Please address all comments in the final manuscript.

EMBO press papers are accompanied online by A) a short (1-2 sentences) summary of the findings and their significance, B) 2-3 bullet points highlighting key results and C) a synopsis image that is exactly 550 pixels wide and 200-600 pixels high (the height is variable). You can either show a model or key data in the synopsis image. Please note that text needs to be readable at the final size. Please send us this information along with the final manuscript

I look forward to seeing a final version of your manuscript as soon as possible. Please use this link to submit your revision: https://embor.msubmit.net/cgi-bin/main.plex

Kind regards,
Esther

Esther Schnapp, PhD
Senior Editor
EMBO reports

Referee #1:

After careful reading of the revised ms and of the rebuttal letter, I am totally satisfied. Not only the authors have addressed my comments, but they have produced additional data, which, if they do not explain what causes ribosome stalling on EVD, at least exclude a role for consecutive proline-glycine amino acids or G-quadruplexes at or upstream the ribosome stalling site. In addition, the authors have paid particular attention to the comments of the two other reviewers and were very cautious when drawing conclusions from their results and discussing their model in the light of other recent publications.

Referee #2:

The authors have carefully addressed my previous concerns. Accordingly, the gap between the description/interpretation and the actual data has now been reduced, while the manuscript has been significantly weakened. The abstract and introduction still read as if this is a general phenomenon (it may well be, but the current data is not strong enough to support the generality) and should be appropriately revised before publication.

Advisor's comments:

In my opinion, this work reports novel and very interesting results that advance significantly our understanding of how the active retrotransposon EVADE triggers the production of siRNAs in A. thaliana.

Although previous works linked ribosome stalling on siRNAs production (e.g. Oberlin et al 2017, Kim et al 2021), this work goes one step further and dissects the different pathways involved in the process. In particular, they show that splicing, cytosolic accumulation, and unconventional 5'OH breakage of shGAG-derived RNAs a required for the production of EVADE-derived siRNAs. Thus, this work is original and relevant.

Notwithstanding, I do agree with Referee 2 comment that it is impossible to generalize these observations to other TEs. Indeed, and as the authors recognize in Lines 272-276, ribosomal stalling at EVD mRNA is a very rare event. Also, in Lines 249-250 the authors point that only EVADE spawns detectable RDR6-dependent shGAG siRNAs. More generally, there is no general association between polysomal engagement of TEs and the production of RDR6 dependent siRNAs (Figure 4). Thus, Abstract's (Lines 20-21) and introduction's (Lines 135-136) conclusions are overstated and must be qualified.

In conclusion, I fully support the publication of this article, provided the authors tone down their generalizations in the abstract and introduction section.

The authors have addressed all minor editorial requests.

2nd Revision - Editorial Decision 6th Dec 2021

Prof. Olivier Voinnet
ETH Zurich
Biology
Swiss Federal Institute of Technology Zurich
Universitätstrasse 2
Zurich, Zürich 8092
Switzerland

Dear Olivier,

I am very pleased to accept your manuscript for publication in the next available issue of EMBO reports. Thank you for your contribution to our journal.

At the end of this email I include important information about how to proceed. Please ensure that you take the time to read the information and complete and return the necessary forms to allow us to publish your manuscript as quickly as possible.

As part of the EMBO publication's Transparent Editorial Process, EMBO reports publishes online a Review Process File to accompany accepted manuscripts. As you are aware, this File will be published in conjunction with your paper and will include the referee reports, your point-by-point response and all pertinent correspondence relating to the manuscript.

If you do NOT want this File to be published, please inform the editorial office within 2 days, if you have not done so already, otherwise the File will be published by default [contact: emboreports@embo.org]. If you do opt out, the Review Process File link will point to the following statement: "No Review Process File is available with this article, as the authors have chosen not to make the review process public in this case." Please note that the author checklist will still be published even if you opt out of the transparent process.

Thank you again for your contribution to EMBO reports and congratulations on a successful publication. Please consider us again in the future for your most exciting work.

Best regards,
Esther

Esther Schnapp, PhD
Senior Editor
EMBO reports

****

THINGS TO DO NOW:

You will receive proofs by e-mail approximately 2-3 weeks after all relevant files have been sent to our Production Office; you should return your corrections within 2 days of receiving the proofs.

Please inform us if there is likely to be any difficulty in reaching you at the above address at that time. Failure to meet our deadlines may result in a delay of publication, or publication without your corrections.

All further communications concerning your paper should quote reference number EMBOR-2021-53400V3 and be addressed to emboreports@wiley.com.

Should you be planning a Press Release on your article, please get in contact with emboreports@wiley.com as early as possible, in order to coordinate publication and release dates.

EMBO PRESS

YOU MUST COMPLETE ALL CELLS WITH A PINK BACKGROUND ↓

PLEASE NOTE THAT THIS CHECKLIST WILL BE PUBLISHED ALONGSIDE YOUR PAPER

Corresponding Author Name: Olivier Voinnet
Journal Submitted to: EMBO reports
Manuscript Number: EMBOR-2021-53400V2

USEFUL LINKS FOR COMPLETING THIS FORM

http://www.antibodypedia.com
http://1degreebio.org
http://www.equator-network.org/reporting-guidelines/improving-bioscience-research-repor
http://grants.nih.gov/grants/olaw/olaw.htm
http://www.mrc.ac.uk/Ourresearch/Ethicsresearchguidance/Useofanimals/index.htm
http://ClinicalTrials.gov
http://www.consort-statement.org
http://www.consort-statement.org/checklists/view/32-consort/66-title
http://www.equator-network.org/reporting-guidelines/reporting-recommendations-for-tumo
http://datadryad.org
http://figshare.com
http://www.ncbi.nlm.nih.gov/gap
http://www.ebi.ac.uk/ega
http://biomodels.net/
http://biomodels.net/miriam/
http://jjj.biochem.sun.ac.za
https://osp.od.nih.gov/biosafety-biosecurity-and-emerging-biotechnology/
http://www.selectagents.gov/

Reporting Checklist For Life Sciences Articles (Rev. June 2017)

This checklist is used to ensure good reporting standards and to improve the reproducibility of published results. These guidelines are consistent with the Principles and Guidelines for Reporting Preclinical Research issued by the NIH in 2014. Please follow the journal’s authorship guidelines in preparing your manuscript.

A- Figures
1. Data
The data shown in figures should satisfy the following conditions:

- the data were obtained and processed according to the field’s best practice and are presented to reflect the results of the experiments in an accurate and unbiased manner.
- figure panels include only data points, measurements or observations that can be compared to each other in a scientifically meaningful way.
- graphs include clearly labeled error bars for independent experiments and sample sizes. Unless justified, error bars should not be shown for technical replicates.
- if n< 5, the individual data points from each experiment should be plotted and any statistical test employed should be justified
- Source Data should be included to report the data underlying graphs. Please follow the guidelines set out in the author ship guidelines on Data Presentation.

2. Captions

Each figure caption should contain the following information, for each panel where they are relevant:

- a specification of the experimental system investigated (eg cell line, species name).
- the assay(s) and method(s) used to carry out the reported observations and measurements
- an explicit mention of the biological and chemical entity(ies) that are being measured.
- an explicit mention of the biological and chemical entity(ies) that are altered/varied/perturbed in a controlled manner.
- the exact sample size (n) for each experimental group/condition, given as a number, not a range;
- a description of the sample collection allowing the reader to understand whether the samples represent technical or biological replicates (including how many animals, litters, cultures, etc.).
- a statement of how many times the experiment shown was independently replicated in the laboratory.
- definitions of statistical methods and measures:
  - common tests, such as t-test (please specify whether paired vs. unpaired), simple χ^2 tests, Wilcoxon and Mann-Whitney tests, can be unambiguously identified by name only, but more complex techniques should be described in the methods section;
  - are tests one-sided or two-sided?
  - are there adjustments for multiple comparisons?
  - exact statistical test results, e.g., P values = x but not P values < x;
  - definition of ‘center values’ as median or average;
  - definition of error bars as s.d. or s.e.m.

Any descriptions too long for the figure legend should be included in the methods section and/or with the source data.

**In the pink boxes below, please ensure that the answers to the following questions are reported in the manuscript itself. Every question should be answered. If the question is not relevant to your research, please write NA (non applicable).
We encourage you to include a specific subsection in the methods section for statistics, reagents, animal models and human subjects.**

B- Statistics and general methods

Please fill out these boxes ↓ (Do not worry if you cannot see all your text once you press return)

1.a. How was the sample size chosen to ensure adequate power to detect a pre-specified effect size?	Bulks of at least 10 individual plants were used per biological replicate. At least two/three independent transgenic lines/segregating populations were used as biological replicates.
1.b. For animal studies, include a statement about sample size estimate even if no statistical methods were used.	NA
2. Describe inclusion/exclusion criteria if samples or animals were excluded from the analysis. Were the criteria pre-established?	No samples were excluded from analysis with the exception of qPCR technical replicates if they deviated more than two PCR cycles from the other two technical replicates within each biological replicate.
3. Were any steps taken to minimize the effects of subjective bias when allocating animals/samples to treatment (e.g. randomization procedure)? If yes, please describe.	Harvest of samples from at least 10 plants within each genotype (biological replicate) was randomized. No plant was included or excluded from pooling, unless they failed to germinate.
For animal studies, include a statement about randomization even if no randomization was used.	NA
4.a. Were any steps taken to minimize the effects of subjective bias during group allocation or/and when assessing results (e.g. blinding of the investigator)? If yes please describe.	allocation of plants to bulks for sampling was based solely o their genotype. Blinding of the investigator was difficult due to experimental design, but all results have been discussed with multiple authors to avoid bias of interpretation.
4.b. For animal studies, include a statement about blinding even if no blinding was done	NA
5. For every figure, are statistical tests justified as appropriate?	Yes, indicated in figure legends and methods section
Do the data meet the assumptions of the tests (e.g., normal distribution)? Describe any methods used to assess it.	For. NGS data, normality tests are included in standard analysis pipelines. For qPCR data normality had to be assumed as the sample size (3 biological replicates) are not enough to adress normality. In such cases two-sided t-tests were used as they produce more conservative p-values without assuming differences between two samples go in a given direction.
Is there an estimate of variation within each group of data?	Yes, indicated in figure legends and methods section

Is the variance similar between the groups that are being statistically compared?	Yes, although for qPCR data variance could only be assesd for the reference genes present in all samples/conditions tested but had to be assumed to be equal within genotypes/samples as only three biological replicates were used.

C- Reagents

6. To show that antibodies were profiled for use in the system under study (assay and species), provide a citation, catalog number and/or clone number, supplementary information or reference to an antibody validation profile. e.g., Antibodypedia (see link list at top right), 1DegreeBio (see link list at top right).	All antibodies and reagents catalog number and detailed information is provided in Methods section
7. Identify the source of cell lines and report if they were recently authenticated (e.g., by STR profiling) and tested for mycoplasma contamination.	NA

* for all hyperlinks, please see the table at the top right of the document

D- Animal Models

8. Report species, strain, gender, age of animals and genetic modification status where applicable. Please detail housing and husbandry conditions and the source of animals.	NA
9. For experiments involving live vertebrates, include a statement of compliance with ethical regulations and identify the committee(s) approving the experiments.	NA
10. We recommend consulting the ARRIVE guidelines (see link list at top right) (PLoS Biol. 8(6), e1000412, 2010) to ensure that other relevant aspects of animal studies are adequately reported. See author guidelines, under 'Reporting Guidelines'. See also: NIH (see link list at top right) and MRC (see link list at top right) recommendations. Please confirm compliance.	NA

E- Human Subjects

11. Identify the committee(s) approving the study protocol.	NA
12. Include a statement confirming that informed consent was obtained from all subjects and that the experiments conformed to the principles set out in the WMA Declaration of Helsinki and the Department of Health and Human Services Belmont Report.	NA
13. For publication of patient photos, include a statement confirming that consent to publish was obtained.	NA
14. Report any restrictions on the availability (and/or on the use) of human data or samples.	NA
15. Report the clinical trial registration number (at ClinicalTrials.gov or equivalent), where applicable.	NA
16. For phase II and III randomized controlled trials, please refer to the CONSORT flow diagram (see link list at top right) and submit the CONSORT checklist (see link list at top right) with your submission. See author guidelines, under 'Reporting Guidelines'. Please confirm you have submitted this list.	NA
17. For tumor marker prognostic studies, we recommend that you follow the REMARK reporting guidelines (see link list at top right). See author guidelines, under 'Reporting Guidelines'. Please confirm you have followed these guidelines.	NA

F- Data Accessibility

18: Provide a "Data Availability" section at the end of the Materials & Methods, listing the accession codes for data generated in this study and deposited in a public database (e.g. RNA-Seq data: Gene Expression Omnibus GSE39462, Proteomics data: PRIDE PXD000208 etc.) Please refer to our author guidelines for 'Data Deposition'.<br><br>Data deposition in a public repository is mandatory for:<br>a. Protein, DNA and RNA sequences<br>b. Macromolecular structures<br>c. Crystallographic data for small molecules<br>d. Functional genomics data<br>e. Proteomics and molecular interactions	Data availability section is found within Methods section
19. Deposition is strongly recommended for any datasets that are central and integral to the study; please consider the journal's data policy. If no structured public repository exists for a given data type, we encourage the provision of datasets in the manuscript as a Supplementary Document (see author guidelines under 'Expanded View' or in unstructured repositories such as Dryad (see link list at top right) or Figshare (see link list at top right).	All NGS and Raw data used in this file have been deposited in public repositories as indicated in the data availability section found in Methods
20. Access to human clinical and genomic datasets should be provided with as few restrictions as possible while respecting ethical obligations to the patients and relevant medical and legal issues. If practically possible and compatible with the individual consent agreement used in the study, such data should be deposited in one of the major public access-controlled repositories such as dbGAP (see link list at top right) or EGA (see link list at top right).	NA
21. Computational models that are central and integral to a study should be shared without restrictions and provided in a machine-readable form. The relevant accession numbers or links should be provided. When possible, standardized format (SBML, CellML) should be used instead of scripts (e.g. MATLAB). Authors are strongly encouraged to follow the MIRIAM guidelines (see link list at top right) and deposit their model in a public database such as Biomodels (see link list at top right) or JWS Online (see link list at top right). If computer source code is provided with the paper, it should be deposited in a public repository or included in supplementary information.	NA

G- Dual use research of concern

22. Could your study fall under dual use research restrictions? Please check biosecurity documents (see link list at top right) and list of select agents and toxins (APHIS/CDC) (see link list at top right). According to our biosecurity guidelines, provide a statement only if it could.	No dual research can be applied to this research
