# Supplementary material for: Innate, translation‐dependent silencing of an invasive transposon in Arabidopsis
Source: EMBO Rep. 2021 Dec 21;23(3):e53400. doi: 10.15252/embr.202153400 (PMC8892269; doi:10.15252/embr.202153400)
Supplement: Supplementary file 2 — Source Data for Appendix [file EMBR-23-e53400-s008.zip › embr202153400-sup-0007-SDataFigEV/embr202153400-sup-0006-AppendixFigS5.zip!/Appendix Figure S5/S5F/Commassie_cyto_nuclear.pdf]

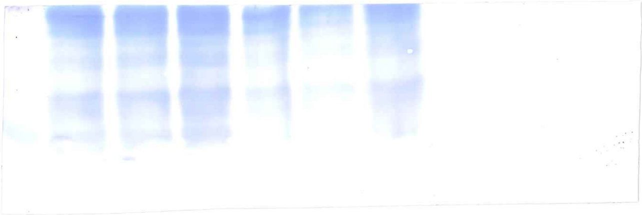

H3
