# Supplementary material for: Innate, translation‐dependent silencing of an invasive transposon in Arabidopsis
Source: EMBO Rep. 2021 Dec 21;23(3):e53400. doi: 10.15252/embr.202153400 (PMC8892269; doi:10.15252/embr.202153400)
Supplement: Supplementary file 2 — Source Data for Appendix [file EMBR-23-e53400-s008.zip › embr202153400-sup-0007-SDataFigEV/embr202153400-sup-0007-AppendixFigS6.zip!/Supp Figure 6/6G/PlatePlan_rdr6F2_ExpressionLevel.pdf]

Plate: rdr6 F2, EVD Expression Levels

Plate plan

	1	2	3	4	5	6	7	8	9	10	11	12	13	14	15	16	17	18	19	20	21	22	23	24
A																								
B		Col-0	Col-0	Col-0		Col-0	Col-0	Col-0		Col-0	Col-0	Col-0		Col-0	Col-0	Col-0								
C																								
D		rdr6-12	rdr6-12	rdr6-12		rdr6-12	rdr6-12	rdr6-12		rdr6-12	rdr6-12	rdr6-12		rdr6-12	rdr6-12	rdr6-12								
E																								
F		F2_1+	F2_1+	F2_1+		F2_1+	F2_1+	F2_1+		F2_1+	F2_1+	F2_1+		F2_1+	F2_1+	F2_1+								
G																								
H		F2_1+	F2_1+	F2_1+		F2_1+	F2_1+	F2_1+		F2_1+	F2_1+	F2_1+		F2_1+	F2_1+	F2_1+								
I																								
J		F2_1-	F2_1-	F2_1-		F2_1-	F2_1-	F2_1-		F2_1-	F2_1-	F2_1-		F2_1-	F2_1-	F2_1-								
K																								
L		F2_2-	F2_2-	F2_2-		F2_2-	F2_2-	F2_2-		F2_2-	F2_2-	F2_2-		F2_2-	F2_2-	F2_2-								
M																								
N						H2O	H2O	H2O		H2O	H2O	H2O		H2O	H2O	H2O								
O																								
P																								

Primers

- ACT2 (columns 14–16)
- EVD-Spliced (columns 6–8)
- EVD-Unspliced (Full length) (columns 10–12)
- no RT, ACT2 primers (control) (columns 2–4)
